# Profluorescent verdazyl radicals – synthesis and characterization†
†Electronic supplementary information (ESI) available. CCDC 1051008–1051011. For ESI and crystallographic data in CIF or other electronic format see DOI: 10.1039/c5sc00724k



**DOI:** 10.1039/c5sc00724k

**Published:** 2015-06-05

**Authors:** David Matuschek, Steffen Eusterwiemann, Linda Stegemann, Carsten Doerenkamp, Birgit Wibbeling, Constantin G. Daniliuc, Nikos L. Doltsinis, Cristian A. Strassert, Hellmut Eckert, Armido Studer

**Affiliations:** a Institute of Organic Chemistry , Westfälische Wilhelms-Universität Münster , Corrensstrasse 40 , 48149 Münster , Germany . Email: studer@uni-muenster.de ; Tel: +49 (0)251-83-33291; b Institute of Physics and Center for Nanotechnology , Westfälische Wilhelms-Universität Münster , Heisenbergstrasse 11 , 48149 Münster , Germany . Email: ca.s@uni-muenster.de; c Institut für Physikalische Chemie , Westfälische Wilhelms-Universität Münster , Corrensstrasse 28/30 , 48149 Münster , Germany . Email: eckerth@uni-muenster.de; d Institut für Festkörpertheorie and Center for Multiscale Theory & Computation , Westfälische Wilhelms-Universität Münster , Wilhelm-Klemm-Straße 10 , 48149 Münster , Germany . Email: nikos.doltsinis@uni-muenster.de; e Instituto da Física em Sao Carlos , Universidade de Sao Paulo , Avenida Trabalhador Saocarlense 400 , Sao Carlos , SP 13590 , Brazil

## Abstract

The synthesis and characterization of various 6-oxo-verdazyl radicals and their diamagnetic styryl radical trapping products are presented. It is shown that styryl radical trapping products derived from *N*-phenyl verdazyls show fluorescence whereas the *N*-methyl congeners are non-fluorescent. In the parent *N*-phenyl verdazyls fluorescence is fully quenched which renders these compounds highly valuable profluorescent radical probes.

## Abstract 2

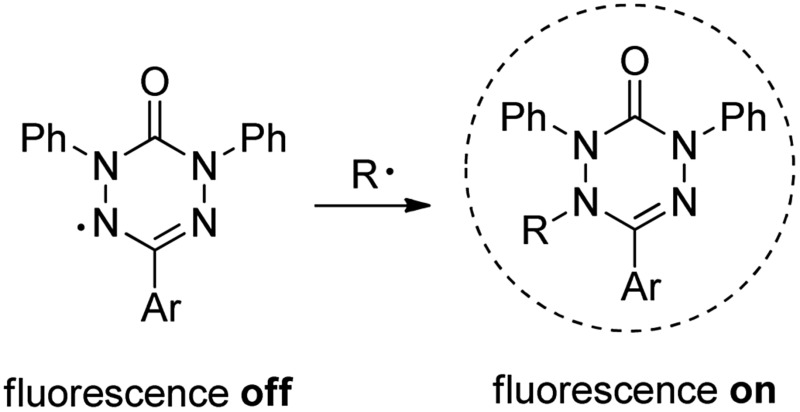
The synthesis and characterization of various 6-oxo-verdazyl radicals and their diamagnetic styryl radical trapping products are presented.

## 

6-Oxo-verdazyl radicals have been widely used as building blocks for construction of magnetic materials;^1^ however, there is no report on their application as profluorescent persistent radicals to be applied as radical probes. Along these lines, nitroxyl radicals conjugated with a fluorophore have been successfully used as profluorescent probes for detection of transient radicals.^2^ Whereas the nitroxide moiety in these conjugates fully quenches fluorescence of the chromophore, nitroxide trapping of a transient C-radical leads to the corresponding closed shell alkoxyamine thereby restoring fluorescence. This interesting property allows such profluorescent nitroxides to be used for radical detection. Transient radical intermediates play important roles in environmental chemistry, biological chemistry, polymer chemistry and organic synthesis.^3^ Therefore, development of methods for radical detection is of importance. Herein we present the synthesis of a series of novel verdazyl radicals and discuss their application as profluorescent persistent radicals for detection of C-centred radicals (Fig. 1).

**Fig. 1:**
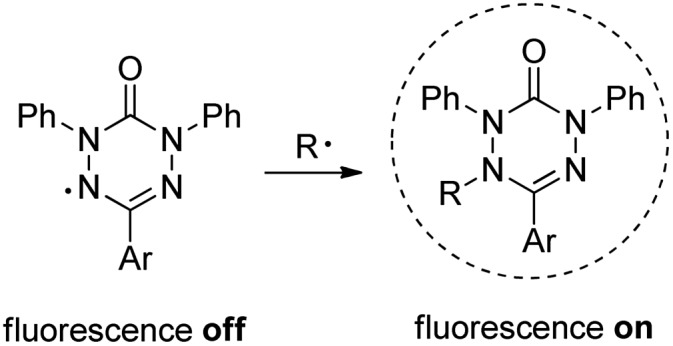
Profluorescent verdazyls as radical probes.

The 1,5-diphenyl-6-oxo-verdazyl radicals **8–15** were prepared using a modified literature procedure^4^ starting with 2,4-diphenylcarbonohydrazide (Table 1, for details, see ESI†). Radical precursor tetrazinan-3(2*H*)-ones **1–7** were obtained in moderate to good yields by acetalization of different aromatic aldehydes with 2,4-diphenylcarbonohydrazide. Oxidation with 1,4-benzoquinone afforded the verdazyl radicals **8–12**. In case of the anthracenyl-derivative **11**, oxidation was carried out without prior isolation of the acetal. In contrast to the established verdazyl syntheses of Barbier and Milcent *et al.*,^5,6^ the applied method allows readily varying the C3-substituent of the 1,5-diphenyl-6-oxo-verdazyl radicals. The bisverdazyls **13**, **14** and the trisverdazyl **15** were prepared in analogy starting with the corresponding bis and trisaldehydes, respectively. These bis- and tris-radicals are planar structures with up to 7 aromatic moieties, which exert strong π–π interactions. It is therefore not surprising that these interactions strongly reduce solubility of these radicals.

**Table 1:** Synthesis of various verdazyl radicals and the corresponding styryl radical trapping products (yields for the individual steps in brackets)^*a*^

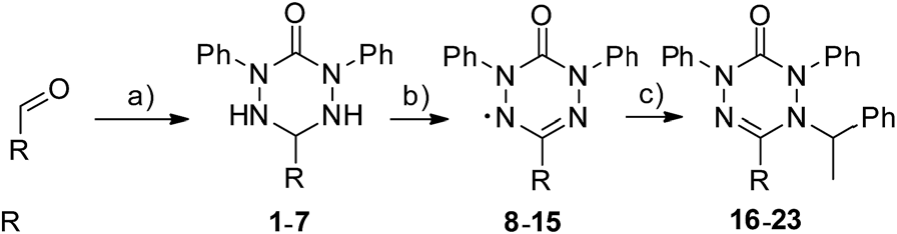			
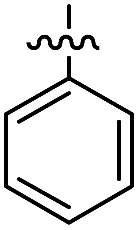	**1** (91%)	**8** (62%)	**16** (72%)
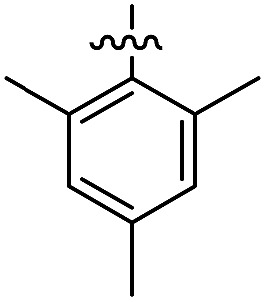	**2** (32%)	**9** (62%)	**17** (52%)
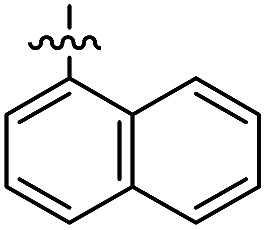	**3** (63%)	**10** (98%)	**18** (82%)
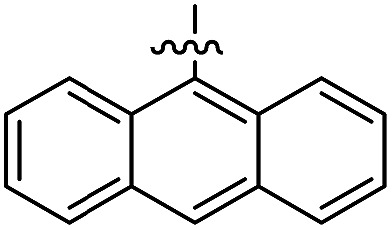	—	**11** (30%)	**19** (61%)
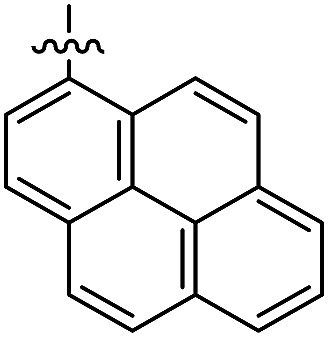	**4** (69%)	**12** (96%)	**20** (93%)
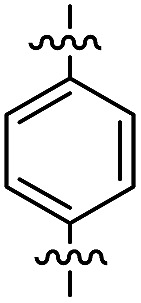	**5** (69%)	**13** (88%)	**21** (60%)
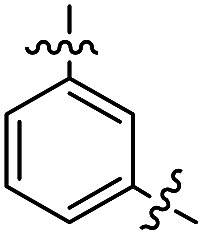	**6** (81%)	**14** (66%)	**22** (68%)
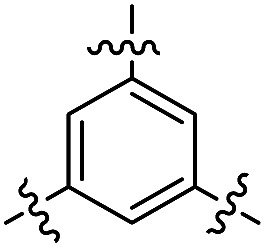	**7** (48%)	**15** (68%)	**23** (63%)

^*a*^Conditions: (a) 2,4-diphenylcarbonohydrazide (1.0 equiv.), 80 °C, 3–6 h; (b) 1,4-benzoquinone (1.5 equiv. – excess), DCM, 60 °C, 3 h; (c) (1-bromoethyl)-benzene (1.1 equiv.), Cu^II^OTf_2_ (2 mol%), BBBPY (4 mol%), benzene, 80 °C, 24 h.

By using the atom transfer coupling reaction^7^ with styryl bromide as a C-radical precursor, the diamagnetic tetrazin-3-ones **16–23** were obtained in good to excellent yields (Table 1).

The well soluble 1,5-diphenyl-6-oxo-verdazyl monoradicals **8–12** were analysed by UV/Vis spectroscopy (see ESI, Fig. S2 and S3†). Verdazyls **8–12** were also investigated by solution phase EPR spectroscopy (ESI, Fig. S1†) and the naphthyl **10** and anthracenyl radical **11** were further characterized by X-ray structure analysis (Fig. 2).

**Fig. 2:**
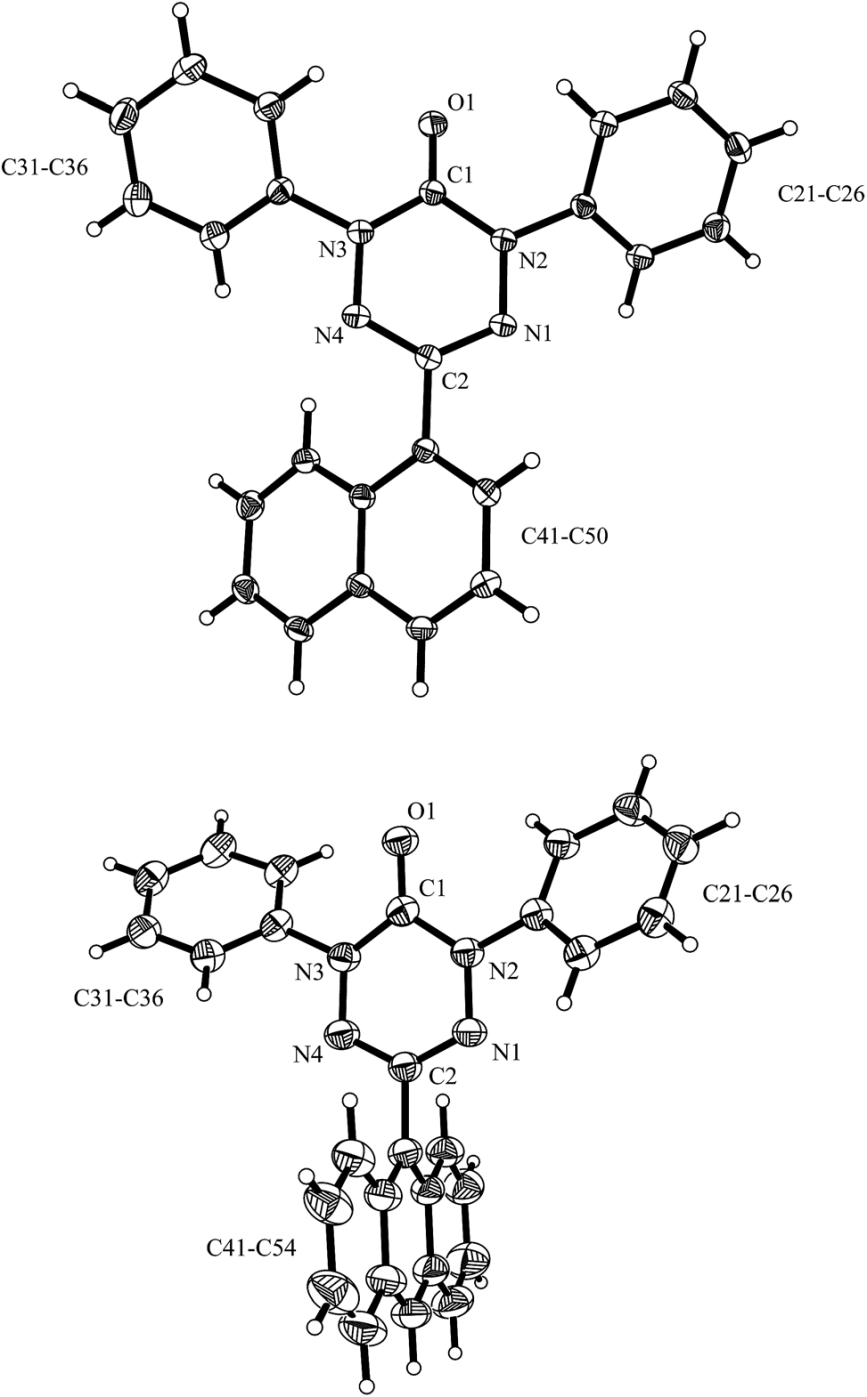
X-ray structure of **10** (top) and **11** (bottom) (thermals ellipsoids are shown with 30% probability).

In the UV/Vis spectra of these radicals we noted a characteristic UV band at 494–559 nm which is strongly influenced by the R-substituent of the verdazyl radicals (**8** (R = Ph): 559 nm; **9** (R = mesityl): 506 nm; **10** (R = 1-naphthyl): 528 nm; **11** (R = anthracenyl): 494 nm; **12** (R = pyrenyl): 510 nm). By looking at the X-ray structure of **11**, we noted the bulky anthracenyl moiety to lie with a torsion angle of 87.9(5)° nearly orthogonal with respect to the verdazylheteroarene plane (Fig. 2). However, in the naphthyl congener **10**, the torsion angle was only 47.2(2)° which allows for better conjugation of the R-arene-substituent with the heteroarene of the verdazyl. This conjugation leads to a red shift of the given UV band. In fact, the reported^8^ X-ray structure of the phenyl verdazyl **8** showed a small torsion angle (12.3°) which is well reflected by the red shifted UV-absorbance (559 nm). Hence the shift of the characteristic UV-band can be taken as a measure for the conjugation of the R-substituent with the verdazyl heteroarene.

This view is further corroborated by our DFT calculations in the gas phase. They confirm that **11** has a larger torsion angle (64°) than **10** (44°) and indeed predict a red-shifted absorption band of **10** at 524 nm. The same argument explains why the absorption spectrum of **9**, which also exhibits a pronounced torsion angle of 62°, is in a similar region as that of **11**. At the other extreme, the torsion angle of **8** is computed to be 9° in agreement with the X-ray structure^8^ and its theoretical absorption is red-shifted relative to **11** by 58 nm, close to the experimental shift of 65 nm.

EPR spectra of the 1 mM solutions of **8–12** in degassed dichloromethane showed the typical lineshapes (quintets of quintets) dominated by the isotropic hyperfine coupling with two distinct sets of two nitrogen atoms, consistent with data reported in the literature on other types of verdazyl radicals.^8–10^ The spectroscopy of the di- and tri-radicals was restricted by severe solubility limitations, however, this problem could be overcome by examining *p*-alkoxy-substituted analogues. Fig. 3 shows a typical result obtained on 1,3-bis(1,5-di-(4-octyloxy)phenyl-6-oxo-3-verdazyl)benzene 14* including the simulation. Table 2 gives a summary of all the *A*_iso_ values obtained.

**Fig. 3:**
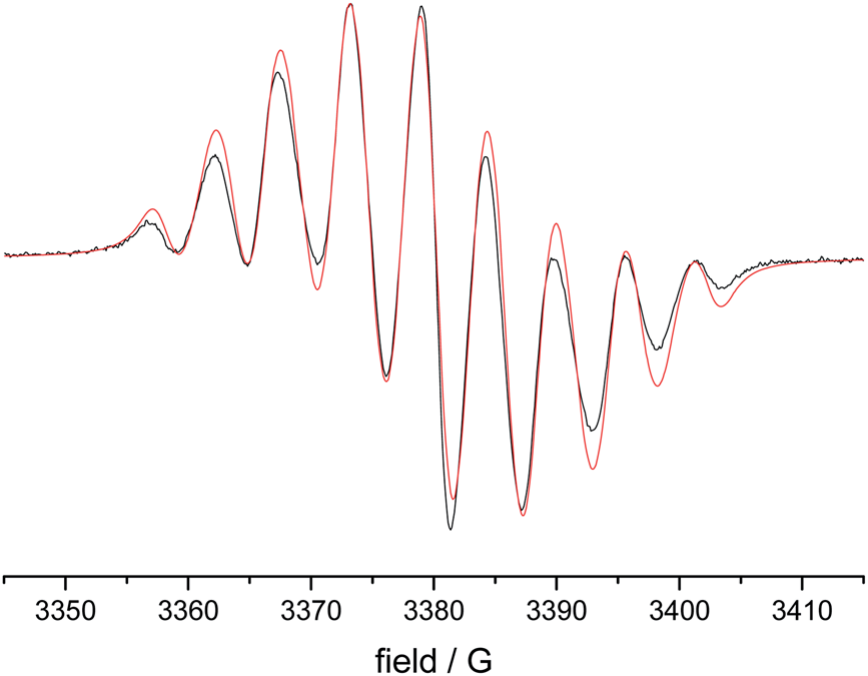
EPR spectrum of 1,3-bis(1,5-di-(4-octyloxy)phenyl-6-oxo-3-verdazyl)benzene (black)(**14**) and simulation (red).

**Table 2:** Summary of the *A*_iso_ values (in Gauss) and *g*-values for radicals **8–12** and **14**. Asterisks denote measurements on the *p*-alkoxy derivatives of **8** and **14**

	Sample	*A*(*N*-1,5)	*A*(*N*-2,4)	*g*-value
8*	1,5-Di-(4-methoxy)phenyl-3-phenyl-6-oxo-verdazyl*	4.866	6.198	2.00391
**9**	1,5-Diphenyl-3-mesityl-6-oxo-verdazyl	4.634	6.510	2.00399
**10**	1,5-Diphenyl-3-(1′-naphthyl)-6-oxo-verdazyl	4.632	6.479	2.00397
**11**	1,5-Diphenyl-3-(9′-anthracenyl)-6-oxo-verdazyl	4.691	6.441	2.00403
**12**	1,5-Diphenyl-3-(1′-pyrenyl)-6-oxo-verdazyl	4.689	6.434	2.00382
14*	1,3-Bis(1,5-di-(4-octyloxy)phenyl-6-oxo-3-verdazyl)benzene	3.858	4.930	2.00375

For the naphthyl **18** and anthracenyl derivate **19**, the structure was further characterized by X-ray analysis (for structures, see ESI, Fig. S35 and S36†). As for the verdazyl radicals, the anthracenyl moiety in tetrazin-3-one **19** has a far larger torsion angle with respect to the heterocycle (torsion angle = –67.4°) than the naphthyl substituent (17.0°). This is confirmed by our theoretical optimized gas phase structures, which exhibit torsion angles of –64° and 27°, respectively. A detailed comparison of X-ray and theoretical geometries can be found in the ESI (Table S2†).

In contrast to the structures of the parent verdazyl radicals **10** and **11**, where the heteroarene has a nearly planar structure due to the delocalization of the spin, the crystal structures of **18** and **19** reveal distortion of the tetrazin-3-one core moiety. Theoretical CNNC dihedral angles in the heterocycle range from 30° to 45° and show an asymmetry within the ring (see ESI, Table S2† for more details).

The well soluble, diamagnetic tetrazin-3-ones **16–25** were analysed by UV/Vis, steady-state and time-resolved fluorescence spectroscopy, the corresponding UV/Vis absorption spectra are given in the ESI (Fig. S4†). With increasing delocalization of the substituent (compounds **16**, **18** and **20**), a red-shift of the absorption maxima can be observed (Fig. S4†). Interestingly, compounds **17** and **19** significantly deviate from this trend, which goes along with their larger torsion angles that hinder an effective conjugation with the heteroarene. Nonetheless, between **17** and **19**, a clear trend can be observed regarding bathochromic absorption shifts and increasing delocalization. In all cases, however, the vertical S_0_–S_1_ transitions can be assigned to nearly pure HOMO–LUMO excitations, as indicated by our TD-DFT calculations. In fact, the LUMO is mainly centred on the aromatic moiety and appears increasingly stabilized with the degree of delocalization. This trend is illustrated in Fig. 4, which compares the shapes and energies of the LUMOs of compounds **16**, **18**, and **20** (the LUMOs of all compounds **16–23** are compared in Fig. S29 of the ESI†). On the other hand, the HOMO is mainly associated to the heterocycle, with minor contributions from the arene substituents in the cases of **19** and **20**. The latter possess a larger intrinsic π–π conjugation on the arene moiety that originates a significant admixture of the occupied arene-centred orbitals with the heteroarene-centred HOMO. Fig. 4 shows for the examples **16**, **18**, and **20** how the delocalization of the HOMO onto the arene substituent becomes more pronounced with increasing size of the arene. However, it can also be seen that this goes hand in hand with a withdrawal of electron density from the phenyl rings explaining the fact that the HOMO energy remains practically unchanged.

**Fig. 4:**
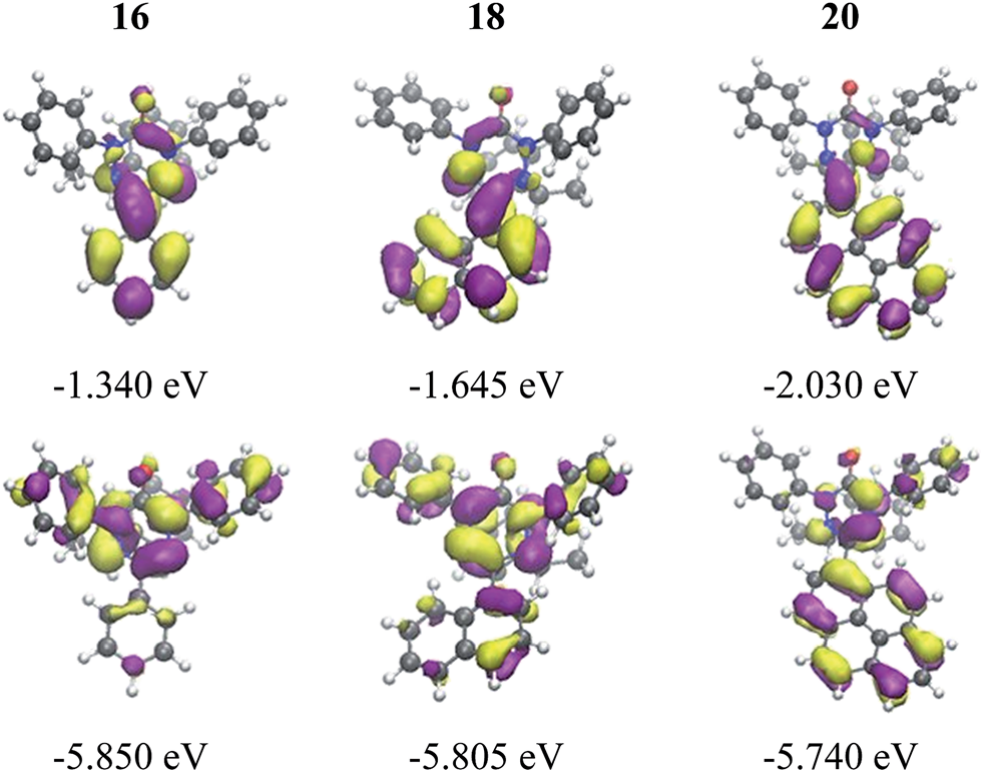
Visualization and energies of the HOMOs and LUMOs of **16**, **18**, and **20** calculated with the PBE0 functional and the 6-31G* basis set at the optimized ground state geometry.

In the series comprising compounds **16** and **21–23**, a maximized delocalization can be observed for the *para*-substitution pattern (**21**), which favors the most effective π-delocalization, compared to the *meta*-substitution pattern (**22**). Introduction of a further heteroarene unit on the *meta*′-position (**23**) enhances delocalization and bathochromic shifts, but without reaching the effective conjugation attained for **21**.

In contrast to the verdazyl radicals, all tetrazin-3-ones **16–25** display a measurable fluorescence. TD-DFT excited state geometry optimizations indicate that the radical species possess easily accessible conical intersections between the S_1_ and the S_0_ states, leading to a fast radiationless deactivation. For compounds **16**, **18** and **20**, the fluorescence emission maxima follow the same trends as described for their absorption spectra (*vide supra*), and the observed radiative rate constants are enhanced along with the π–π delocalization. Compounds **17** and **19**, which display comparable emission maxima despite the quite different intrinsic π–π delocalization of their arene substituents, do not follow these trends. This can be explained by their larger coplanar torsion angles that hinder an effective conjugation with the heteroarene. The rotational freedom of the phenyl moiety on compound **16** explains its high radiationless rate constant, as opposed to **17** for which the sterical hindrance of the methyl groups impose a fixed orthogonal conformation between the heterocycle and the substituent. The broad emission of compound **19** is due to numerous dynamically accessible conformations in the excited S_1_ state, as revealed our by *ab initio* molecular dynamics simulations (ESI, Section 5†). The emission shoulder at 660 nm, in particular, provides evidence that conformations with a small HOMO–LUMO gap are visited in the S_1_ state. This suggests the existence of energetically accessible pathways for radiationless decay *via* a conical intersection, which is confirmed by its relatively high non-radiative deactivation rate constant *k*_nr_ (see Table 3).

**Table 3:** Experimental and theoretical photophysical data of **16–25**. The theoretical data were calculated at the PBE0/6-31G* level including the effect of the acetonitrile solvent at the PCM level. The emission wavelengths of **16** and **19** were obtained by averaging over 10 configurations sampled randomly from an MD simulation in the S_1_ state. Quantum yields (*Φ*_fl_) were obtained by comparative method with quinine sulfate in 0.5 M H_2_SO_4_ (0.564)^11^ as a reference

	*λ*_Abs_ (nm)	*λ*_Abs,calc_ (nm)	*λ*_Em_ (nm)	*λ*_Em,calc_ (nm)	*τ* ^*a*^ (ns)	*φ*_fl_ (%)	*k*_r_/*k*_nr_ (10^5^ s^–1^)
**16**	332	339	397	438	1.43	0.27	19/6974
**17**	305	332	435	459	2.80	0.03	1/3570
**18**	336	366	484	493	0.88	1.29	147/11 217
**19**	394	405	441	449	0.87	0.39	45/11 449
**20**	368	407	515	528	0.62	1.88	303/15 826
**21**	368	396	503	531	2.80	5.96	213/3359
**22**	338	361	470	483	0.90	1.21	134/10 977
**23**	348	371	482	493	1.94	1.84	95/5060
**24**	253		—		—	—	—
24*	254		—		—	—	—
**25**	258		485		1.12	<0.01	—

^*a*^Amplitude-averaged lifetime.

A further corroboration for this model is provided by the 77 K measurements, showing a bathochromic shift from **17–20** that nicely correlates with the increasing π–π delocalization. However, compound **16** exhibits a similar red-shift as **18**, despite the different degree of π–π delocalization on its substituent, most likely due to the lack of rotational constraints that, in turn, enable a significant coplanarization of the phenyl unit with the heteroarene. Indeed, compound **17**, which bears two methyl groups that fix an orthogonal conformation between the arene and heteroarene rings, appears blue-shifted as compared to **16** and **18**.

Finally, we looked at the effect of the *N*-phenyl substituents in the tetrazin-3-ones on their fluorescence behavior. For this purpose, **24**, 24* and **25**, which are analogues of **16** and **23**, respectively, in which all *N*-phenyl groups have been replaced by *N*-methyl substituents, were successfully prepared (Fig. 6). To our surprise, neither **24** nor 24* showed any fluorescence at room temperature, clearly documenting the key importance of the *N*-phenyl groups on the fluorescence properties of these tetrazin-3-ones. Compound **25**, on the other hand, only displayed a weak, non-quantifiable luminescence. The calculated oscillator strength of compound **24** is indeed significantly smaller than that of **16**. The S_1_–S_0_ energy gap at the S_1_ minimum is smaller for **24** than for **16**, in line with the red-shift observed in the emission spectrum of **24** relative to **16** at 77 K (see Fig. 5c), also increasing the probability for nonradiative deactivation.

**Fig. 5:**
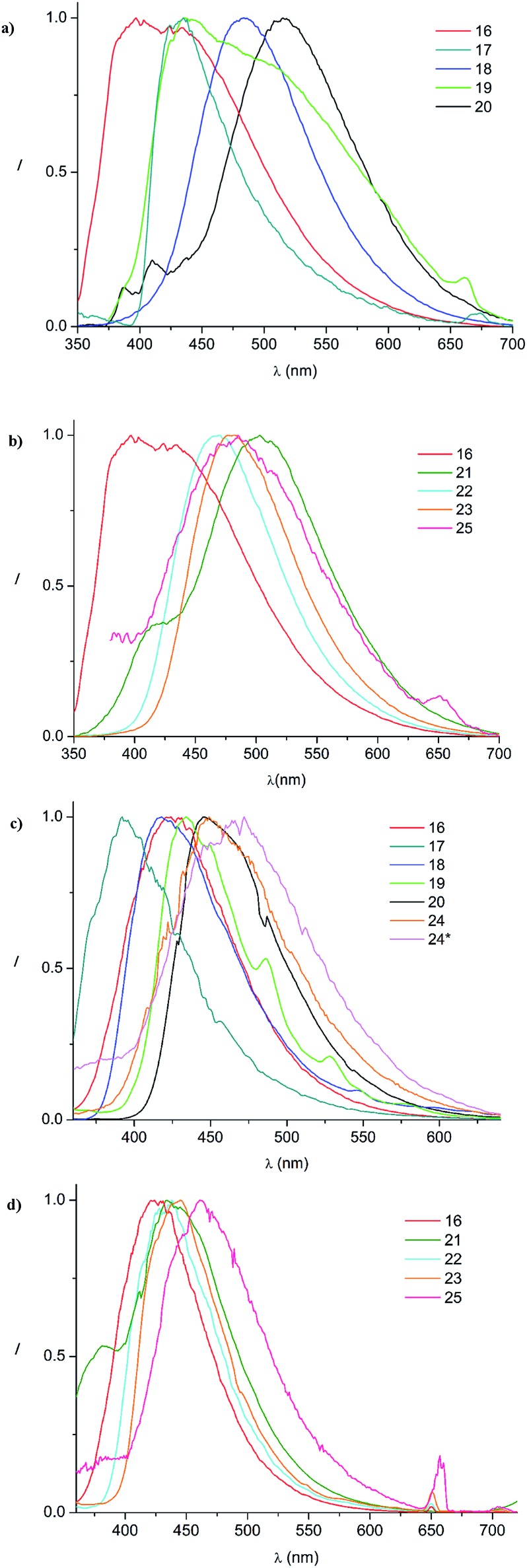
Normalized emission spectra in acetonitrile at room temperature ((a): **16–20**; (b): **16–25**) and at 77 K in a frozen glassy matrix of butyronitrile ((c): 16–24*; (d): **16–25**).

**Fig. 6:**
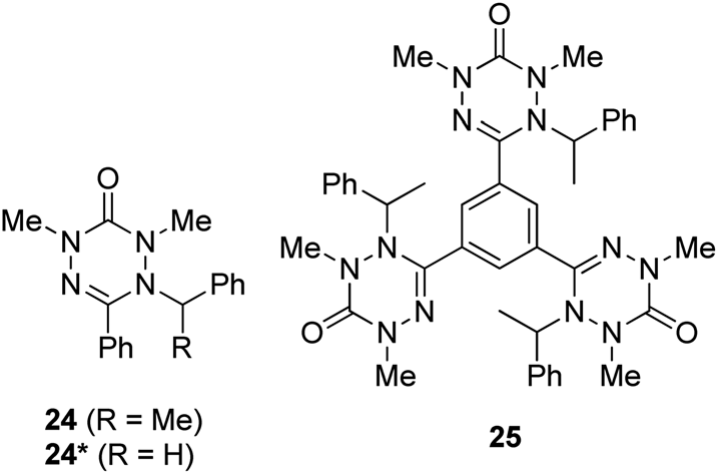
*N*-Methyl tetrazin-3-ones **24**, 24* and **25**.

In summary, we have presented an efficient synthesis for the preparation of various 1,5-diphenyl-6-oxo-verdazyl radicals. The method was also applied to the synthesis of bis- and tris-verdazyl radical systems. Verdazyls were characterized by UV/VIS and EPR spectroscopy. Analysis of the X-ray structure of two verdazyls allowed understanding the blue shift of the major UV band as a function of the C3-verdazyl substituent in the radicals. Importantly, we found that the verdazyl radicals do not have any fluorescence properties, which could be ascribed by TD-DFT calculations to the presence of S_1_–S_0_ conical intersections. However, the corresponding diamagnetic tetrazin-3-ones, readily obtained upon reacting the verdazyls with C-centred radicals are fluorescent. This profluorescent behavior renders these verdazyl valuable spin probes. We also found that the *N*-phenyl substituents at the tetrazin-3-one moieties are of key importance to obtain fluorescence.
