# Supplementary material for: Profluorescent verdazyl radicals – synthesis and characterization
Source: Chem Sci. 2015 Jun 5;6(8):4712–6. doi: 10.1039/c5sc00724k (PMC5667500; doi:10.1039/c5sc00724k)
Supplement: Supplementary file 1 [file SC-006-C5SC00724K-s001.pdf]

Electronic Supplementary Material (ESI) for Chemical Science.
This journal is © The Royal Society of Chemistry 2015

Supporting information

Profluorescent Verdazyl Radicals – Synthesis and Characterization

David Matuschek,[a] Steffen Eusterwiemann,[a] Linda Stegemann,[b] Carsten Doerenkamp,[c] Birgit Wibbeling,[a] Constantin G. Daniliuc,[a] Nikos L. Doltsinis,[*d] Cristian A. Strassert,[*b] Hellmut Eckert[*c,e], and Armido Studer[*a]

[a]*Organisch-Chemisches Institut, Westfälische Wilhelms-University Münster, Corrensstrasse 40, 48149 Münster, Germany*

E-mail: studer@uni-muenster.de

[b]*Physikalisches Institut and Center for Nanotechnology, Westfälische Wilhelms-Universität Münster, Heisenbergstrasse 11, D-48149 Münster, Germany*

E-mail: ca.s@uni-muenster.de

[c]*Institut für Physikalische Chemie, Westfälische Wilhelms-University Münster, Corrensstrasse 30, 48149 Münster, Germany*

E-mail: eckerth@uni-muenster.de

[d]*Institut für Festkörpertheorie and Center for Multiscale Theory & Computation, Westfälische Wilhelms-University Münster, Wilhelm-Klemm-Straße 10, 48149 Münster, Germany.*

E-mail: nikos.doltsinis@uni-muenster.de.

[e]*Instituto da Física em Sao Carlos, Universidade de Sao Paulo, Avenida Trabalhador Saocarlense 400, Sao Carlos, SP 13590, Brazil*

Table of contents

1 General information and methods 3

2 Experimental procedures 5

2.1 General procedures 5

2.2 Synthesis of carbonohydrazides 6

2.3 Synthesis of precursor compounds 8

2.4 Synthesis of tetrazinan-3-ones 9

2.5 Synthesis of verdazyl radicals 13

2.6 Synthesis of fluorescent tetrazin-3-ones 17

2.7 Synthesis of methyl substituted tetrazin-3-one derivatives 22

3 EPR spectra 26

4 Photophysical measurements 28

5 Calculation of absorption and emission spectra 38

6 X-Ray crystallographic data 42

7 ^{1}H and ^{13}C NMR spectra 46

8 References 68

1 General information and methods

^{1}H-NMR- and **^{13}C-NMR spectra** were recorded on an *AV-300*, *DPX-300* (^{1}H: 300 MHz;^{13}C 75 MHz) and an *AV-400* (^{1}H: 400 MHz; ^{13}C: 100 MHz) from *Bruker* at RT. The signals of the solvents were calibrated upon $CDCl_3$, CD_2Cl_2, DMSO-d_6 in ^{1}H NMR-spectra to the literature known value at δ = 7.260 ppm, 5.320 ppm, 2.500 pm, in ^{13}C NMR spectra relative to deuterated coupled signal at 77.160, 54.000, 39.520 ppm. The coupling constants *J* are given in Hz. The chemical shift in ppm are given relative to the resonance signal of the internal standard tetramethylsilane (δ = 0.00 ppm). Spectral splitting pattern are designated as *s* (singlet), *d* (doublet), *t* (triplet), *q* (quartet), *m* (multiplet) and *br* (broad signal). **Mass spectra** (HRMS - ESI) were recorded on a *Finnigan* MAT 4200S, *Bruker* Daltonics Micro-TOF, a *Micromass* Quatro LCZ (ESI), a *Bruker* LTQ Orbitrap XL and peaks are given in *m/z*. MALDI spectra were recorded with a *Bruker* Autoflex Speed TOF-MS in linear mode. **IR spectra** were recorded on a *Digilab* Varian 3100 FT-IR Excalibur Series equipped with a MKII Golden Gate Single Reflection ATR unit. Recorded IR signals are reported in wavelength (cm^{-1}) with the following abbreviation for the intensity of absorption: *s* = strong, *m* = medium, *w* = weak. **Melting points** (M.p.) were measured on a *Stuart* SMP-10 melting point apparatus and are uncorrected. **Elemental analysis** was performed at a *Vario EL III* of the company *Elementar-Analysensysteme GmbH*. **Steady-state emission spectra and luminescence lifetimes** were recorded on a FluoTime300 spectrometer from *PicoQuant* equipped with a 300 W ozone-free Xe lamp (250 – 900 nm), a 10 W Xe flash-lamp (250-900 nm, pulse width < 10μs) with repetition rates of 0.1 – 300 Hz, an excitation monochromator (Czerny-Turner 2.7 nm/mm dispersion, 1200 grooves/mm, blazed at 300 nm), diode lasers (pulse width < 80 ps) operated by a computer-controlled laser driver PDL-820 (repetition rate up to 80 MHz, burst mode for slow and weak decays), two emission monochromators (Czerny-Turner, selectable gratings blazed at 500 nm with 2.7 nm/mm dispersion and 1200 grooves/mm, or blazed at 1250 nm with 5.4 nm/mm dispersion and 600 grooves/mm), *Glan-Thompson* polarizers for excitation (Xe-lamps) and emission, a Peltier-thermostatized sample holder from *Quantum Northwest* (-40°C – 105°C), and two detectors, namely a PMA Hybrid 40 (transit time spread FWHM < 120 ps, 300 – 720 nm) and a R5509-42 NIR-photomultiplier tube (transit time spread FWHM 1.5 ns, 300-1400 nm) with external cooling (-80 °C) from *Hamamatsu*. Steady-state and fluorescence lifetimes were recorded in TCSPC mode by a PicoHarp 300 (minimum base resolution 4 ps). Emission and excitation spectra were corrected for source intensity (lamp and grating) by standard correction curves. Phosphorescence lifetimes were recorded by a NanoHarp 250 (minimum base resolution 32 ns)

in MCS mode. Lifetime analysis was performed using the commercial FluoFit software. The quality of the fit was assessed by minimizing the reduced chi squared function (χ2) and visual inspection of the weighted residuals and their autocorrelation. **Luminescence quantum yields** were obtained by using the comparative method with quinine sulfate in 0.5 M H_2SO_4 as the quantum yield standard (0.546).[1] **UV/Vis spectra** were measured at RT in DCM (~ 0.83 mM) in quartz glas cuvette (0.1 mm QS) with a *Varian* Cary 1Bio. **EPR measurements** were carried out on a Bruker X-Band spectrometer (9.48 GHz) and performed at ambient conditions with a sample concentration of 1 mM in dichloromethane, which was degassed with nitrogen. Depending on the sample 512 points at a center field of 338 mT, a sweep width of 10-13 mT and a sweep time of 41.94 s or 1024 points per spectra at a center field of 338 mT, a sweep width of 8-10 mT and a sweep time of 83.9 s were recorded. In every measurement a modulation amplitude of 0.1 mT with a modulation field of 100 MHz has been used. The microwave power attenuation level was 29db. 10 to 50 scans of each sample were added up.**Thin layer chromatography** was carried out on *Merck* silica gel 60 F254 plates; detection by UV (irradiation at 254 nm) or dipping into a solution of $KMnO_4$ (1.5 g), $NaHCO_3$ (5.0 g) in H_2O (400 mL), followed by heating. **Flash chromatography** (FC) was performed on silica gel (*Merck*-Si60 40 – 63 μm) with a pressure of 0.1 to 0.5 bar. Used eluents are given in parentheses. **Solvents:** All solvents for extraction and flash chromatography (FC) were distilled before use. Et_2O was distilled from K/Na, benzene was distilled from Na, THF was distilled from K, and CH_2Cl_2 was distilled from P_2O_5. All other solvents and chemicals were used as received from the suppliers (Alfa, Acros, Aldrich, ABCR, Fluka).

Techniques: All reactions involving air or moisture sensitive reagents or intermediates were carried out under argon atmosphere using standard *Schlenk* techniques. All glasswares were dried by the use of a heat gun under high vacuum prior to use. Concentration of the reaction mixture was performed under reduced pressure at 40 °C at the appropriate pressure. Purified compounds were further dried under high vacuum. **All reagents** were purchased of the following companies and have been used without further purification: *Acros Organics*, *Sigma-Aldrich*, *Alfa Aesar*, *TCI Germany* or *Merck*.

2 Experimental procedures

2.1 General procedures

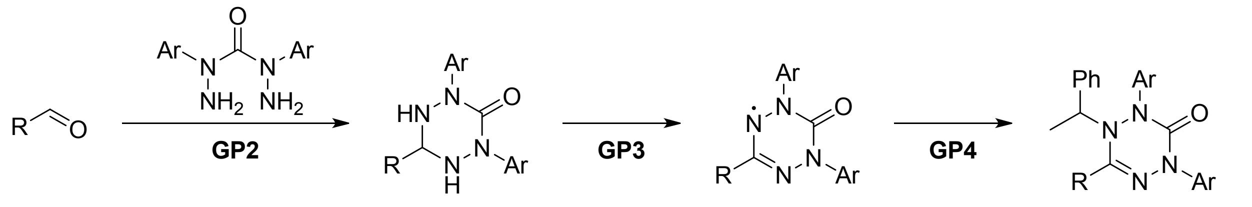

Scheme 1 Synthesis of fluorescent tetrazin-3-one derivatives.

GP1: **Synthesis of aryl substituted carbonohydrazides**

In accordance to a procedure reported by *Masuda et al.*[2] carbohydrazide (1.0 eq.), iodo substituted arene (2.2 eq.), CuI (5 mol-%), 1,10-phenanthroline (10 mol-%) and K_3PO_4 (2.6 eq.) were added to a sealed tube and DMF was added. The mixture was stirred at 90 °C for 20 h. After cooling down to RT, EtOAc was added to the mixture, which was then filtered over Celite. The filtrate was washed with H_2O and the combined aqueous layers were extracted with EtOAc. The combined organic layers were dried over $MgSO_4$ and the solvent was removed *in vacuo*. FC afforded the aryl substituted carbonohydrazide as a solid.

GP2: Synthesis of tetrazinan-3-ones (1 –7)

In accordance to a literature procedure reported by *Neugebauer et al*.[3] a solution of aldehyde (1.0 eq.) in EtOH or DCM was added dropwise to a solution of carbonohydrazide (1.0 eq.) in EtOH at 80 °C. The reaction mixture was refluxed for 6 h and after cooling down to RT the solvent was removed *in vacuo*. The crude product was suspended in MeOH and filtered. After washing with MeOH (three times) the tetrazinan-3-one was obtained as a colorless solid.

GP3: Synthesis of verdazyl radicals (8 – 15)

In a sealed tube 1,4-benzoquinone (1.5 eq. – excess) and tetrazin-3-one (1.0 eq.) were dissolved in DCM and the solution was stirred at 60 °C for 3 h. The solvent was removed *in vacuo* and FC afforded the appropriate verdazyl radical.

GP4: Synthesis of fluorescent tetrazin-3-one derivatives (16 – 23)

In accordance to a literature procedure reported by *Matyjaszewski et al.*[4] a mixture of 1-bromoethyl benzene (1.1 eq.), verdazyl radical (1.0 eq.), Cu (1.1 eq.), $Cu(OTf)_2$ (1 mol-%), 4,4‘-di-*tert*-butyl-2,2‘-bipyridine (2 mol-%) in benzene was stirred at 80 °C for 16 - 24 h. The reaction mixture was filtered over celite and was then extracted with DCM. The solvent of the filtrate was removed *in vacuo* and FC afforded the product as a solid.

2.2 Synthesis of carbonohydrazides

***N,N'*-Diphenylcarbonohydrazide (S1)**

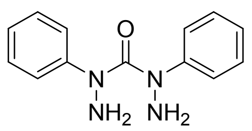

$C_{13}H_{14}N_4O$
242.28 g/mol

In accordance to **GP1** with carbohydrazide (3.00 g, 33.3 mmol, 1.0 eq.), iodobenzene (6.50 mL, 73.7 mmol, 2.2 eq.), CuI (186 mg, 1.7 mmol, 5 mol-%), 1,10-phenanthroline (0.60 g, 3.3 mmol, 10 mol-%) and K_3PO_4 (19.00 g, 86.6 mmol, 2.6 eq.) in DMF (40 mL) at 90 °C for 20 h. FC (MTBE → MTBE/EtOAc, 10:1) afforded *N,N'*-diphenylcarbonohydrazide (3.00 g, 12.4 mmol, 37%) as an orange solid.

^{1}H NMR (300 MHz, $CDCl_3$): δ = 7.22 – 7.10 (*m*, 8H, CH_{arom}), 7.07 – 7.00 (*t*, 2H, CH_{arom}), 3.64 (*br s*, 4H, NH_2); **^{13}C NMR** (75 MHz, $CDCl_3$): δ = 161.8 (C), 144.7 (2 × C), 128.8 (4 × CH), 125.2 (2 × CH), 123.5 (4 × CH); **HRMS** (ESI): *m/z* = 265.1060 calcd. for $[M+Na]^+$, found: 265.1060.
The obtained spectroscopic data are in accord with those reported in the literature.[2]

***N,N‘*-Di-(4-methoxy)phenylcarbonohydrazide (S2)**

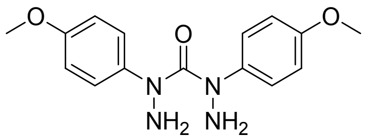

$C_{15}H_{18}N_4O_3$
302.33 g/mol

According to **GP1** with carbohydrazide (3.00 g, 33.3 mmol, 1.0 eq.), iodoanisole (18.00 g, 76.9 mmol, 2.3 eq.), CuI (186 mg, 1.7 mmol, 5 mol-%), 1,10-phenanthroline (0.60 g, 3.3 mmol, 10 mol-%) and K_3PO_4 (19.00 g, 86.6 mmol, 2.6 eq.) in DMF (40 mL) at 90 °C for 20 h. FC (MTBE/EtOAc, 10:1) afforded *N,N‘*-di-(4-methoxy)phenylcarbonohydrazide (2.34 g, 7.7 mmol, 23%) as a colorless solid.

M.p.: 133 °C; **IR** (neat): 3348*w*, 3231*w*, 2957*w*, 2840*w*, 1637*m*, 1605*m*, 1582*m*, 1502*s*, 1470*m*, 1442*m*, 1420*m*, 1360*m*, 1297*m*, 1244*br*, 1181*s*, 1131*m*, 1032*s*, 1009m, 936*m*, 914*m*, 828*s*, 762*m*, 723*m*, 699*m*, 642*m*, 609*m*, 522*s*, 507*w*; **^{1}H NMR** (300 MHz, $CDCl_3$): δ = 6.92 (*d*, *J* = 8.9 Hz, 4H, CH_{arom}), 6.66 (*d*, *J* = 8.9 Hz, 4H, CH_{arom}), 4.44 (*br s*, 4H, NH_2), 3.70 (*s*, 6H,

CH_3); **^{13}C NMR** (75 MHz, $CDCl_3$): δ = 162.8 (C), 157.4 (2 × C), 138.2 (2 × C), 126.0 (4 × CH), 114.1 (4 × CH), 55.5 (2 × CH_3); **HRMS** (ESI): *m/z* = 325.1271, 627.2650 calcd. for $[M+Na]^+$, $[2M+Na]^+$, found: 325.1262, 627.2643.

N,N'-Di-(4-octyloxy)phenyl-carbonohydrazide (S3)

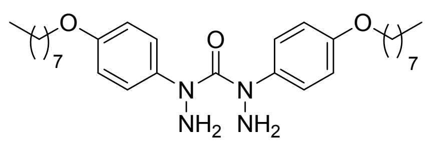

$C_{29}H_{46}N_4O_3$
498.70 g/mol

In accordance to **GP1** with carbohydrazide (3.00 g, 33.3 mmol, 1.0 eq.), 1-iodo-4-octyloxybenzene (24.10 g, 72.5 mol, 2.2 eq.), CuI (0.34 g, 3.3 mmol, 10 mol-%), 1,10-phenanthroline (1.20 g, 6.7 mmol, 20 mol-%) and K_3PO_4 (19.00 g, 86.6 mmol, 2.6 eq.) in DMF (40 mL) at 90 °C for 20 h. After the described extraction procedure and removal of the solvent *in vacuo*, MTBE (50 mL) was added to the residue and the product crystallized over night at - 20 °C. Filtration and washing with cold MTBE afforded *N,N'*-di-(4-octyloxy)phenyl-carbonohydrazide (2.76 g, 5.5 mmol, 17%) as a colorless solid.

IR (neat): 3348*w*, 2957*m*, 2923*s*, 2853*m*, 1637*m*, 1606*m*, 1581*w*, 1504*s*, 1477*s*, 1422*m*, 1366*s*, 1297*s*, 1254*s*, 1177*m*, 1130*w*, 1026*m*, 1000*m*, 936*m*, 914*m*, 872*w*, 830*s*, 737*m*, 721*m*, 702*m*, 645*m*, 616*m*, 549*m*; **^{1}H NMR** (300 MHz, $CDCl_3$): δ = 6.92 (*d*, *J* = 8.9 Hz, 4H, CH_{arom}), 6.68 (*d*, *J* = 8.9 Hz, 4H, CH_{arom}), 4.43 (*br s*, 4H, NH_2), 3.87 (*t*, *J* = 6.6 Hz, 4H, OCH_2), 1.73 (*p*, *J* = 6.6 Hz, 4H, CH_2), 1.35 (*m*, 20H, CH_2), 0.98 – 0.80 (*t*, *J* = 6.7 Hz, 6H, CH_3); **^{13}C NMR** (75 MHz, $CDCl_3$): δ = 157.1 (C), 138.2 (2 × C), 138.1 (2 × C), 126.1 (4 × CH), 114.8 (4 × CH), 68.4 (2 × CH_2), 31.9 (2 × CH_2), 29.4 (2 × CH_2), 29.3 (2 × CH_2), 29.3 (2 × CH_2), 26.1 (2 × CH_2), 22.7 (2 × CH_2), 14.2 (2 × CH_3); **HRMS** (ESI): *m/z* = 499.3643, 521.3462, 1019.7032 calcd. for $[M+H]^+$, $[M+Na]^+$, $[2M+Na]^+$, found: 499.3635, 539.5616, 1019.7001.

N,N'-Dimethylcarbonohydrazide (S4)

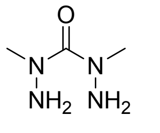

$C_3H_{10}N_4O$
118.14 g/mol

In a modified version to a procedure reported by *Yang et al.*[5] in a two-necked flask with addition funnel and gas inlet, methyl hydrazine (0.86 mL, 16.52 mmol, 11.8 eq.) was dissolved in DCM (50 mL). A solution of triphosgene (415 mg, 1.40 mmol, 1.0 eq.) in DCM (25 mL) was added dropwise at - 78 °C within 1 h. The mixture was allowed to warm to RT and was then stirred overnight. The formed colorless precipitate was filtered off, washed with DCM (2 × 10 mL) and the filtrate concentrated *in vacuo* to afford *N,N'*-dimethylcarbonohydrazide (160 mg, 1.36 mmol, 97%) as a colorless hygroscopic solid.

^{1}H NMR (300 MHz, $CDCl_3$): δ = 4.12 (*br s*, 4H, NH_2), 2.95 (*s*, 6H, CH_3); **HRMS** (ESI): *m/z* = 119.0927, 141.0747 calcd. for $[M+H]^+$, $[M+Na]^+$, found: 119.0911, 141.0737.
The obtained spectroscopic data are in accord with those reported in the literature.[5]

2.3 Synthesis of precursor compounds

Benzene-1,3,5-triyltrimethanol (S5)

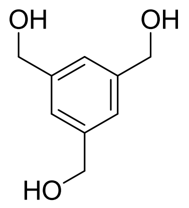

$C_9H_{12}O_3$
168.19 g/mol

A solution of trimethyl 1,3,5-benzenetricarboxylate (8.00 g, 31.72 mmol, 1.0 eq.) in THF (25 mL) was added dropwise to an ice cooled suspension of $LiAlH_4$ (5.55 g, 146.25 mmol, 4.6 eq.) in THF (20 mL) and stirred at RT for 16 h. Then MeOH was added dropwise at 0 °C and the mixture was neutralized with H_2SO_4 (conc.). The precipitate was filtered off, washed with MeOH (50 mL) and the solvent removed *in vacuo*. EtOAc (100 mL) was added to the crude product and the solution was refluxed for 2 h. The suspension was filtered hot to afford benzene-1,3,5-triyltrimethanol (4.76 g, 28.32 mmol, 89%) as a colorless solid

^{1}H NMR (300 MHz, DMSO-d_6): δ = 7.09 (*s*, 3H, CH_{arom}), 5.11 (*s*, 3H, OH), 4.44 (*d*, *J* = 5.5 Hz, 6H, CH_2); **HRMS** (ESI): *m/z* = 191.0679 calcd. for $[M+Na]^+$, found: 191.0681.
The obtained spectroscopic data are in accord with those reported in the literature.[6]

Benzene-1,3,5-tricarbaldehyde (S6)

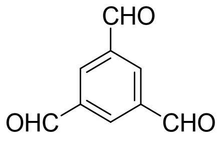

$C_9H_6O_3$
162.14 g/mol

In a round bottom flask 4-hydroxy-2,2,6,6-tetramethylpiperidin-1-oxyl (0.10 g, 0.58 mmol, 9 mol-%), **S5** (1.11 g, 6.60 mmol, 1.0 eq.) and *tert*-butylnitrite (1 mL, 0.58 mmol, 9 mol-%) were dissolved in AcOH (20 mL). The reaction mixture was stirred for 3 d at RT under an O_2-atmosphere. The precipitate was filtered off to afford benzene-1,3,5-tricarbaldehyde (0.54 g, 3.33 mmol, 50%) as a colorless solid.

^{1}H NMR (300 MHz, $CDCl_3$): δ = 10.20 (*s*, 3H, C*H*O), 8.63 (*s*, 3H, CH_{arom}); **^{13}C NMR** (75 MHz, $CDCl_3$): δ = 189.8 (3 × C), 137.8 (3 × CH), 134.7 (3 × CH).
The obtained spectroscopic data are in accord with those reported in the literature.[6]

2.4 Synthesis of tetrazinan-3-ones

2,4,6-Triphenyl-1,2,4,5-tetrazinan-3-one (1)

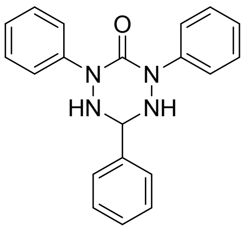

$C_{20}H_{18}N_4O$
330.38 g/mol

According to **GP2** with benzaldehyde (0.10 g, 0.99 mmol, 1.0 eq.) and **S1** (0.24 g, 0.99 mmol, 1.0 eq.). Filtration afforded **1** (0.30 g, 0.91 mmol, 91%) as a colorless solid.

M.p.: 191 °C; **IR** (neat): 3229*m*, 3059*w*, 1621*s*, 1489*s*, 1377*s*, 1037*s*, 1225*m*, 1112*w*, 1075*w*, 1028*m*, 926*s*, 849*m*, 744*s*, 691*s*, 626*m*, 510*m*; **^{1}H NMR** (300 MHz, DMSO-d_6): δ = 7.60 (d, J = 7.7 Hz, 4H, CH_{arom}), 7.54 (d, J = 6.8 Hz, 2H, CH_{arom}), 7.41 – 7.26 (m, 7H, CH_{arom}), 7.07 (t, J = 7.2 Hz, 2H, CH_{arom}), 6.38 (d, J = 9.1 Hz, 2H, N*H*), 5.38 (t, J = 9.0 Hz, 1H, NC*H*N); **^{13}C NMR** (75 MHz, DMSO-d_6): δ = 156.8 (C), 142.8 (2 × C), 137.7 (C), 128.3 (2 × CH), 128.1 (CH), 128.0 (4 × CH), 126.9 (2 × CH), 123.3 (2 × CH), 121.2 (4 × CH), 72.8 (CH); **HRMS** (ESI): *m/z* = 353.1373, 683.2853, 1013.4334 calcd. for $[M+Na]^+$, $[2M+Na]^+$, $[3M+Na]^+$, found: 353.1368, 683.2843, 1013.4315; **Anal. Calcd.** for $C_{20}H_{18}N_4O$, C: 72.71, H: 5.49, N: 16.96; found: 72.59, 5.49, 16.80.

2,4-Bis(4-methoxyphenyl)-6-phenyl-1,2,4,5-tetrazinan-3-one (1*)

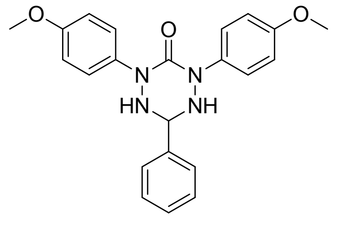

$C_{22}H_{22}N_4O_3$
390.44 g/mol

According to **GP2** with benzaldehyde (0.22 mL, 1.96 mmol, 0.9 eq.) and **S2** (0.66 g, 2.18 mmol, 1.0 eq.). Filtration afforded **1*** (0.48 g, 1.22 mmol, 56%) as a colorless solid.

M.p.: 183 °C; **IR** (neat): 3231*m*, 2938*w*, 2912*w*, 2840*w*, 1622*m*, 1602*m*, 1584*m*, 1507*s*, 1466*m*, 1441*m*, 1424*w*, 1368*s*, 1340*m*, 1323*m*, 1297*m*, 1244*s*, 1168*m*, 1129*w*, 1106*w*, 1031*m*, 1010*m*, 916*m*, 883*w*, 830*s*, 795*m*, 752*m*, 724*m*, 698*s*, 627*m*, 570*m*, 546*m*, 522*s*; **^{1}H NMR** (300 MHz, DMSO-d_6): δ = 7.55 (*d*, J = 6.3 Hz, 2H, CH_{arom}), 7.45 (*d*, J = 9.0 Hz, 4H, CH_{arom}), 7.41 – 7.32 (*m*, 3H, CH_{arom}), 6.89 (*d*, J = 9.0 Hz, 4H, CH_{arom}), 6.29 (*d*, J = 9.2 Hz, 2H, NH), 5.34 (*t*, J = 9.1 Hz, 1H, NC*H*N), 3.74 (*s*, 6H, CH_3); **^{13}C NMR** (75 MHz, DMSO-d_6): δ = 155.8 (2 × C), 154.5 (C), 137.0 (2 × C), 136.3 (CH), 128.2 (2 × CH), 128.1 (C), 126.9 (2 × CH), 124.0 (4 × CH), 113.2 (4 × CH), 71.6 (CH), 55.2 (2 × CH_3); **HRMS** (ESI): *m/z* = 391.1770, 413.1590, 803.3282, 1193.4873 calcd. for $[M+H]^+$, $[M+Na]^+$, $[2M+Na]^+$, $[3M+Na]^+$, found: 391.1764, 413.1579, 803.3264, 1193.4897.

6-Mesityl-2,4-diphenyl-1,2,4,5-tetrazinan-3-one (2)

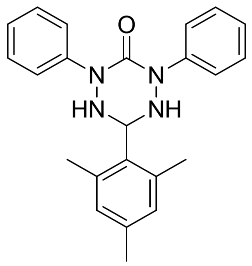

$C_{23}H_{24}N_4O$
372.46 g/mol

According to **GP2** with mesitylcarbaldehyde (0.66 g, 4.47 mmol, 1.0 eq.) and **S1** (1.08 g, 4.47 mmol, 1.0 eq.). Filtration afforded **2** (0.54 g, 1.45 mmol, 32%) as a colorless solid.

M.p.: 161 °C; **IR** (neat): 3342*w*, 3064*w*, 2926*m*, 1692*m*, 1651*m*, 1636*m*, 1597*m*, 1493*m*, 1454*m*, 1384*s*, 1308*s*, 1159*m*, 1123*m*, 1073*m*, 1027*m*, 968*w*, 900*m*, 858*m*, 752*s*, 730*m*, 689*m*, 666*m*, 618*m*, 575*m*, 535*m*, 510*m*; **^{1}H NMR** (300 MHz, $CDCl_3$): δ = 7.66 (*d*, J = 7.7 Hz, 4H, CH_{arom}), 7.38 – 7.30 (*m*, 4H, CH_{arom}), 7.12 (*t*, J = 7.4 Hz, 2H, CH_{arom}), 6.90 (*s*, 2H, CH_{arom}), 5.73 (*t*, 1H, J = 9.1 Hz, NC*H*N), 5.00 (*d*, J = 11.1 Hz, 2H, NH), 2.45 (*s*, 6H, CH_3), 2.29 (*s*, 3H, CH_3); **^{13}C NMR** (75 MHz, $CDCl_3$): δ = 156.1 (C), 141.9 (2 × C), 138.7 (C), 136.6 (2 × C), 130.7 (2 × CH), 129.3 (C), 128.5 (4 × CH), 124.5 (2 × CH), 121.8 (4 × CH), 72.6 (CH), 21.3 (2 × CH), 20.9 (CH); **HRMS** (ESI): *m/z* = 395.1842 calcd. for $[M+Na]^+$, found: 395.1844.

6-(Naphthalen-1-yl)-2,4-diphenyl-1,2,4,5-tetrazinan-3-one (3)

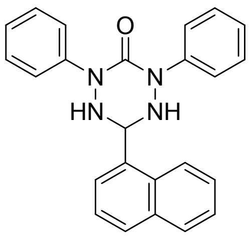

$C_{24}H_{20}N_4O$
380.44 g/mol

According to **GP2** with 1-naphthaldehyde (0.94 g, 7.58 mmol, 1.0 eq.) and **S1** (1.83 g, 7.58 mmol, 1.0 eq.). Filtration afforded **3** (1.81 g, 4.76 mmol, 63%) as a colorless solid.

M.p.: 217 °C; **IR** (neat): 3240*s*, 3061*br*, 2911*w*, 1669*s*, 1595*s*, 1496*s*, 1454*m*, 1337*s*, 1312*s*, 1285*m*, 1226*m*, 1156*m*, 1106*m*, 951*m*, 905*m*, 803*m*, 780*m*, 750*s*, 738*s*, 727*m*, 687*s*, 632*s*, 590*s*, 568*s*, 553*s*, 522*s*; **^{1}H NMR** (300 MHz, DMSO-d_6): δ = 8.42 (*d*, J = 8.5 Hz, 1H, CH_{arom}), 7.96 (*d*, J = 8.5 Hz, 1H, CH_{arom}), 7.90 (*d*, J = 8.2 Hz, 1H, CH_{arom}), 7.65 (*m*, 6H, CH_{arom}), 7.57 (*t*, J = 7.4 Hz, 1H, CH_{arom}), 7.46 - 7.31 (*m*, 5H, CH_{arom}), 7.09 (*t*, J = 7.4 Hz, 2H, CH_{arom}), 6.53 (*d*, J = 9.3 Hz, 2H, N*H*), 6.06 (*t*, J = 9.3 Hz, 1H, NC*H*N); **^{13}C NMR** (75 MHz, DMSO-d_6): δ = 159.3 (C), 142.6 (2 × C), 133.6 (C), 133.2 (C), 130.7 (C), 128.6 (CH), 128.3 (CH), 128.1 (4 × CH), 126.3 (CH), 125.7 (CH), 125.2 (CH), 124.3 (CH), 124.2 (CH), 123.0 (2 × CH), 120.5 (4 × CH), 71.5 (CH); **HRMS** (ESI): *m/z* = 381.1710, 403.1529 calcd. for $[M+H]^+$, $[M+Na]^+$, found: 381.1710, 403.1535.

2,4-Diphenyl-6-(pyren-1-yl)-1,2,4,5-tetrazinan-3-one (4)

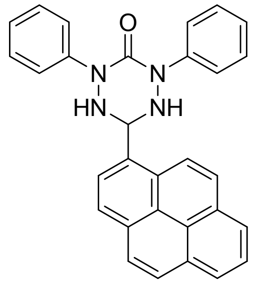

According to **GP2** with 1-pyrenecarboxaldehyde (0.34 g, 1.49 mmol, 1.0 eq.) and **S1** (0.40 g, 1.67 mmol, 1.1 eq.). Filtration afforded **4** (0.46 g, 1.02 mmol, 69%) as a colorless solid.

M.p.: 227 °C; **IR** (neat): 3255*w*, 3035*w*, 1618*m*, 1485*m*, 1382*m*, 1309*m*, 1090*s*, 929*m*, 846*m*, 754*m*, 684*m*, 646*s*; **^{1}H NMR** (600 MHz, DMSO-d_6): δ = 8.68 (*d*, *J* = 9.3 Hz, 1H, CH_{arom}), 8.37 – 8.28 (*m*, 3H, CH_{arom}), 8.23 – 8.17 (*m*, 3H, CH_{arom}), 8.15 – 8.08 (*m*, 2H, CH_{arom}), 7.72 (*d*, *J* = 7.7 Hz, 4H, CH_{arom}), 7.42 – 7.34 (*m*, 4H, CH_{arom}), 7.11 (*t*, *J* = 7.4 Hz, 2H), 6.62 (*d*, *J* = 9.3 Hz, 2H, N*H*), 6.39 (*t*, *J* = 9.3 Hz, 1H, NC*H*N); **^{13}C NMR** (151 MHz, DMSO-d_6): δ = 157.5 (C), 142.5 (2 × C), 130.6 (C), 130.4 (C), 130.4 (C), 129.8 (C), 127.9 (C), 127.5 (8 × CH), 127.1 (CH), 127.0 (CH), 126.7 (CH), 125.7 (CH), 124.9 (CH), 124.8 (CH), 124.1 (CH), 123.8 (CH), 123.6 (C), 123.5 (C), 123.1 (CH), 122.8 (CH), 120.7 (CH), 70.9 (CH); **HRMS** (ESI): *m/z* = 455.1866, 477.1686 calcd. for $[M+H]^+$, $[M+Na]^+$; found: 455.1864, 477.1685.

6,6'-(1,4-Phenylene)bis(2,4-diphenyl-1,2,4,5-tetrazinan-3-one) (5)

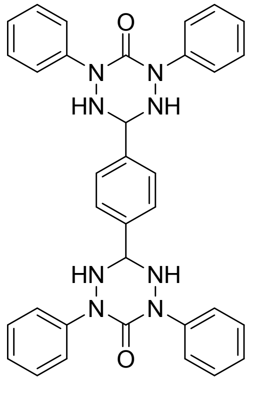

According to **GP2** with terephthalaldehyde (0.23 g, 1.72 mmol, 1.0 eq.) and **S1** (0.85 g, 3.51 mmol, 2.0 eq.). Filtration afforded **5** (0.69 g, 1.18 mmol, 69%) as a colorless solid.

M.p.: 215 °C; **IR** (neat): 3228*w*, 3065*w*, 1669*w*, 1624*m*, 1594*m*, 1490*m*, 1379*s*, 1310*s*, 1223*s*, 1206*s*, 923*s*, 815*w*, 738*s*, 690*s*, 664*s*, 569*m;* **^{1}H NMR** (300 MHz, DMSO-d_6): δ = 7.60 (*d*, *J* = 8.2 Hz, 8H, CH_{arom}), 7.52 (*s*, 4H, CH_{arom}), 7.33 (*t*, *J* = 7.8 Hz, 8H, CH_{arom}), 7.07 (*t*, *J* = 7.3 Hz, 4H, CH_{arom}), 6.38 (*d*, *J* = 8.9 Hz, 4H, NH), 5.38 (*t*, *J* = 8.9 Hz, 2H, NC*H*N). **^{13}C NMR** (75 MHz, DMSO-d_6): δ = 156.8 (2 × C), 142.7 (4 × C), 137.7 (2 × CH), 128.0 (8 × CH), 126.9 (4 × CH), 123.2 (4 × CH), 121.1 (8 × CH), 72.6 (2 × CH); **HRMS** (ESI): *m/z* = 605.2384, 1187.4867 calcd. for $[M+Na]^+$, $[2M+Na]^+$, found: 605.2375, 1187.4803.

6,6'-(1,3-Phenylene)bis(2,4-diphenyl-1,2,4,5-tetrazinan-3-one) (6)

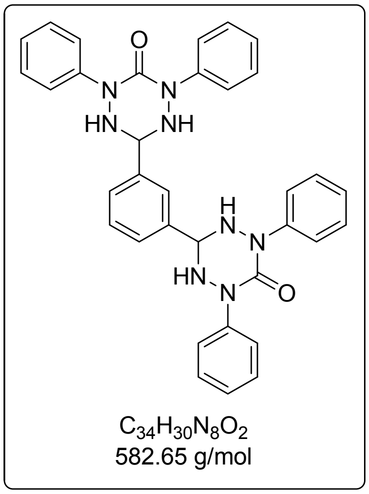

According to **GP2** with isophthalaldehyde (0.55 g, 4.13 mmol, 1.0 eq.) and **S1** (2.00 g, 8.26 mmol, 2.0 eq.). Filtration afforded **6** (3.87 g, 6.65 mmol, 81%) as a colorless solid.

M.p.: 216 °C; **IR** (neat): 3251*w*, 3064*w*, 1685*w*, 1633*m*, 1595*m*, 1487*s*, 1431s, 1384*s*, 1307*m*, 1217*m*, 1163*m*, 1129*w*, 1074*w*, 988*w*, 890*m*, 754 *s*, 717*m*, 690*s*, 607*m*; **^{1}H NMR** (300 MHz, DMSO-d_6): δ = 7.80 (*s*, 1H, CH_{arom}), 7.60 (*d*, *J* = 8.0 Hz, 8H, CH_{arom}), 7.50 (*d*, *J* = 7.5 Hz, 2H, CH_{arom}), 7.35 – 7.32 (*m,* 9H, CH_{arom}), 7.07 (*t*, *J* = 7.3 Hz, 4H, CH_{arom}), 6.35 (*d*, *J* = 9.0 Hz, 4H, NH), 5.34 (*t*, *J* = 8.9 Hz, 2H, NC*H*N); **^{13}C NMR** (75 MHz, DMSO-d_6): δ = 156.8 (2 × C), 142.7 (4 × C), 137.7 (2 × C), 128.0 (8 × CH), 126.9 (4 × CH), 123.3 (CH), 123.2 (2 × CH), 121.2 (CH), 121.1 (8 × CH), 72.6 (2 × CH); **HRMS** (ESI): *m/z* = 605.2384, 1187.4867 calcd. for $[M+Na]^+$, $[2M+Na]^+$, found: 605.2384, 1187.5121.

6,6',6''-(Benzene-1,3,5-triyl)tris(2,4-diphenyl-1,2,4,5-tetrazinan-3-one) (7)

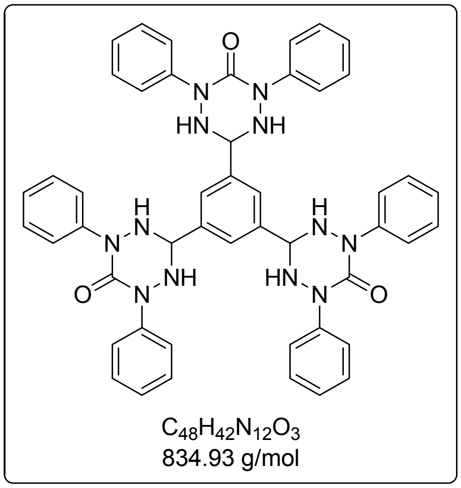

According to **GP2** with 1,3,5-triformylbenzene (0.23 g, 1.42 mmol, 0.33 eq.) and **S1** (1.05 g, 4.34 mmol, 3.1 eq.). Filtration afforded **7** (0.57 g, 0.69 mmol, 48%) as a colorless solid.

M.p.: 229 °C; **IR** (neat): 3239*w*, 3064*w*, 1695*s*, 1641*s*, 1595*m*, 1488*m*, 1458*m*, 1374*s*, 1310*m*, 1262*w*, 1222*m*, 1175*w*, 1133*w*, 1072*w*, 1028*w*, 900w, 876m, 746s, 712*m*, 678*s*, 619*s*, 611*s*, 512*s;* **^{1}H NMR** (300 MHz, DMSO-d_6): δ = 7.82 (*s*, 3H, CH_{arom}), 7.58 (*d*, *J* = 7.9 Hz, 12H, CH_{arom}), 7.30 (*t*, *J* = 7.8 Hz, 12H, CH_{arom}), 7.07 (*t*, *J* = 7.3 Hz, 6H, CH_{arom}), 6.26 (*d*, *J* = 9.1 Hz, 6H, NH), 5.29 (*t*, *J* = 9.1 Hz, 3H, NC*H*N); **^{13}C NMR** (75 MHz, DMSO-d_6): δ = 155.8 (3 × C), 142.7 (6 × C), 137.6 (3 × C), 127.9 (12 × CH), 125.6 (3 × CH), 123.3 (6 × CH), 121.4 (12 × CH), 72.3 (3 × CH); **HRMS** (ESI): *m/z* = 857.3395 calcd. for $[M+Na]^+$, found: 857.3369.

2.5 Synthesis of verdazyl radicals

1,3,5-Triphenyl-6-oxo-verdazyl radical (8)

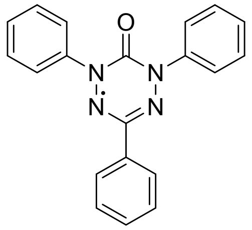

$C_{20}H_{15}N_4O^{\bullet}$
327.36 g/mol

N,*N'*-Diphenylcarbonohydrazide (**S1**) (0.80 g, 3.29 mmol, 1.0 eq.) was dissolved in EtOH (100 mL) and a solution of benzaldehyde (0.31 mL, 3.29 mmol, 1.0 eq.) in EtOH (20 mL) was added dropwise at 80 °C. The mixture was refluxed for 4 h and then 1,4-benzoquinone (0.53 g, 4.94 mmol, 1.5 eq.) was added to the suspension, which was then stirred at 80 °C for 3 h. After removal of the solvent *in vacuo* the residue was purified by FC (pentane/DCM, 5:1) to afford verdazyl radical **8** (0.97 g, 2.93 mmol, 89%) as a dark red solid.

HRMS (ESI): *m/z* = 329.1397, 350.1138 calcd. for $[M+H]^+$, $[M+Na]^+$, found: 329.1398, 350.1145. The obtained spectroscopic data are in accord with those reported in the literature.[7]

1,5-Di-(4-methoxy)phenyl-3-phenyl-6-oxo-verdazyl (8*)

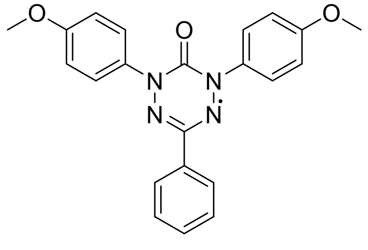

$C_{22}H_{19}N_4O_3^{\bullet}$
387.41 g/mol

According to **GP3** with 1,4-benzoquinone (0.30 g, 2.78 mmol, 3.0 eq.) and **1*** (0.36 g, 0.93 mmol, 1.0 eq.). FC (pentane/DCM, 5:1) afforded **8*** (0.30 g, 0.77 mmol, 82%) as a dark red solid.

M.p.: 199 °C; **IR** (neat): 3059*m*, 2981*w*, 2836*w*, 1685*s*, 1615*w*, 1503*s*, 151*m*, 1443*m*, 1342*s*, 1297*s*, 1239*s*, 1168*m*, 1110*m*, 1096*m*, 1029*s*, 918*w*, 832*s*, 794*w*, 750*s*, 703*s*, 689*s*, 655*w*, 608*w*, 577*w*, 553*w*, 519*m*; **HRMS** (ESI): *m/z* = 410.1349, 797.2807, 1184.4269 calcd. for $[M+Na]^+$, $[2M+Na]^+$, $[3M+Na]^+$, found: 410.1347, 797.2796, 1184.4191.
The obtained spectroscopic data are in accord with those reported in the literature.[8]

1,5-Diphenyl-3-mesityl-6-oxo-verdazyl radical (9)

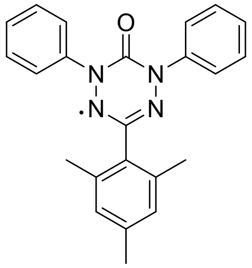

$C_{23}H_{21}N_4O^{\bullet}$
369.44 g/mol

According to **GP3** with **2** (400 mg, 1.07 mmol, 1.0 eq.) and 1,4-benzoquinone (340 mg, 3.18 mmol, 3.0 eq.). FC (pentane/DCM, 5:1) afforded verdazyl radical **9** (0.20 g, 0.54 mmol, 51%) as a dark red solid.

M.p.: 172 °C; **IR** (neat): 2921*m*, 1695*s* 1590*m*, 1485*s*, 1442*m*, 1359*m*, 1315*w*, 1236*m*, 1171*m*, 1111*m*, 1031*m*, 911*w*, 854*m*, 745*s*, 688*s*; **HRMS** (ESI): *m/z* = 392.1608 calcd. for $[M+Na]^+$, found: 392.1597.

1,5-Diphenyl-3-(1'-naphthyl)-6-oxo-verdazyl radical (10)

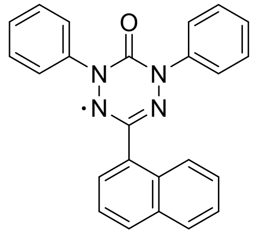
$C_{24}H_{17}N_4O^{\bullet}$
377.42 g/mol

According to **GP3** with **3** (1.48 g, 3.89 mmol, 1.0 eq.) and 1,4-benzoquinone (0.65 g, 6.13 mmol, 1.6 eq.). FC (pentane/DCM, 5:1) afforded verdazyl radical **10** (1.44 g, 3.81 mmol, 98%) as a dark red solid.

M.p.: 166 °C; **IR** (neat): 3055*w*, 2956*w*, 2922*w*, 2855*w*, 1734*w*, 1697*s*, 1630*w*, 1589*w*, 1510*w*, 1480*m*, 1459*m*, 1422*w*, 1364*m*, 1340*w*, 1294*w*, 1261*w*, 1237*m*, 1215*m*, 119*m*, 1176*m*, 1139*m*, 1085*w*, 1069*w*, 1029*w*, 904*w*, 896*w*, 799*s*, 772*s*, 753*s*, 731*s*, 680*s*, 611*s*, 600*s*, 567*w*, 545*s*, 502*s*; **HRMS** (ESI): *m/z* = 378.1475, 400.1295 calcd. for $[M+H]^+$, $[M+Na]^+$, found: 378.1465, 400.1286; **Anal. Calcd.** for $C_{24}H_{17}N_4O$,· C: 76.38, H: 4.54, N: 14.81; found: 76.30, 4.56, 14.91.

1,5-Diphenyl-3-(9'-anthracenyl)-6-oxo-verdazyl radical (11)

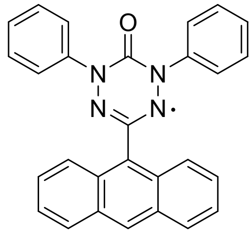
$C_{28}H_{19}N_4O^{\bullet}$
427.48 g/mol

To a solution of 2,4-diphenylcarbonohydrazide (1.00 g, 4.13 mmol, 1.0 eq.) in EtOH (100 mL) a solution of 9-anthracenecarboxaldehyde (0.85 g, 4.14 mmol, 1.0 eq.) in EtOH (10 mL) was added dropwise at 80 °C. After addition the reaction mixture was refluxed for 4 h and then 1,4-benzoquinone (0.26 g, 2.53 mmol, 1.5 eq.) was added. The reaction mixture was stirred at 80 °C for 3 h. The solvents were removed *in vacuo* and FC (pentane/DCM, 10:1) afforded verdazyl radical **11** (0.22 g, 0.51 mmol, 30%) as a red solid.

M.p.: 226 °C; **IR** (neat): 3057*w*, 2963*w*, 1697*s*, 1591*m*, 1522*w*, 1486*s*, 1459*m*, 1396*w*, 1362*w*, 1253*s*, 1200*s*, 1088*s*, 1069*s*, 1014s, 968*w*, 890*s*, 843*m*, 788*m*, 762*s*, 729*s*, 720*s*, 689*s*, 614*w*, 602*s*, 584*s*, 562*w*, 539*m*, 516*s*; **HRMS** (ESI): *m/z* = 450.1451 calcd. for $C_{28}H_{19}N_4O$ $[M+Na]^+$, found: 450.1455.

1,5-Diphenyl-3-(1'-pyrenyl)-6-oxo-verdazyl radical (12)

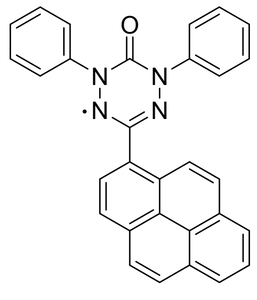

$C_{30}H_{19}N_4O^{\bullet}$
451.50 g/mol

According to **GP3** with **4** (0.28 g, 0.62 mmol, 1.0 eq.) and 1,4-benzoquinone (0.11 g, 0.99 mmol, 1.6 eq.). FC (pentane/DCM, 5:1) afforded verdazyl radical **12** (0.27 g, 0.59 mmol, 96%) as a dark red solid.

M.p.: 186 °C; **IR** (neat): 3368*w*, 3042*w*, 2982*w*, 1942*w*, 1776*w*, 1734*m*, 1690*s*, 1592*m*, 1511*w*, 1484*m*, 1457*m*, 1400*m*, 1372*m*, 1351*m*, 1311*w*, 1296*w*, 1232*s*, 1176, 1147*s*, 1136*m*, 1097*m*, 1069*m*, 1045*m*, 971*w*, 901*m*, 849*s*, 824*m*, 796*w*, 746*m*, 729*s*, 679*s*, 651*s*, 617*s*, 604*m*, 560*m*, 529*m*, 506*m*; **HRMS** (ESI): *m/z* = 452.1632, 474.1451 calcd. for $[M+H]^+$, $[M+Na]^+$, found: 452.1613, 474.1448.

1,4-Bis(1,5-diphenyl-6-oxo-3-verdazyl)benzene radical (13)

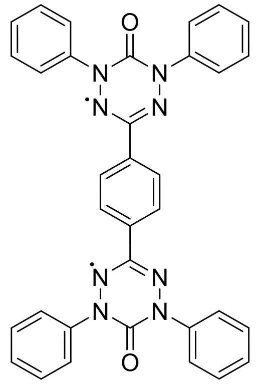

$C_{34}H_{24}N_8O_2^{2\bullet}$
576.61 g/mol

According to **GP3** with **5** (0.33 g, 0.57 mmol, 1.0 eq.) and 1,4-benzoquinone (0.50 g, 4.71 mmol, 8.3 eq.) in DCM (15 mL) at 120 °C for 16 h. Filtration afforded verdazyl radical **13** (0.29 g, 0.50 mmol, 88%) as a green solid.

M.p.: >260 °C; **IR** (neat): 3066*w*, 1744*w*, 1693*s*, 1593*w*, 1486*m*, 1458*m*, 1398*w*, 1347*m*, 1313*w*, 1249*m*, 1177*w*, 1127*s*, 1103*w*, 1074*w*, 1028*w*, 894*w*, 848*m*, 822*w*, 759*w*, 738*s*, 680*s*, 658*s*, 650*s*, 618*w*, 604*s*, 533*w*; **MS** (EI): *m/z* = 576.19 calcd. for $[M+Na]^+$, found: 576.19; **Anal. Calcd.** for $C_{34}H_{24}N_8O_2$, C: 70.82, H: 4.20, N: 19.43; found: 70.01, 4.15, 19.07

1,3-Bis(1,5-diphenyl-6-oxo-3-verdazyl)benzene radical (14)

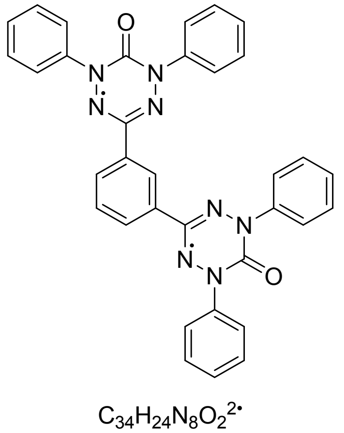

$C_{34}H_{24}N_8O_2^{2\bullet}$
576.61 g/mol

In a sealed tube **6** (0.60 g, 1.03 mmol, 1.0 eq.) and $NaIO_4$ (0.50 g, 2.35 mmol, 2.3 eq.) were dissolved in a mixture of DCM (20 mL) and H_2O (5 mL) and the suspension was stirred at 120 °C for 16 h. Filtration afforded verdazyl radical **14** (0.39 g, 0.68 mmol, 66%) as a purple solid.

M.p.: >260 °C; **IR** (neat): 3182*w*, 3067*w*, 1675*s*, 1596*m*, 1486*m*, 1457*m*, 1409*w*, 1357*m*, 1335*s*, 1225*m*, 1159*m*, 1078*w*, 1028*w*, 964*w*, 808*w*, 749*s*, 727*s*, 715*m*, 687*s*, 617*s*, 571*m*, 515*s*;

HRMS (ESI): *m/z* = 599.1914 calcd. for $[M+Na]^+$, found: 599.1919; **Anal. Calcd.** for $C_{34}H_{24}N_8O_2$ C: 70.82, H: 4.20, N: 19.4, found: 69.97, 3.83, 19.02.

1,3-Bis(1,5-di-(4-octyloxy)phenyl-6-oxo-3-verdazyl)benzene (14*)

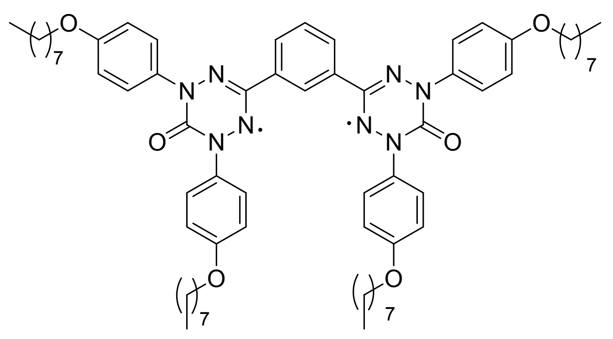

$C_{66}H_{88}N_8O_6{}^{2\bullet}$
1089.4549 g/mol

N,N'-Di-(4-octyloxy)phenylcarbonohydrazide (**S3**) (0.50 g, 1.01 mmol, 2.0 eq.) was dissolved in EtOH (50 mL) and a solution of isophthalaldehyde (0.07 g, 0.50 mmol, 1.0 eq.) in EtOH (20 mL) was added dropwise at 80 °C and stirred for 4 h. After addition of 1,4-benzoquinone (0.49 g, 4.52 mmol, 9.0 eq.) the mixture was stirred at 80 °C for 3 h. Removal of the solvent *in vacuo* and FC (pentane/DCM, 4:1) afforded **14*** (0.29 g, 0.27 mmol, 60%) as a purple solid.

M.p.: 76 °C; **IR** (neat): 2914*s*, 2851*s*, 1682*s*, 1606*s*, 1505*s*, 1470*m*, 1390*m*, 1328*m*, 1299*m*, 1242*s*, 1168*m*, 1129*w*, 1046*m*, 932*w*, 826*m*, 754*w*, 693*m*, 644*m*, 597*m*; **MS** (MALDI): *m/z* = 1089.69 calcd. for $[M+H]^+$, found: 1088.68.

1,3,5-Tris(1,5-diphenyl-6-oxo-3-verdazyl)benzene radical (15)

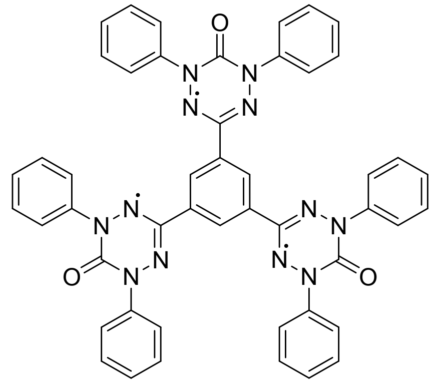

$C_{48}H_{33}N_{12}O_3{}^{3\bullet}$
825.85 g/mol

According to **GP3** with **7** (0.47 g, 0.56 mmol, 1.0 eq.) and 1,4-benzoquinone (0.60 g, 5.66 mmol, 10.1 eq.) in DCM (15 mL) at 120 °C for 16 h. Filtration afforded verdazyl radical **15** (0.46 g, 0.55 mmol, 98%) as a dark purple solid.

M.p.: >260 °C; **IR** (neat): 3067*w*, 1739*w*, 1693*s*, 1593*w*, 1485*m*, 1458*m*, 1394*w*, 1357*m*, 1316*w*, 1260*m*, 1221*s*, 1178*w*, 1066*w*, 1028*w*, 899*w*, 844*w*, 745*s*, 711*m*, 678*s*, 639*m*, 618*s*, 539*m*; **MS** (MALDI): *m/z* = 825.25 calcd. for $[M]^+$, found: 825.25; **Anal. Calcd.** for $C_{48}H_{33}N_{12}O_3$, C: 69.81, H: 4.03, N: 20.35; found: 69.36, 3.92, 19.61.

2.6 Synthesis of fluorescent tetrazin-3-ones

2,4,6-Triphenyl-5-(1-phenylethyl)-4,5-dihydro-1,2,4,5-tetrazin-3(2H)-one (16)

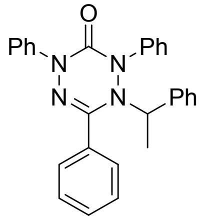

$C_{28}H_{24}N_4O$
432.52 g/mol

According to **GP4** with 1-bromoethylbenzene (155 µL, 1.13 mmol, 1.2 eq.), verdazyl radical **8** (310 mg, 0.95 mmol, 1.0 eq.), Cu (72 mg, 1.13 mmol, 1.2 eq.), $Cu(OTf)_2$ (3.4 mg, 9.4 µmol, 1 mol-%), 4,4'-di-*tert*-butyl-2,2'-bipyridine (5.1 mg, 19.0 µmol, 2 mol-%) in benzene (14 mL) at 80 °C for 20 h. FC (pentane/DCM, 5:1) afforded **16** (290 mg, 0.68 mmol, 72%) as a colorless solid.

M.p.: 140 °C; **IR** (neat): 3064*w*, 2972*w*, 1692*s*, 1595*m*, 1485*m*, 1456*m*, 1366*w*, 1331*s*, 1302*s*, 1208*w*, 1167*m*, 1131*w*, 1095*w*, 1071*w*, 1054*w*, 1028*w*, 995*w*, 905*w*, 836*w*, 779*w*, 758*s*, 736*s*, 691*s*, 628*s*, 602*s*, 573*s*, 540*m*; **^{1}H NMR** (300 MHz, $CDCl_3$): δ = 8.14 – 8.05 (*m*, 2H, CH_{arom}), 7.90 (*d*, *J* = 7.2 Hz, 2H, CH_{arom}), 7.60 – 7.55 (*m*, 3H, CH_{arom}), 7.44 – 7.32 (*m*, 7H, CH_{arom}), 7.27 – 7.07 (*m*, 4H, CH_{arom}), 6.98 (*d*, *J* = 7.3 Hz, 2H, CH_{arom}), 4.63 (*q*, *J* = 7.1 Hz, 1H, CH), 1.52 (*d*, *J* = 7.2 Hz, 3H, CH_3); **^{13}C NMR** (75 MHz, $CDCl_3$): δ = 153.0 (C), 149.9 (C), 144.8 (C), 140.2 (C), 138.8 (C), 131.3 (C), 131.1 (CH), 129.3 (2 × CH), 128.9 (2 × CH), 128.8 (CH), 128.7 (2 × CH), 128.1 (2 × CH), 127.8 (2 × CH), 127.7 (2 × CH), 125.8 (CH), 125.0 (CH), 123.7 (2 × CH), 121.5 (2 × CH), 63.5 (CH), 18.2 (CH_3); **HRMS** (ESI): *m/z* = 433.2023, 455.1842 calcd. for $[M+H]^+$, $[M+Na]^+$, found: 433.2015, 455.1834.

6-Mesityl-2,4-diphenyl-5-(1-phenylethyl)-4,5-dihydro-1,2,4,5-tetrazin-3(2H)-one (17)

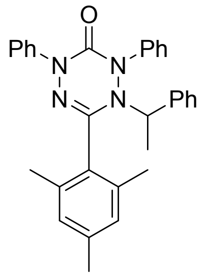

$C_{31}H_{30}N_4O$
474.60 g/mol

According to **GP4** with 1-bromoethylbenzene (90 µL, 0.73 mmol, 1.5 eq.), verdazyl radical **9** (180 mg, 0.49 mmol, 1.0 eq.), Cu (40 mg, 0.59 mmol, 1.2 eq.), $Cu(OTf)_2$ (1.8 mg, 4.9 µmol, 1 mol-%), 4,4'-di-*tert*-butyl-2,2'-bipyridine (2.6 mg, 9.8 µmol, 2 mol-%) in benzene (8 mL) at 80 °C for 20 h. FC (pentane/DCM, 5:1) afforded **17** (0.12 g, 0.25 mmol, 52%) as a colorless solid.

M.p.: 152 °C; **IR** (neat): 3063*w*, 2982*w*, 2926*w*, 1687*s*, 1630*w*, 1611*w*, 1596*m*, 1495*m*, 1450*m*, 1341*s*, 1322*s*, 1307*s*, 1276*m*, 1206*w*, 1160*w*, 1090*w*, 1064*m*, 1029*m*, 1013*m*, 967*w*, 931*w*, 853*m*, 833*w*, 793*w*, 759*s*, 744*s*, 733*s*, 687*s*, 663*m*, 607*m*, 595*s*, 559*w*, 544*m*, 513*s;* **^{1}H NMR** (300 MHz, CD_2Cl_2): δ = 7.58 (*d*, *J* = 7.7 Hz, 2H, CH_{arom}), 7.43 (*t*, *J* = 7.9 Hz, 2H, CH_{arom}), 7.35 – 7.14 (*m*, 10H, CH_{arom}), 7.09 (*t*, *J* = 7.2 Hz, 1H, CH_{arom}), 6.95 (*s*, 2H, CH_{arom}), 4.65 (*q*, *J* = 7.0 Hz, 1H, C*H*CH_3), 2.35 (*s*, 9H, CH_3), 1.77 (*d*, *J* = 7.0 Hz, 3H, CHC*H*$_3$); **^{13}C NMR** (75 MHz, CD_2Cl_2): δ = 154.0 (C), 152.2 (C), 142.9 (C), 141.2 (C),

140.4 (C), 140.3 (C), 139.1 (2 × C), 129.8 (2 × CH), 129.0 (2 × CH), 128.9 (2 × CH), 128.8 (2 × CH), 128.7 (2 × CH), 128.5 (CH), 128.5 (C), 126.3 (CH), 125.8 (CH), 124.0 (2 × CH), 123.8 (2 × CH), 62.4 (CH), 21.4 (CH_3), 21.3 (2 × CH_3), 18.1 (CH_3); **HRMS** (ESI): *m/z* = 475.2492, 497.2312, 971.4731, 1446.7182 calcd. for $[M+H]^+$, $[M+Na]^+$, $[2M+Na]^+$, $[3M+Na]^+$, found: 475.2493, 497.2315, 971.4742, 1446.7162.

6-(Naphthalen-1-yl)-2,4-diphenyl-5-(1-phenylethyl)-4,5-dihydro-1,2,4,5-tetrazin-3(2H)-one (18)

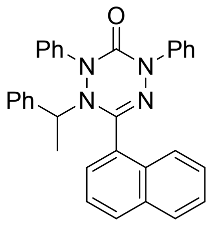

$C_{32}H_{26}N_4O$
482.58 g/mol

According to **GP4** with 1-bromoethylbenzene (260 µL, 1.91 mmol, 1.1 eq.), verdazyl radical **10** (600 mg, 1.59 mmol, 1.0 eq.), Cu (121 mg, 1.91 mmol, 1.1 eq.), $Cu(OTf)_2$ (11.5 mg, 31.8 µmol, 2 mol-%), 4,4'-di-*tert*-butyl-2,2'-bipyridine (17.1 mg, 62.6 µmol, 4 mol-%) in benzene (14 mL) at 80 °C for 20 h. FC (pentane/DCM, 5:1) afforded **18** (0.76 g, 1.57 mmol, 82%) as a colorless solid.

M.p.: 167 °C; **IR** (neat): 3060*w*, 2974*w*, 2932*w*, 1685*s*, 1593*m*, 1496*m*, 1485*s*, 1453*m*, 1362*w*, 1334*s*, 1318*m*, 1301*s*, 1253*w*, 1208*w*, 1177*w*, 1169*w*, 1136*w*, 1111*w*, 1086*m*, 1059*w*, 1026*w*, 1010*w*, 981*w*, 947*w*, 899*w*, 807*m*, 793*m*, 776*m*, 764m, 751*s*, 731*m*, 693*s*, 685*s*, 666*m*, 635*m*, 603*m*, 575*s*, 517*s;* **^{1}H NMR** (300 MHz, $CDCl_3$): δ = 8.91 (*s*, 1H, CH_{arom}), 8.33 (*s*, 1H, CH_{arom}), 8.04 (*d*, *J* = 8.2 Hz, 1H, CH_{arom}), 8.02 – 7.88 (*m*, 3H, CH_{arom}), 7.66 (*t*, *J* = 7.8 Hz, 1H, CH_{arom}), 7.60 (*dt*, *J* = 6.5, 3.4 Hz, 2H, CH_{arom}), 7.45 (*t*, *J* = 7.8 Hz, 2H, CH_{arom}), 7.40 – 7.28 (*m*, 7H, CH_{arom}), 7.24 – 7.16 (*m*, 4H, CH_{arom}), 4.57 (*q*, *J* = 7.2 Hz, 1H, CH), 1.49 (*d*, *J* = 7.2 Hz, 3H, CH_3); **^{13}C NMR** (75 MHz, $CDCl_3$) δ = 153.2 (C), 149.9 (C), 140.3 (2 × C), 139.0 (2 × C), 134.6 (C), 132.0 (CH), 131.7 (C), 129.0 (4 × CH), 128.9 (CH), 128.7 (CH), 128.7 (2 × CH), 128.3 (2 × CH), 128.0 (2 × CH), 127.6 (CH), 126.5 (CH), 126.0 (CH), 125.8 (CH), 125.1 (CH), 123.5 (4 × CH), 64.0 (CH), 18.5 (CH_3); **HRMS** (ESI): *m/z* = 483.2179, 505.1999 calcd. for $[M+H]^+$, $[M+Na]^+$, found: 483.2171, 505.1992.

6-(Anthracen-9-yl)-2,4-diphenyl-5-(1-phenylethyl)-4,5-dihydro-1,2,4,5-tetrazin-3(2H)-one (19)

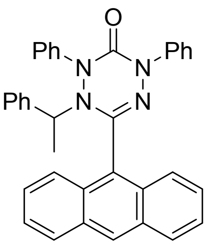

$C_{36}H_{28}N_4O$
532.63 g/mol

According to **GP4** with 1-bromoethylbenzene (100 µL, 0.73 mmol, 2.4 eq.), verdazyl radical **11** (130 mg, 0.30 mmol, 1.0 eq.), Cu (70 mg, 1.02 mmol, 3.1 eq.), $Cu(OTf)_2$ (3.4 mg, 9.4 µmol, 3 mol-%), 4,4'-di-*tert*-butyl-2,2'-bipyridine (5.8 mg, 21.6 µmol, 7 mol-%) in benzene (8 mL) at 80 °C for

20 h. FC (pentane/DCM, 5:1) afforded **19** (96 mg, 0.18 mmol, 61%) as a yellow solid.

M.p.: 141 °C; **IR** (neat): 3060*w*, 3030*w*, 2978*w*, 1692*s*, 1595*m*, 1492*m*, 1454*m*, 1343*s*, 1304*m*, 1239*w*, 1100*w*, 1068*w*, 1029*w*, 889*w*, 848*w*, 786*m*, 759*s*, 737*s*, 703*s*, 690*s*, 537*s*, 507*s*; **^{1}H NMR** (300 MHz, $CDCl_3$): δ = 8.77 (*s*, 1H, CH_{arom}), 8.51 (*br s*, 2H, CH_{arom}), 8.26 (*d*, *J* = 9.1 Hz, 2H, CH_{arom}), 8.09 – 8.03 (*m*, 2H, CH_{arom}), 7.79 – 7.66 (*m*, 6H, CH_{arom}), 7.64 – 7.30 (*m*, 10H, CH_{arom}), 7.25 (*s*, 1H, CH_{arom}), 4.75 (*q*, *J* = 7.1 Hz, 1H, CH), 1.66 (*d*, *J* = 7.1 Hz, 3H, CH_3); **^{13}C NMR** (75 MHz, $CDCl_3$): δ = 153.5 (C), 149.6 (C), 142.0 (C), 140.4 (2 × C), 139.0 (2 × C), 131.4 (2 × C), 131.2 (C), 130.5 (CH), 128.9 (2 × CH), 128.7 (2 × CH), 128.7 (2 × CH), 128.2 (CH), 128.1 (2 × CH), 128.1 (2 × CH), 126.9 (CH), 126.1 (2 × CH), 125.6 (2 × CH), 125.3 (CH), 124.6 (2 × CH), 123.8 (2 × CH), 120.6 (2 × CH), 63.1 (CH), 18.5 (CH_3); **HRMS** (ESI): *m/z* = 555.2155 calcd. for $[M+Na]^+$, found: 555.2161.

2,4-diphenyl-5-(1-phenylethyl)-6-(pyren-1-yl)-4,5-dihydro-1,2,4,5-tetrazin-3(2H)-one (20)

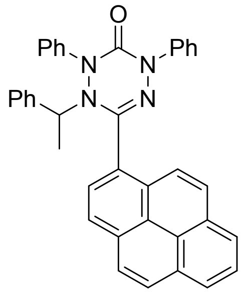

$C_{38}H_{28}N_4O$
556.66 g/mol

According to **GP4** with 1-bromoethylbenzene (81 µL, 0.60 mmol, 1.5 eq.), verdazyl radical **12** (0.180 g, 0.40 mmol, 1.0 eq.), Cu (30 mg, 0.48 mmol, 1.2 eq.), $Cu(OTf)_2$ (1.4 mg, 4.0 µmol, 2 mol-%), 4,4'-di-*tert*-butyl-2,2'-bipyridine (2.1 mg, 7.9 µmol, 4 mol-%) in benzene (14 mL) at 80 °C for 20 h. FC (pentane/DCM, 10:3) afforded **20** (210 mg, 0.37 mmol, 93%) as a colorless solid.

M.p.: 205 °C (decomp.); **IR** (neat): 3034*w*, 2963*w*, 1691*s*, 1596*m*, 1491*m*, 1455*m*, 1415*w*, 1378*w*, 1344*m*, 1304*m*, 1291*w*, 1260*s*, 1134*w*, 1089*s*, 1008*s*, 908*w*, 850*s*, 792*s*, 764*s*, 755*m*, 747*m*, 723*m*, 689*s*, 573*w*, 522*w*; **^{1}H NMR** (300 MHz, CD_2Cl_2): δ = 9.09 (*d*, *J* = 9.4 Hz, 1H, CH_{arom}), 8.74 (*br s*, 1H, CH_{arom}), 8.36 (*d*, *J* = 8.1 Hz, 1H, CH_{arom}), 8.30 (*dd*, *J* = 7.5, 3.4 Hz, 2H, CH_{arom}), 8.26 – 8.16 (*m*, 3H, CH_{arom}), 8.10 (*t*, *J* = 7.6 Hz, 1H, CH_{arom}), 7.98 (*br d*, *J* = 6.3 Hz, 2H, CH_{arom}), 7.50 (*t*, *J* = 7.8 Hz, 2H, CH_{arom}), 7.40 – 7.19 (*m*, 11H, CH_{arom}), 4.58 (*q*, *J* = 7.2 Hz, 1H, C*H*CH_3), 1.45 (*d*, *J* = 6.9 Hz, 3H, CH_3); **^{13}C NMR** (75 MHz, $CDCl_3$): δ = 153.2 (C), 150.6 (C), 140.4 (C), 139.1 (2 × C), 133.3 (C), 131.4 (2 × C), 130.8 (2 × C), 129.3 (CH), 129.2 (CH), 129.0 (2 × CH), 128.8 (CH), 128.7 (2 × CH), 128.4 (2 × CH), 128.0 (2 × CH), 127.4 (2 × CH), 126.6 (CH), 126.2 (CH), 126.0 (2 × CH), 125.9 (2 × CH), 125.7 (C), 125.2 (CH), 124.9 (CH), 124.8 (CH), 124.6 (C), 123.6 (2 × CH), 64.1 (CH), 18.5 (CH_3); **HRMS** (ESI): *m/z* = 579.2161, 1135.4424 calcd. for $[M+Na]^+$, $[2M+Na]^+$, found: 579.2154, 1135.4404.

6,6'-(1,4-phenylene)bis(2,4-diphenyl-5-(1-phenylethyl)-4,5-dihydro-1,2,4,5-tetrazin-3(2H)-one) (21)

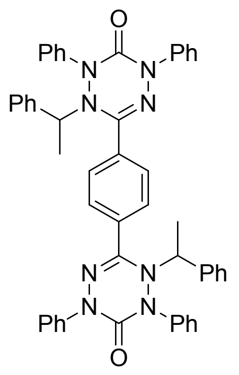

$C_{50}H_{42}N_8O_2$
786.92 g/mol

According to **GP4** with 1-bromoethylbenzene (200 µL, 1.41 mmol, 2.2 eq.), verdazyl radical **13** (400 mg, 0.69 mmol, 1.0 eq.), Cu (120 mg, 1.57 mmol, 2.3 eq.), $Cu(OTf)_2$ (5.0 mg, 13.9 µmol, 2.0 mol-%), 4,4'-di-*tert*-butyl-2,2'-bipyridine (16.0 mg, 59.7 µmol, 8.6 mol-%) in benzene (8 mL) at 80 °C for 20 h. FC (pentane/DCM, 3:1) afforded **21** (330 mg, 0.41 mmol, 60%) as a yellow solid.

M.p: 220 °C (decomp.); **IR** (neat): 3062*w*, 3032*w*, 2978*w*, 1776*w*, 1683*s*, 1596*m*, 1493*s,* 1454*m*, 1407*w*, 1335*s*, 1307*s*, 1290*s*, 1206*w*, 1182*m*, 1169*m*, 1113*m*, 1096*m*, 1071*w*, 1053*w*, 1030*w*, 1009*m*, 996*w*, 955*w*, 906*w*, 853*m*, 831*m*, 798*m*, 764*m*, 750*s*, 735*s*, 690*s*, 642*m*, 618*s*, 572*s*, 40*m*, 506*m*; **^{1}H NMR** (300 MHz, $CDCl_3$): δ = 8.27 – 8.06 (*m*, 4H, CH_{arom}), 7.97 – 7.71 (*m*, 4H, CH_{arom}), 7.52 – 7.33 (*m*, 14H, CH_{arom}), 7.32 – 7.12 (*m*, 9H, CH_{arom}), 7.01 (*d*, *J* = 7.8 Hz, 3H, CH_{arom}), 4.70 (*m*, 2H, C*H*CH_3), 1.67 – 1.52 (*m*, 6H, CH_3); **^{13}C NMR** (75 MHz, $CDCl_3$): δ = 152.7 (2 × C), 148.7 (2 × C), 144.5 (2 × C), 140.0 (2 × C), 138.5 (2 × C), 133.6 (2 × C), 128.8 (4 × CH), 128.7 (4 × CH), 128.2 (2 × CH), 128.1 (4 × CH), 127.8 (2 × CH), 125.9 (2 × CH), 125.1 (2 × CH), 123.6 (4 × CH), 123.6 (4 × CH), 121.5 (4 × CH), 120.6 (2 × CH), 63.7 (2 × CH), 18.1 (2 × CH_3); **HRMS** (ESI): *m/z* = 787.3503. 809.3323 calcd. for $[M+H]^+$, $[M+Na]^+$, found: 787.3486, 809.3306.

6,6'-(1,3-phenylene)bis(2,4-diphenyl-5-(1-phenylethyl)-4,5-dihydro-1,2,4,5-tetrazin-3(2H)-one) (22)

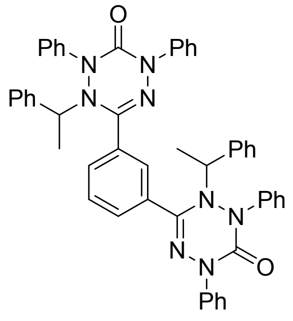

$C_{50}H_{42}N_8O_2$
786.92 g/mol

According to **GP4** with 1-bromoethylbenzene (53 µL, 0.37 mmol, 2.2 eq.), verdazyl radical **14** (100 mg, 0.17 mmol, 1.0 eq.), Cu (60 mg, 0.95 mmol, 5.6 eq.), $Cu(OTf)_2$ (1.5 mg, 4.2 µmol, 2.6 mol-%), 4,4'-di-*tert*-butyl-2,2'-bipyridine (3.7 mg, 13.8 µmol, 8.1 mol-%) in benzene (8 mL) at 80 °C for 20 h. FC (pentane/DCM, 3:1) afforded **22** (95 mg, 0.12 mmol, 68%) as a colorless solid.

M.p: 106 °C; **IR** (neat): 3065*w*, 2978*w*, 2937*w*, 1731*w*, 1689*m*, 1596*m*, 1486*m*, 1454*m*, 1333*m*, 1304*s*, 1240*s*, 1201*m*, 1178*s*, 1162*s*, 1098*s*, 10852*s*, 1053*w*, 983*m*, 903*w*, 808*w*, 750*m*, 689*s*, 628*m*, 600*m*, 571*m*, 529*m*, 502*s;* **^{1}H NMR** (300 MHz, $CDCl_3$): δ = 8.93 – 8.72 (*m*, 1H, CH_{arom}), 8.40 – 8.15 (*m*, 2H, CH_{arom}), 7.98 (*t*, *J* = 7.8 Hz, 3H, CH_{arom}), 7.85 – 7.64 (*m*, 2H, CH_{arom}), 7.58 –

7.50 (*m*, 1H, CH_{arom}), 7.45 – 7.16 (*m*, 22H, CH_{arom}), 7.08 (*t*, *J* = 6.4 Hz, 3H, CH_{arom}), 4.82 – 4.57 (*m*, 2H, C*H*CH_3), 1.69 – 1.52 (*m*, 6H, CHCH_3); **^{13}C NMR** (75 MHz, $CDCl_3$): δ = 152.7 (2 × C), 149.0 (2 × C), 144.6 (2 × C), 140.1 (2 × C), 138.6 (2 × C), 132.6 (2 × C), 130.4 (CH), 129.0 (4 × CH), 128.8 (4 × CH), 128.6 (2 × CH), 128.2 (4 × CH), 127.9 (4 × CH), 125.9 (CH), 125.1 (2 × CH), 123.6 (4 × CH), 123.5 (4 × CH), 121.4 (2 × CH), 121.3 (2 × CH), 63.8 (2 × CH), 18.4 (2 × CH_3); **HRMS** (ESI): *m/z* = 787.3503, 809.3323 calcd. for $[M+H]^+$, $[M+Na]^+$, found: 787.3505, 809.3330.

6,6',6"-(Benzene-1,3,5-triyl)tris(2,4-diphenyl-5-(1-phenylethyl)-4,5-dihydro-1,2,4,5-tetrazin-3(2H)-one) (23)

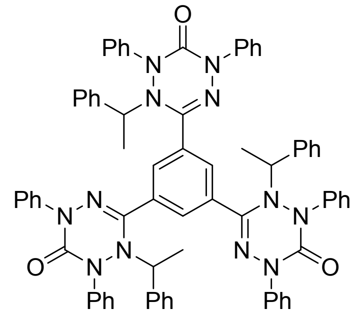

$C_{72}H_{60}N_{12}O_3$
1141.33 g/mol

According to **GP4** with 1-bromoethylbenzene (250 µL, 1.76 mmol, 4.8 eq.), verdazyl radical **15** (300 mg, 0.36 mmol, 1.0 eq.), Cu (90 mg, 1.41 mmol, 3.9 eq.), $Cu(OTf)_2$ (3.9 mg, 10.8 µmol, 3.0 mol-%), 4,4'-di-*tert*-butyl-2,2'-bipyridine (6.0 mg, 22.3 µmol, 6.2 mol-%) in benzene (8 mL) for 80 °C for 20 h. FC (pentane/DCM, 3:1) afforded **23** (260 mg, 0.23 mmol, 63%) as a colorless solid.

M.p.: 134 °C; **IR** (neat): 3064*w*, 3031*w*, 2980*w*, 2933*w*, 1692*s*, 1595*m*, 1488*s*, 1454*m*, 1332*s*, 1302*s*, 1204*w*, 1163*m*, 1107*w*, 1072*w*, 1054*w*, 1029*w*, 995*w*, 902*w*, 826*w*, 750*s*, 689*s*, 592*m*, 570*w*, 515m; **^{1}H NMR** (300 MHz, $CDCl_3$): δ = 8.99 – 8.70 (*m*, 3H, CH_{arom}), 7.96 (*d*, *J* = 7.8 Hz, 4H, CH_{arom}), 7.76 (*t*, *J* = 8.6 Hz, 2H, CH_{arom}), 7.60 (*s*, 2H, CH_{arom}), 7.48 – 7.30 (*m*, 24H, CH_{arom}), 7.25 – 7.16 (*m*, 5H, CH_{arom}), 7.16 – 6.98 (*m*, 8H, CH_{arom}), 4.88 – 4.61 (*m*, 3H, C*H*CH_3), 1.86 – 1.49 (*m*, 9H, CH_3); **^{13}C NMR** (75 MHz, $CDCl_3$): δ = 152.7 and 152.4 (3 × C), 149.0 and 148.1 (3 × C), 144.3 and 142.6 (3 × C), 140.2 and 139.9 (3 × C), 138.3 and 138.1 (3 × C), 133.8 and 133.2 (3 × C), 129.0 (6 × CH), 128.8 (6 × CH), 128.6 and 128.5 (6 × CH), 128.4 (2 × CH), 128.3 (2 × CH), 128.2 (4 × CH), 127.8 (4 × CH), 126.2 and 126.0 (3 × CH), 125.2 and 124.6 (3 × CH), 123.3 and 123.2 (6 × CH), 121.2 and 120.0 (6 × CH), 64.4 and 64.0 (3 × CH), 18.4 (3 × CH_3); **HRMS** (ESI): *m/z* = 1163.4828 calcd. for $[M+Na]^+$, found: 1163.4804.

2.7 Synthesis of methyl substituted tetrazin-3-one derivatives

2,4-Dimethyl-6-phenyl-1,2,4,5-tetrazinan-3-one (S7)

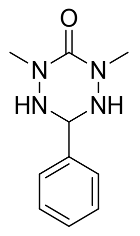

$C_{10}H_{14}N_4O$
206.24 g/mol

In a two-necked flask with condenser, **S4** (160 mg, 1.36 mmol, 1.0 eq.) was dissolved in MeOH (8 mL) and a solution of benzaldehyde (159 mg, 1.50 mmol, 1.1 eq.) in MeOH (2 mL) was added with a syringe pump at 60 °C within 30 min. After addition the mixture was stirred at 60 °C for 3 h and was then allowed to cool to RT. The solvent was removed *in vacuo* and the residue was recrystallized in hot EtOAc to afford colorless needles of 2,4-dimethyl-6-phenyl-1,2,4,5-tetrazinan-3-one (248 mg, 1.20 mmol, 88%).

^{1}H NMR (300 MHz, $CDCl_3$): δ = 7.56 – 7.49 (*m*, 2H, CH_{arom}), 7.44 – 7.31 (*m*, 3H, CH_{arom}), 5.04 (*s*, 1H, NC*H*N), 4.34 (*br s*, 2H, N*H*), 3.16 (*s*, 6H, CH_3); **HRMS** (ESI): *m/z* = 229.1060 calcd. for $[M+Na]^+$, found: 229.1069.
The obtained spectroscopic data are in accord with those reported in the literature.[5]

1,5-Dimethyl-3-phenyl-6-oxo-verdazyl radical (S8)

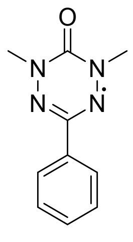

$C_{10}H_{11}N_4O^{\bullet}$
203.22 g/mol

In a round bottom flask **S7** (206 mg, 1.0 mmol, 1.0 eq.) was dissolved in a mixture of H_2O (5 mL) and MeOH (3.5 mL). A solution of $K_3Fe(CN)_6$ (1.086 g, 3.3 mmol, 3.3 eq.) and aq. Na_2CO_3 (2*N*, 1.5 mL) in H_2O (6 mL) was added. Immediately a red precipitate formed. The mixture was stirred for 40 min at RT and then the precipitate was filtered off, washed with H_2O and dried *in vacuo* to afford the 1,5-dimethyl-3-phenyl-6-oxo-verdazyl radical (135 mg, 0.67 mmol, 67%) as a red solid.

M.p.: 65 °C; **IR** (neat): 1685*s*, 1394*w*, 1295*w*, 1249*m*, 1014*m*, 925*w*, 776*s*, 716*m*, 692*s*, 657*s*, 541*s*, 534*s*, 516*w*; **HRMS** (ESI): *m/z* = 226.0825 calcd. for $[M+Na]^+$, found: 226.0828.
The obtained spectroscopic data are in accord with those reported in the literature.[5]

2,4-Dimethyl-6-phenyl-5-(1-phenylethyl)-4,5-dihydro-1,2,4,5-tetrazin-3(2H)-one (24)

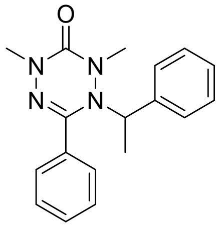

$C_{18}H_{20}N_4O$
308.38 g/mol

According to **GP4** with 1-bromoethylbenzene (319 µL, 2.34 mmol, 1.2 eq.), verdazyl radical **S8** (397 mg, 1.95 mmol, 1.0 eq.), Cu (149 mg, 2.34 mmol, 1.2 eq.), $Cu(OTf)_2$ (7 mg, 20 µmol, 1 mol-%), 4,4‘-di-*tert*-butyl-2,2‘-bipyridine (10.4 mg, 39 µmol, 2 mol-%) in benzene (7.5 mL) at 80 °C for

26 h. FC (pentane/MTBE, 3:1) afforded **24** (300 mg, 0.97 mmol, 50%) as a colorless sticky liquid.

IR (neat): 2976*w*, 2935*w*, 1673*s*, 1607*w*, 1448*m*, 1369*m*, 1282*w*, 1202*w*, 1072*m*, 1004*m*, 932*w*, 850*w*, 749*s*, 693*s*, 645*w*, 593*w*, 530*m*; **^{1}H NMR** (300 MHz, $CDCl_3$): δ = 7.92 – 7.82 (*m*, 2H, CH_{arom}), 7.49 – 7.40 (*m*, 3H, CH_{arom}), 7.30 (*s*, 5H, CH_{arom}), 4.35 (*d*, *J* = 7.3 Hz, 1H, CH), 3.19 – 2.68 (*br d*, 6H, NCH_3), 1.55 (*d*, *J* = 7.0 Hz, 3H, CH_3); **^{13}C NMR** (75 MHz, $CDCl_3$): δ = 157.5 (C), 147.7 (C), 139.2 (C), 132.0 (C), 130.4 (2 × CH), 128.9 (2 × CH), 128.3 (2 × CH), 128.2 (2 × CH), 127.7 (CH), 127.3 (CH), 62.2 (CH), 40.3 (CH_3), 36.0 (CH_3), 18.3 (CH_3); **HRMS** (ESI): *m/z* = 331.1529, 639.3166 calcd. for $[M+Na]^+$, $[2M+Na]^+$, found: 331.1532 , 639.3188.

5-Benzyl-2,4-dimethyl-6-phenyl-4,5-dihydro-1,2,4,5-tetrazin-3(2H)-one (24*)

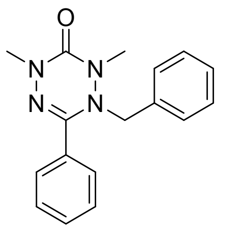

$C_{17}H_{18}N_4O$
294.35 g/mol

According to **GP4** with benzyl bromide (152 mg, 106 µL, 0.89 mmol, 1.2 eq.), verdazyl radical **S8** (150 mg, 0.74 mmol, 1.0 eq.), Cu (57 mg, 0.89 mmol, 1.2 eq.), $Cu(OTf)_2$ (2.7 mg, 7.4 µmol, 1 mol-%), 4,4‘-di-*tert*-butyl-2,2‘-bipyridine (4.0 mg, 14.8 µmol, 2 mol-%) in benzene (11 mL) at 80 °C for 20 h. FC (pentane/MTBE 2:1) afforded **24*** (80 mg, 0.27 mmol, 37%) as a purple solid.

M.p.: 74 °C; **IR** (neat): 2926*w*, 1666*s*, 1452*m*, 1371*m*, 1220*m*, 1066*w*, 977*w*, 743*m*, 701*s*, 592*m*; **^{1}H NMR** (300 MHz, $CDCl_3$): δ = 7.87 – 7.81 (*m*, 2H, CH_{arom}), 7.41 – 7.36 (*m*, 3H, CH_{arom}), 7.30 – 7.24 (*m*, 3H, CH_{arom}), 7.23 – 7.18 (*m*, 2H, CH_{arom}), 3.99 (*s*, 2H, CH_2), 2.98 (*s*, 3H, CH_3), 2.92 (*s*, 3H, CH_3); **^{13}C NMR** (75 MHz, $CDCl_3$): δ = 156.4 (C), 147.6 (C), 135.1 (C), 131.0 (C), 130.5 (CH), 129.9 (2 × CH), 128.9 (2 × CH), 128.5 (2 × CH), 128.4 (CH), 127.3 (2 × CH) , 55.6 (CH_2), 36.5 (CH_3), 35.9 (CH_3); **HRMS** (ESI): *m/z* = 317.1373, 611.2853 calcd. for $[M+Na]^+$, $[2M+Na]^+$, found: 317.1369, 611.2854.

6,6',6''-(Benzene-1,3,5-triyl)tris(2,4-dimethyl-1,2,4,5-tetrazinan-3-one) (S9)

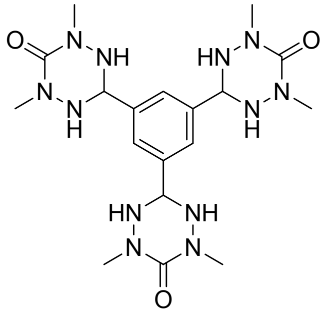

$C_{18}H_{30}N_{12}O_3$
462.51 g/mol

According to **GP2** with 1,3,5-triformylbenzene (**S6**) (0.30 g, 1.85 mmol, 1.0 eq.) and *N*,*N'*-dimethylcarbonohydrazide (0.70 g, 5.93 mmol, 3.1 eq.). Filtration afforded 6,6',6''-(benzene-1,3,5-triyl)tris-(2,4-dimethyl-1,2,4,5-tetrazinan-3-one) (0.55 g, 1.20 mmol, 65%) as a colorless solid.

IR (neat): 3458*m*, 3228*m*, 2930*m*, 1607*s*, 1505*m*, 1440*m*, 1388*s*, 1191*w*, 1119*m*, 1070*w*, 950*m*, 865*m*, 725*m*, 660*m*; **^{1}H NMR**

(300 MHz, DMSO-d_6): δ = 7.70 (*s*, 3H, CH_{arom}), 5.69 (*d*, *J* = 7.5 Hz, 6H, N*H*), 4.93 (*t*, *J* = 7.4 Hz, 3H, NC*H*N), 2.97 (*s*, 18H, CH_3); **^{13}C NMR** (75 MHz, DMSO-d_6): δ = 154.5 (3 × C), 136.8 (3 × C), 125.4 (3 × CH), 68.5 (3 × CH), 37.6 (6 × CH); **HRMS** (ESI): *m/z* = 463.2637, 485.2456 calcd. for $[M+H]^+$, $[M+Na]^+$, found: 463.2641, 485.2459.

1,3,5-Tris(1,5-dimethyl-6-oxo-3-verdazyl)benzene (S10)

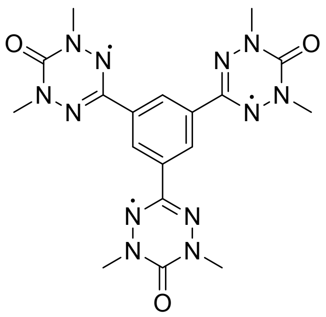

$C_{18}H_{21}N_{12}O_3^{3\bullet}$
453.44 g/mol

According to **GP3** with **S9** (0.53 g, 1.15 mmol, 1.0 eq.) and 1,4-benzoquinone (0.62 g, 5.85 mmol, 5.1 eq.) in DCM (15 mL). Filtration afforded 1,3,5-tris(1,5-dimethyl-6-oxo-3-verdazyl)-benzene (0.15 g, 0.33 mmol, 29%) as a red solid.

M.p.: 76 °C; **IR** (neat): 3230*w*, 2939*w*, 1678*s*, 1612*m*, 1507*w*, 1443*w*, 1392*m*, 1322*w*, 1289*m*, 1250*w*, 1116*w*, 1047*w*, 975*m*, 954*w*, 904*m*, 875*w*, 836*w*, 719*s*, 691*s*, 655*m*, 537*s*; **HRMS** (ESI): *m/z* = 501.21954 calcd. for $[M+Na]^+$, found: 501.21931.

6,6',6''-(Benzene-1,3,5-triyl)tris(1,4-dihydro-2,4-dimethyl-5-(1-phenylethyl)-1,2,4,5-tetrazinan-3-one) (25)

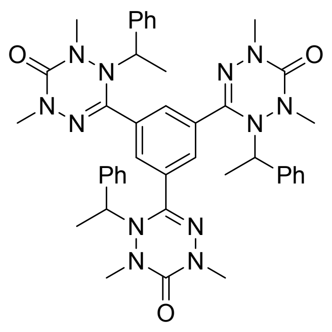

$C_{42}H_{48}N_{12}O_3$
768.91 g/mol

According to **GP4** with 1-bromoethylbenzene (200 µL, 1.41 mmol, 6.3 eq.), verdazyl triradical **S10** (100 mg, 0.22 mmol, 1.0 eq.), Cu (150 mg, 2.36 mmol, 10.7 eq.), $Cu(OTf)_2$ (1.7 mg, 4.7 µmol, 2.1 mol-%), 4,4'-di-*tert*-butyl-2,2'-bipyridine (5.2 mg, 19.4 µmol, 8.8 mol-%) in benzene (5 mL) at 80 °C for 20 h. FC (EtOAc/pentane, 3:1) afforded **25** (0.16 g, 0.20 mmol, 91%) as a colorless solid.

M.p.: 82 °C; **IR** (neat): 3031*w*, 2979*w*, 2932*w*, 1774*w*, 1674*s*, 1585*w*, 1495*w*, 1453*m*, 1422*m*, 1403*w*, 1369*s*, 1264*m*, 1202*m*, 1126*w*, 1060*m*, 1024*m*, 986*m*, 903*m*, 856*w*, 796*w*, 755*s*, 732*m*, 697*s*, 661*m*, 601*w*, 587*w*, 554*s*, 531*s;* **^{1}H NMR** (300 MHz, $CDCl_3$): δ = 8.42 (*s*, 3H, CH_{arom}), 7.32 (*br s*, 15H, CH_{arom}),4.45 (*s*, 3H, C*H*CH_3), 3.53 – 2.67 (*m*, 18H, NCH_3), 1.61 (*d*, *J* = 7.0 Hz, 9H, CHCH_3); **^{13}C NMR** (101 MHz, $CDCl_3$): δ = 158.0 and 156.9 (3 × C), 146.5 and 146.1 (3 × C), 139.2 (3 × C), 133.5 (3 × C), 128.5 (6 × CH), 128.4 (6 × CH), 127.7 (3 × CH), 126.8 (3 × CH), 62.6 and 62.2 (3 × CH), 39.9

and 39.3 (3 × CH_3), 36.9 and 36.4 (3 × CH_3), 19.0 and 15.5 (3 × CH_3); **HRMS** (ESI): *m/z* = 791.38645 calcd. for $[M+Na]^+$; found: 791.38682.

3 EPR spectra

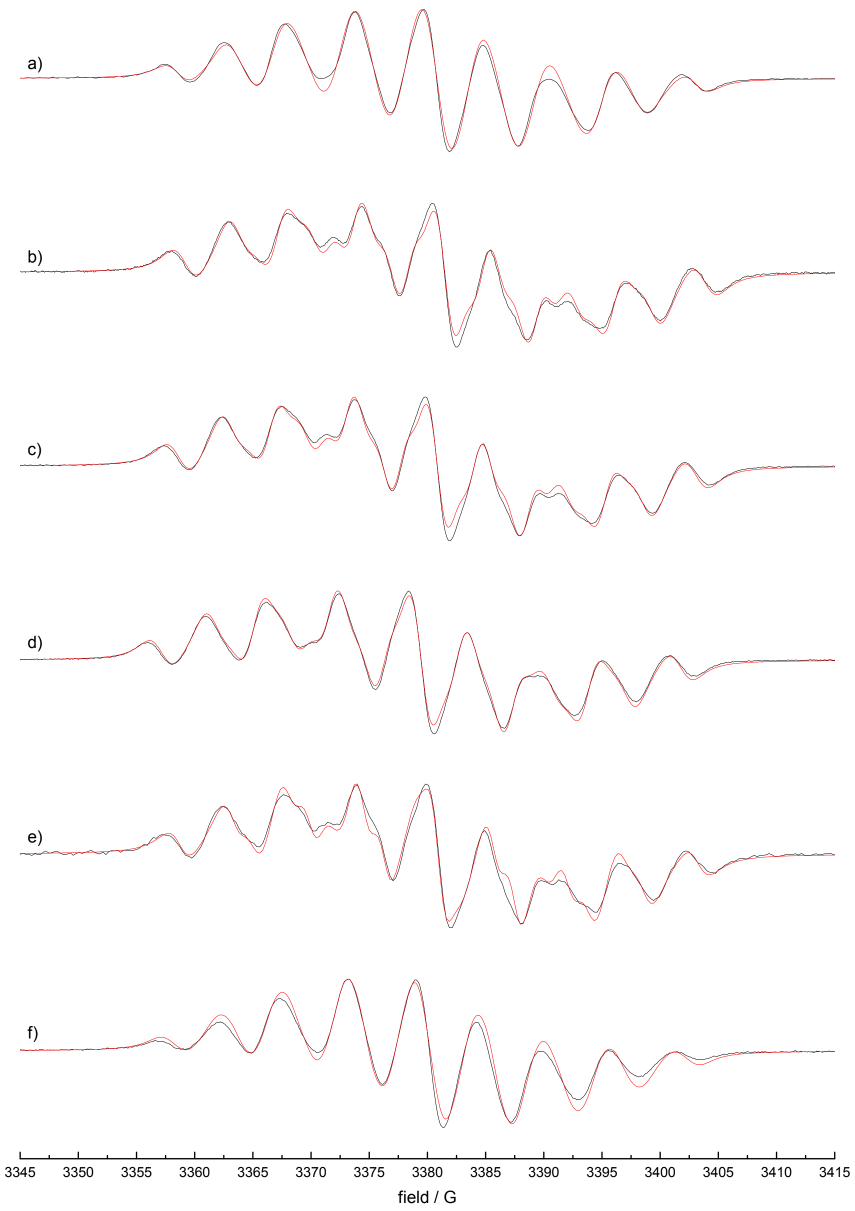

Figure S1: EPR spectra (black) and appending simulations (red) of verdazyl radicals:
a) 1,5-di-(4-methoxy)phenyl-3-phenyl-6-oxo-verdazyl (**8***), b) 1,5-diphenyl-3-mesityl-6-oxo-verdazyl (**9**), c) 1,5-diphenyl-3-(1'-naphthyl)-6-oxo-verdazyl (**10**), d) 1,5-diphenyl-3-(9'-anthracenyl)-6-oxo-verdazyl (**11**), e) 1,5-diphenyl-3-(1'-pyrenyl)-6-oxo-verdazyl (**12**) f) 1,3-bis(1,5-di-(4-octyloxy)phenyl-6-oxo-3-verdazyl)benzene (**14***).

Table S1: Summary of the A_{iso} values (in Gauss) and g-values.

		A(N-1,5)	A(N-2,4)	g-value
**8***	1,5-Di-(4-methoxy)phenyl-3-phenyl-6-oxo-verdazyl	5.112	5.901	2.00375
**9**	1,5-Diphenyl-3-mesityl-6-oxo-verdazyl	4.593	6.366	2.00368
**10**	1,5-Diphenyl-3-(1'-naphthyl)-6-oxo-verdazyl	4.767	6.383	2.00385
**11**	1,5-Diphenyl-3-(9'-anthracenyl)-6-oxo-verdazyl	5.069	5.973	2.0036
**12**	1,5-Diphenyl-3-(1'-pyrenyl)-6-oxo-verdazyl	5.063	5.973	2.0036
**14***	1,3-Bis(1,5-di-(4-octyloxy)phenyl-6-oxo-3-verdazyl)benzene	5.032	5.901	2.00375

4 Photophysical measurements

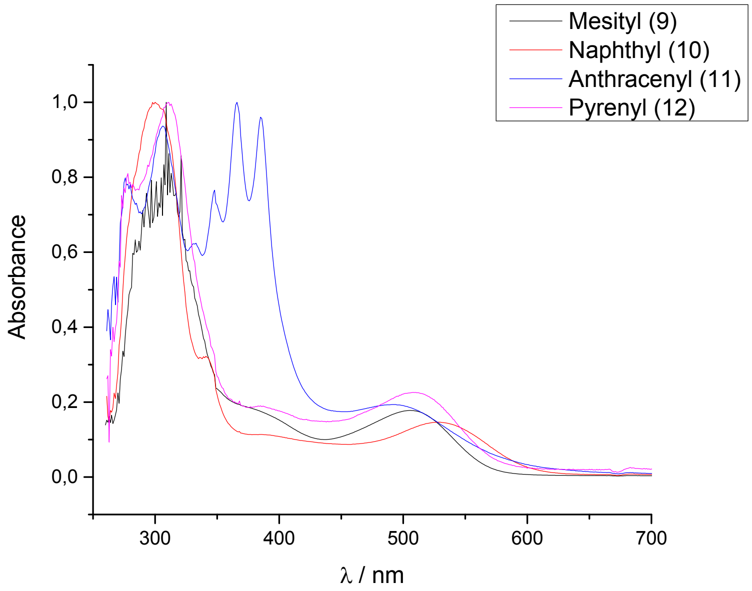

Figure S2: UV/Vis-spectra of the verdazyl radicals **9** – **12**.

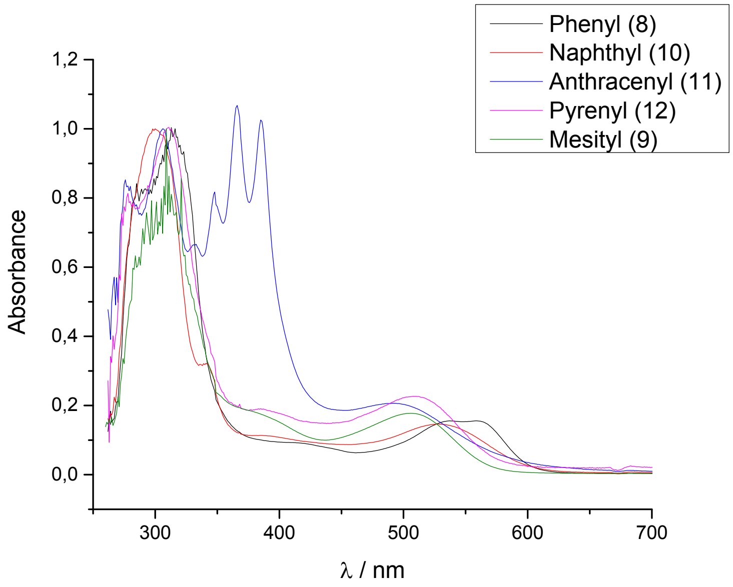

Figure S3 UV/Vis-spectra of the verdazyl radicals **8** – **12.**

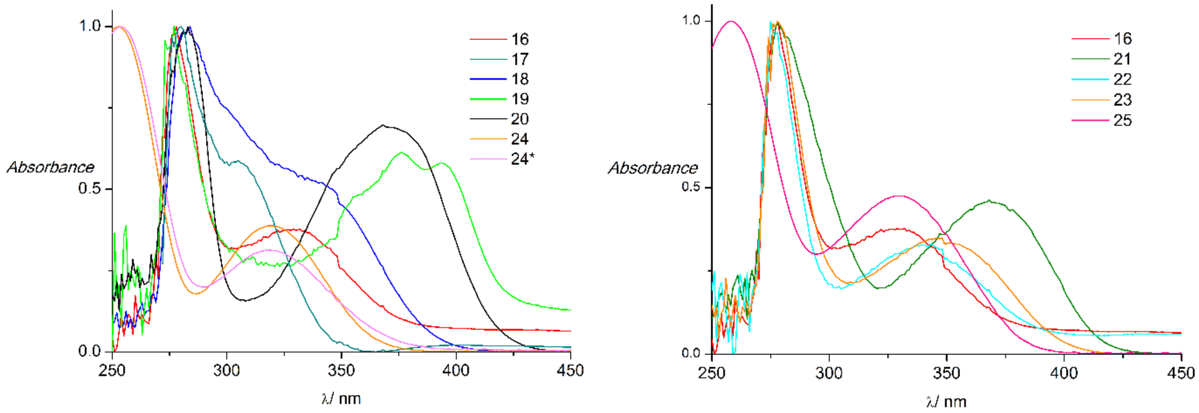

Figure S4: Absorption spectra in *acetonitrile*.

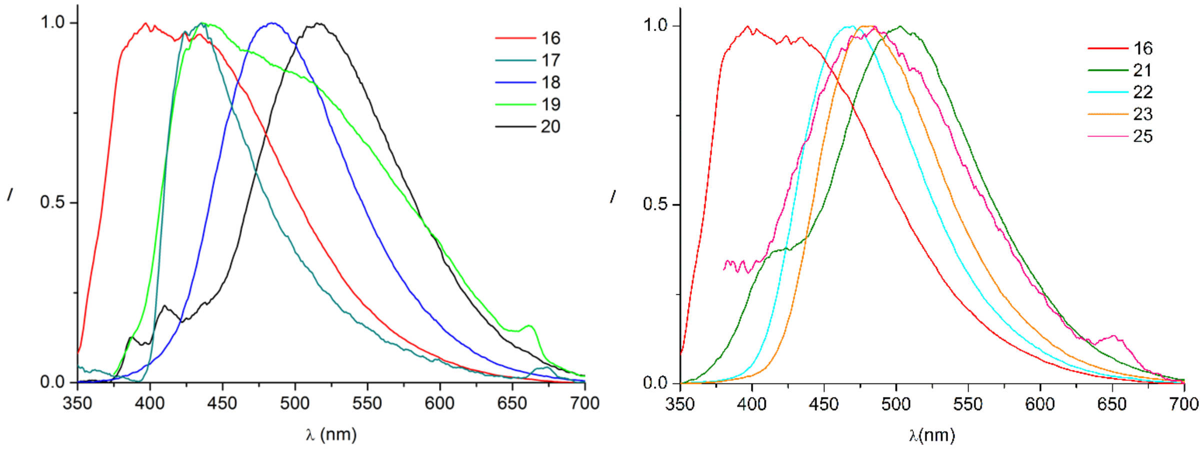

Figure S5: Emission spectra in *acetonitrile* (λ_{Exc} = 325 nm).

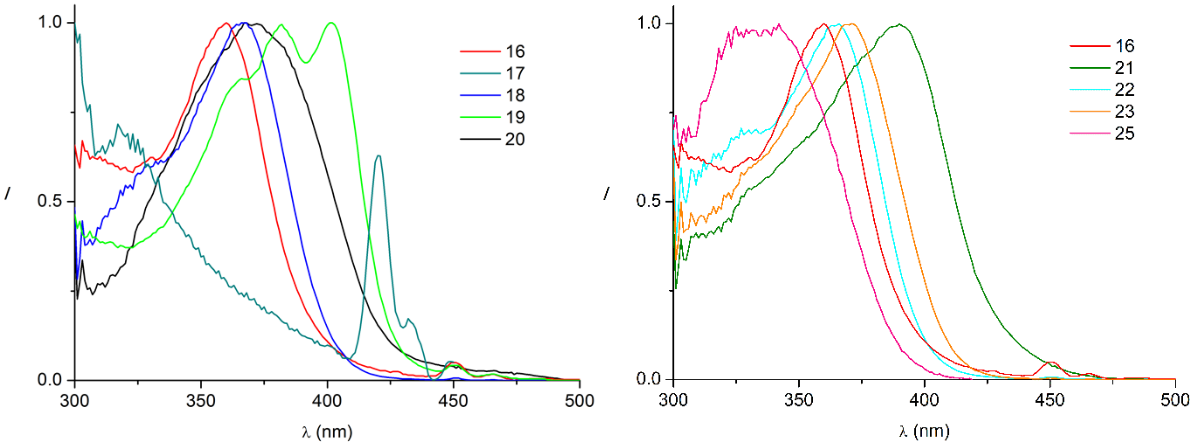

Figure S6: Excitation spectra in *acetonitrile* (λ_{Em} = 520 nm).

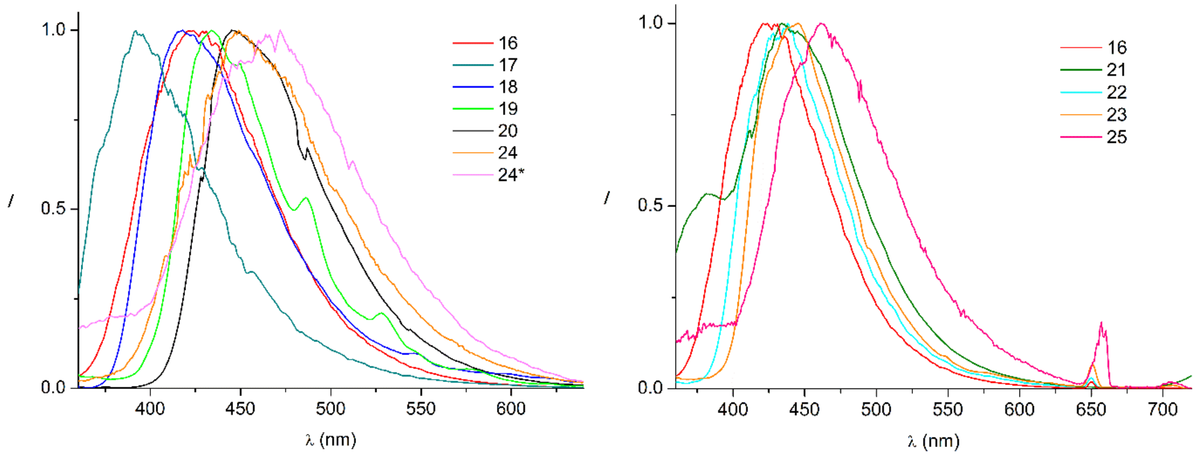

Figure S7: Emission spectra in *butyronitrile* at 77 K (λ_{Exc} = 325 nm).

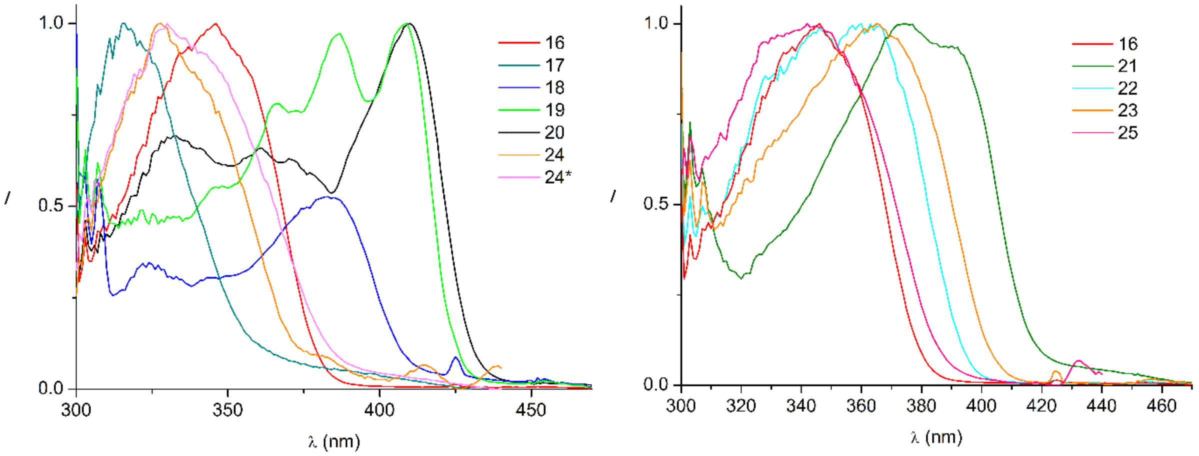

Figure S8: Excitation spectra in *butyronitrile* at 77 K (λ_{Em} = 520 nm).

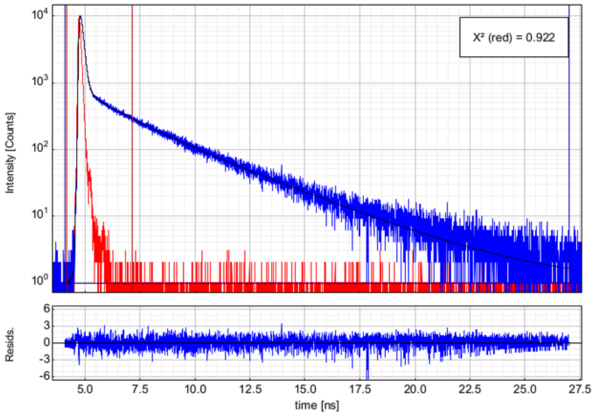

Parameter	Value	Conf. $_{Lower}$	Conf. $_{Upper}$
A_1 [Cnts]	441.3	-10.1	+10.1
τ_1 [ns]	3.3971	-0.0513	+0.0513
A_2 [Cnts]	24604	-416	+416
τ_2 [ns]	0.08801	-0.00135	+0.00135
A_3 [Cnts]	360.9	-23.8	+23.8
τ_3 [ns]	1.4422	-0.0822	+0.0822
Bkgr. $_{Dec}$ [Cnts]	0.974	-0.471	+0.471
Bkgr. $_{IRF}$ [Cnts]	0.237	-1.34	+1.34
Shift $_{IRF}$ [ns]	-0.08454	-0.00128	+0.00128

Figure S9: Left: Time-resolved luminescence decay of **16** in *acetonitrile* including the residuals and IRF (λ_{exc} = 376.7 nm). Right: Fitting parameters including pre-exponential factors and confidence limits.

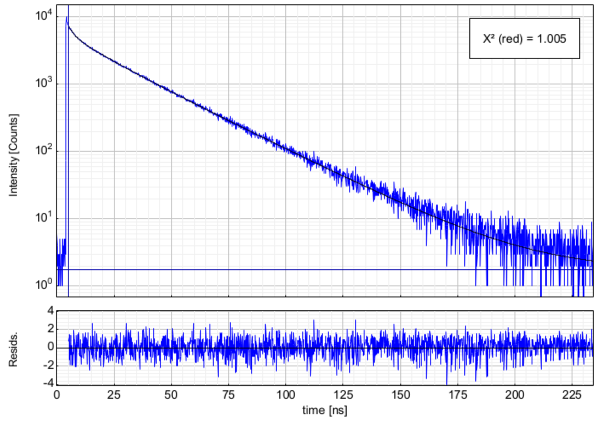

Parameter	Value	Conf. $_{Lower}$	Conf. $_{Upper}$
A_1 [Cnts]	4075.5	-32.5	+32.5
τ_1 [ns]	26.111	-0.142	+0.142
A_2 [Cnts]	1878	-156	+156
τ_2 [ns]	2.769	-0.294	+0.294
A_3 [Cnts]	1213.5	-59.4	+59.4
τ_3 [ns]	13.052	-0.629	+0.629
Bkgr. $_{Dec}$ [Cnts]	1.743	-0.630	+0.630

Figure S10: Left: Time-resolved luminescence decay of **16** in a frozen *butyronitrile* glassy matrix at 77 K including the residuals (λ_{exc} = 376.7 nm). Right: Fitting parameters including pre-exponential factors and confidence limits.

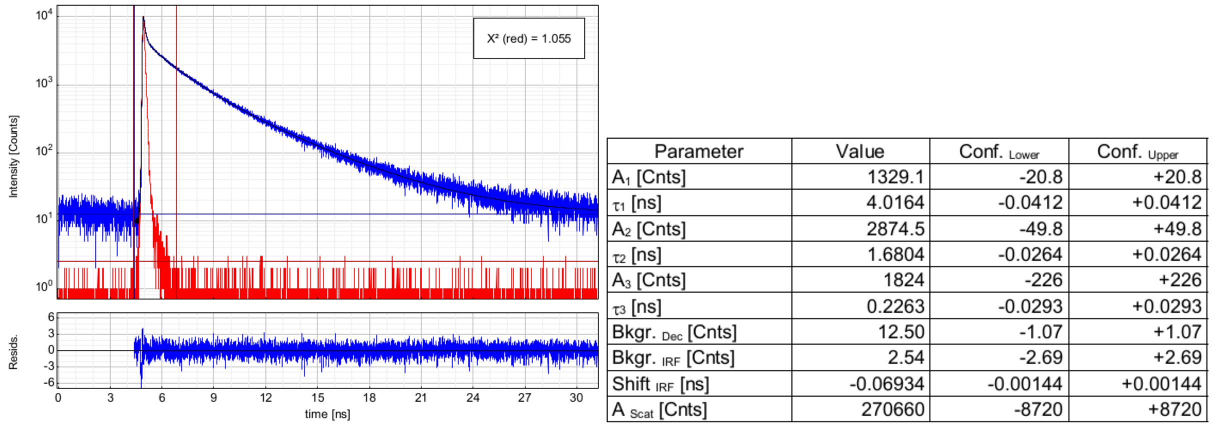

Parameter	Value	Conf. $_{Lower}$	Conf. $_{Upper}$
A_1 [Cnts]	1329.1	-20.8	+20.8
τ_1 [ns]	4.0164	-0.0412	+0.0412
A_2 [Cnts]	2874.5	-49.8	+49.8
τ_2 [ns]	1.6804	-0.0264	+0.0264
A_3 [Cnts]	1824	-226	+226
τ_3 [ns]	0.2263	-0.0293	+0.0293
Bkgr. $_{Dec}$ [Cnts]	12.50	-1.07	+1.07
Bkgr. $_{IRF}$ [Cnts]	2.54	-2.69	+2.69
Shift $_{IRF}$ [ns]	-0.06934	-0.00144	+0.00144
A $_{Scat}$ [Cnts]	270660	-8720	+8720

Figure S11: Left: Time-resolved luminescence decay of **17** in *acetonitrile* including the residuals and IRF (λ_{exc} = 376.7 nm). Right: Fitting parameters including pre-exponential factors and confidence limits.

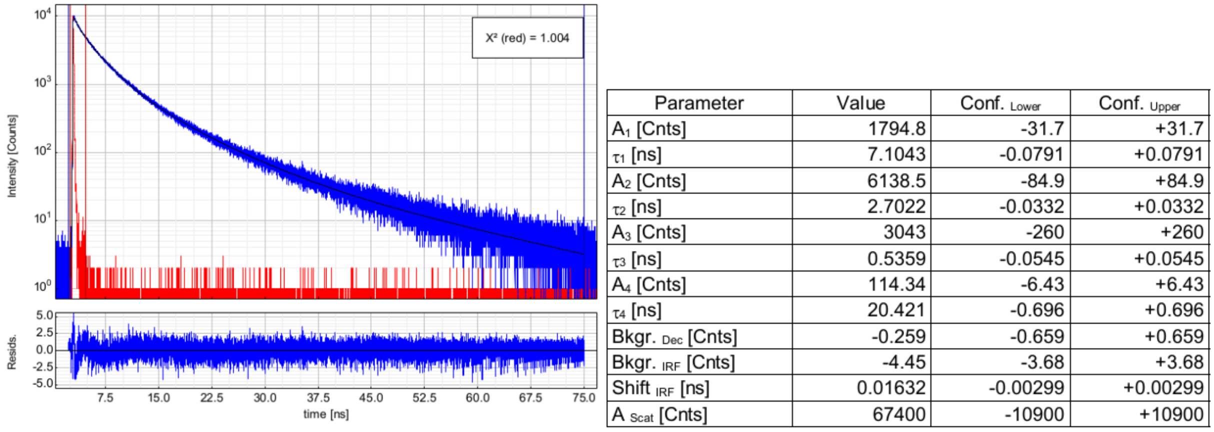

Parameter	Value	Conf. $_{Lower}$	Conf. $_{Upper}$
A_1 [Cnts]	1794.8	-31.7	+31.7
τ_1 [ns]	7.1043	-0.0791	+0.0791
A_2 [Cnts]	6138.5	-84.9	+84.9
τ_2 [ns]	2.7022	-0.0332	+0.0332
A_3 [Cnts]	3043	-260	+260
τ_3 [ns]	0.5359	-0.0545	+0.0545
A_4 [Cnts]	114.34	-6.43	+6.43
τ_4 [ns]	20.421	-0.696	+0.696
Bkgr. $_{Dec}$ [Cnts]	-0.259	-0.659	+0.659
Bkgr. $_{IRF}$ [Cnts]	-4.45	-3.68	+3.68
Shift $_{IRF}$ [ns]	0.01632	-0.00299	+0.00299
A $_{Scat}$ [Cnts]	67400	-10900	+10900

Figure S12: Left: Time-resolved luminescence decay of **17** in a frozen *butyronitrile* glassy matrix at 77 K including the residuals (λ_{exc} =376.7 nm). Right: Fitting parameters including pre-exponential factors and confidence limits.

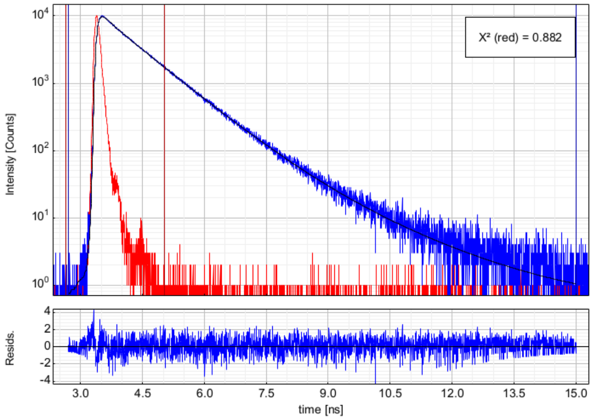

Parameter	Value	Conf. $_{Lower}$	Conf. $_{Upper}$
A_1 [Cnts]	2035	-195	+195
τ_1 [ns]	0.1716	-0.0201	+0.0201
A_2 [Cnts]	10694.5	-61.4	+61.4
τ_2 [ns]	0.84825	-0.00362	+0.00362
A_3 [Cnts]	324.9	-21.0	+21.0
τ_3 [ns]	1.8003	-0.0489	+0.0489
Bkgr. $_{Dec}$ [Cnts]	0.515	-0.429	+0.429
Bkgr. $_{IRF}$ [Cnts]	-0.0379	-0.492	+0.492
Shift $_{IRF}$ [ns]	-0.05272	-0.00111	+0.00111

Figure S13: Left: Time-resolved luminescence decay of **18** in *acetonitrile* including the residuals and IRF (λ_{exc} = 376.7 nm). Right: Fitting parameters including pre-exponential factors and confidence limits.

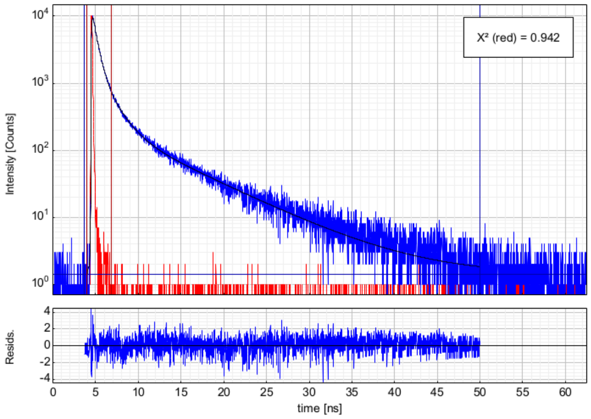

Parameter	Value	Conf. $_{Lower}$	Conf. $_{Upper}$
A_1 [Cnts]	271.06	-8.87	+8.87
τ_1 [ns]	7.063	-0.143	+0.143
A_2 [Cnts]	2071.3	-62.3	+62.3
τ_2 [ns]	1.5163	-0.0319	+0.0319
A_3 [Cnts]	10779	-191	+191
τ_3 [ns]	0.48435	-0.00798	+0.00798
Bkgr. $_{Dec}$ [Cnts]	1.403	-0.456	+0.456
Bkgr. $_{IRF}$ [Cnts]	-0.109	-1.48	+1.48
Shift $_{IRF}$ [ns]	-0.01945	-0.00206	+0.00206

Figure S14: Left: Time-resolved luminescence decay of **18** in a frozen *butyronitrile* glassy matrix at 77 K including the residuals and IRF (λ_{exc} = 376.7 nm). Right: Fitting parameters including pre-exponential factors and confidence limits.

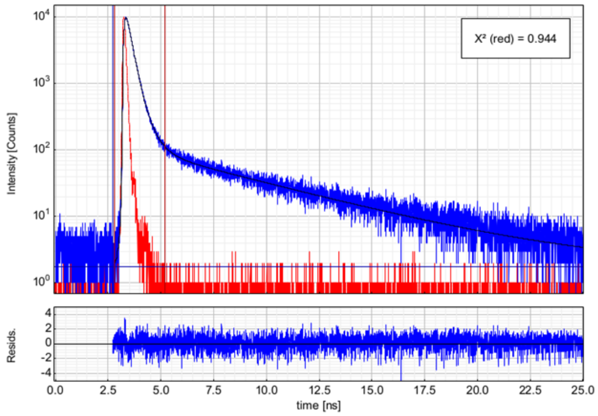

Parameter	Value	Conf. $_{Lower}$	Conf. $_{Upper}$
A_1 [Cnts]	1035.7	-79.0	+79.0
τ_1 [ns]	0.5226	-0.0250	+0.0250
A_2 [Cnts]	15330	-201	+201
τ_2 [ns]	0.23345	-0.00254	+0.00254
A_3 [Cnts]	112.19	-4.23	+4.23
τ_3 [ns]	5.165	-0.147	+0.147
Bkgr. $_{Dec}$ [Cnts]	1.786	-0.555	+0.555
Bkgr. $_{IRF}$ [Cnts]	-0.239	-1.55	+1.55
Shift $_{IRF}$ [ns]	-0.08370	-0.00145	+0.00145

Figure S15: Left: Time-resolved luminescence decay of **19** in *acetonitrile* including the residuals and IRF (λ_{exc} = 376.7 nm). Right: Fitting parameters including pre-exponential factors and confidence limits.

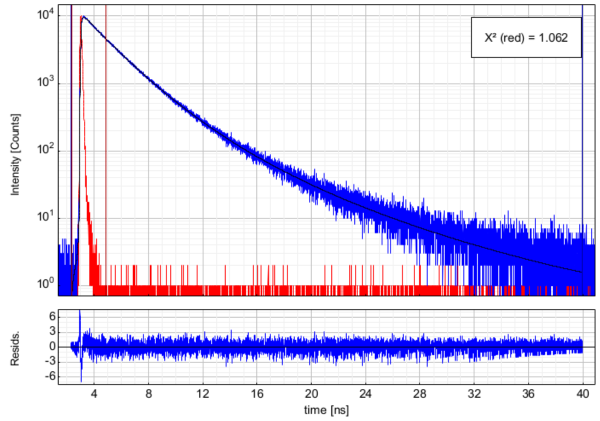

Parameter	Value	Conf. $_{Lower}$	Conf. $_{Upper}$
A_1 [Cnts]	181.3	-12.6	+12.6
τ_1 [ns]	7.183	-0.236	+0.236
A_2 [Cnts]	8407.2	-82.8	+82.8
τ_2 [ns]	1.8374	-0.0155	+0.0155
A_3 [Cnts]	2366.5	-46.1	+46.1
τ_3 [ns]	3.2574	-0.0389	+0.0389
Bkgr. $_{Dec}$ [Cnts]	0.480	-0.535	+0.535
Bkgr. $_{IRF}$ [Cnts]	-0.545	-0.976	+0.976
Shift $_{IRF}$ [ns]	-0.01342	-0.00227	+0.00227

Figure S16: Left: Time-resolved luminescence decay of **19** in a frozen *butyronitrile* glassy matrix at 77 K including the residuals and IRF (λ_{exc} = 376.7 nm). Right: Fitting parameters including pre-exponential factors and confidence limits.

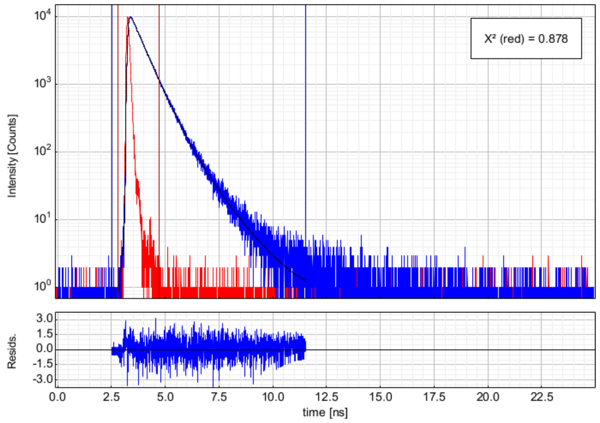

Parameter	Value	Conf. $_{Lower}$	Conf. $_{Upper}$
A_1 [Cnts]	12242.3	-71.2	+71.2
τ_1 [ns]	0.54684	-0.00250	+0.00250
A_2 [Cnts]	939.8	-28.8	+28.8
τ_2 [ns]	1.1130	-0.0159	+0.0159
Bkgr. $_{Dec}$ [Cnts]	0.696	-0.446	+0.446
Bkgr. $_{IRF}$ [Cnts]	-0.82	-1.13	+1.13
Shift $_{IRF}$ [ns]	-0.099785	-0.000964	+0.000964

Figure S17: Left: Time-resolved luminescence decay of **20** in *acetonitrile* including the residuals and IRF (λ_{exc} = 376.7 nm). Right: Fitting parameters including pre-exponential factors and confidence limits.

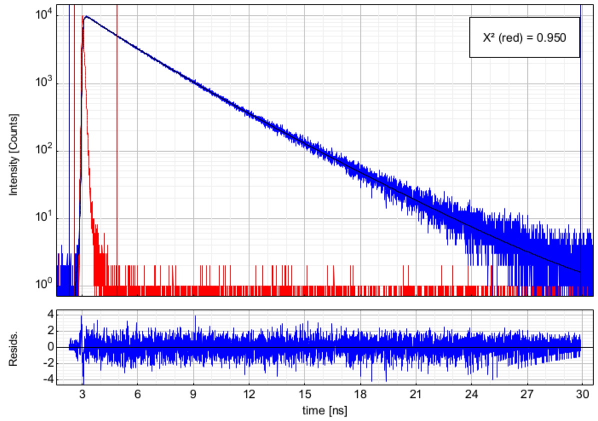

Parameter	Value	Conf. $_{Lower}$	Conf. $_{Upper}$
A_1 [Cnts]	935.7	-34.0	+34.0
τ_1 [ns]	3.7145	-0.0736	+0.0736
A_2 [Cnts]	5282.9	-46.3	+46.3
τ_2 [ns]	2.8813	-0.0170	+0.0170
A_3 [Cnts]	4626.6	-68.7	+68.7
τ_3 [ns]	1.8724	-0.0249	+0.0249
Bkgr. $_{Dec}$ [Cnts]	0.432	-0.589	+0.589
Bkgr. $_{IRF}$ [Cnts]	-1.21	-1.64	+1.64
Shift $_{IRF}$ [ns]	-0.01775	-0.00183	+0.00183

Figure S18: Left: Time-resolved luminescence decay of **20** in a frozen *butyronitrile* glassy matrix at 77 K including the residuals and IRF (λ_{exc} = 376.7 nm). Right: Fitting parameters including pre-exponential factors and confidence limits.

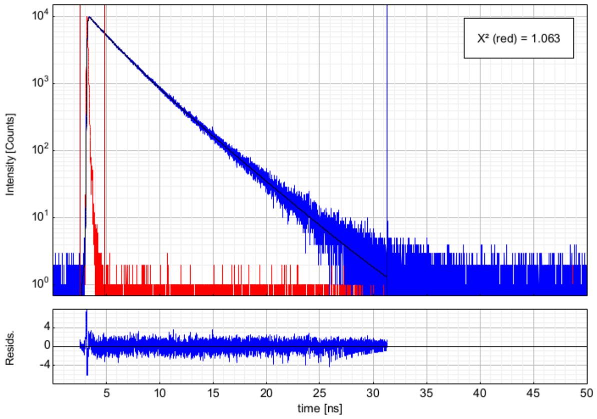

Parameter	Value	Conf. $_{\text{Lower}}$	Conf. $_{\text{Upper}}$
A_1 [Cnts]	5582.5	-44.1	+44.1
τ_1 [ns]	3.2942	-0.0164	+0.0164
A_2 [Cnts]	5291.1	-74.9	+74.9
τ_2 [ns]	1.8678	-0.0242	+0.0242
Bkgr. $_{\text{Dec}}$ [Cnts]	0.192	-0.613	+0.613
Bkgr. $_{\text{IRF}}$ [Cnts]	-0.506	-0.963	+0.963
Shift $_{\text{IRF}}$ [ns]	0.04962	-0.00202	+0.00202

Figure S19: Left: Time-resolved luminescence decay of **21** in *acetonitrile* including the residuals and IRF (λ_{exc} = 376.7 nm). Right: Fitting parameters including pre-exponential factors and confidence limits.

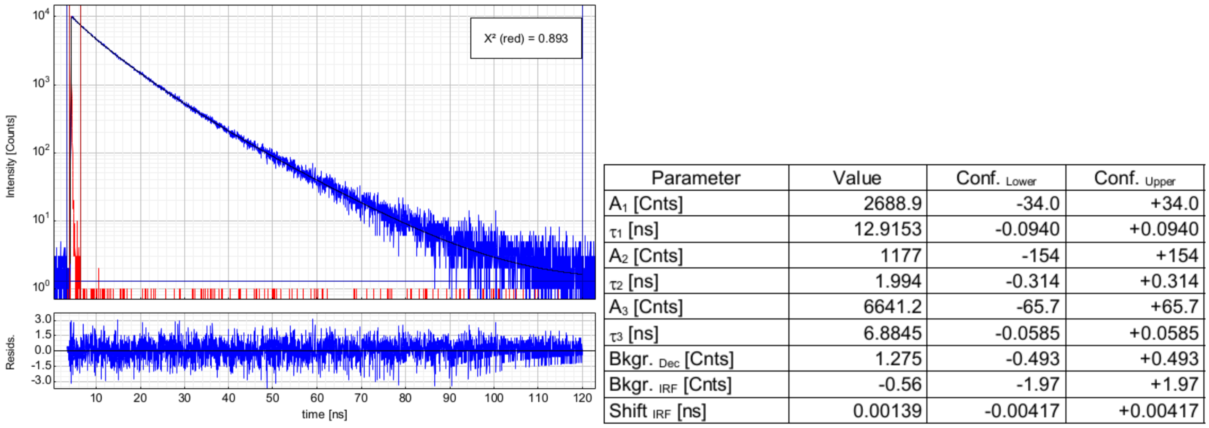

Parameter	Value	Conf. $_{\text{Lower}}$	Conf. $_{\text{Upper}}$
A_1 [Cnts]	2688.9	-34.0	+34.0
τ_1 [ns]	12.9153	-0.0940	+0.0940
A_2 [Cnts]	1177	-154	+154
τ_2 [ns]	1.994	-0.314	+0.314
A_3 [Cnts]	6641.2	-65.7	+65.7
τ_3 [ns]	6.8845	-0.0585	+0.0585
Bkgr. $_{\text{Dec}}$ [Cnts]	1.275	-0.493	+0.493
Bkgr. $_{\text{IRF}}$ [Cnts]	-0.56	-1.97	+1.97
Shift $_{\text{IRF}}$ [ns]	0.00139	-0.00417	+0.00417

Figure S20: Left: Time-resolved luminescence decay of **21** in a frozen *butyronitrile* glassy matrix at 77 K including the residuals and IRF (λ_{exc} = 376.7 nm). Right: Fitting parameters including pre-exponential factors and confidence limits.

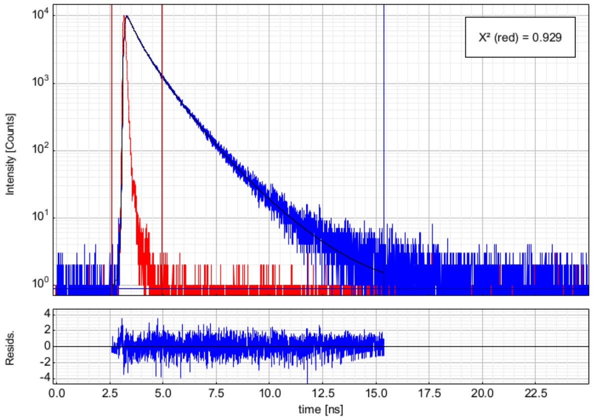

Parameter	Value	Conf. $_{\text{Lower}}$	Conf. $_{\text{Upper}}$
A_1 [Cnts]	6597.4	-56.5	+56.5
τ_1 [ns]	0.92322	-0.00566	+0.00566
A_2 [Cnts]	6013	-129	+129
τ_2 [ns]	0.34114	-0.00775	+0.00775
A_3 [Cnts]	684.9	-24.2	+24.2
τ_3 [ns]	1.7410	-0.0296	+0.0296
Bkgr. $_{\text{Dec}}$ [Cnts]	0.893	-0.492	+0.492
Bkgr. $_{\text{IRF}}$ [Cnts]	-0.0396	-0.702	+0.702
Shift $_{\text{IRF}}$ [ns]	-0.16642	-0.00116	+0.00116

Figure S21: Left: Time-resolved luminescence decay of **22** in *acetonitrile* including the residuals and IRF (λ_{exc} = 376.7 nm). Right: Fitting parameters including pre-exponential factors and confidence limits.

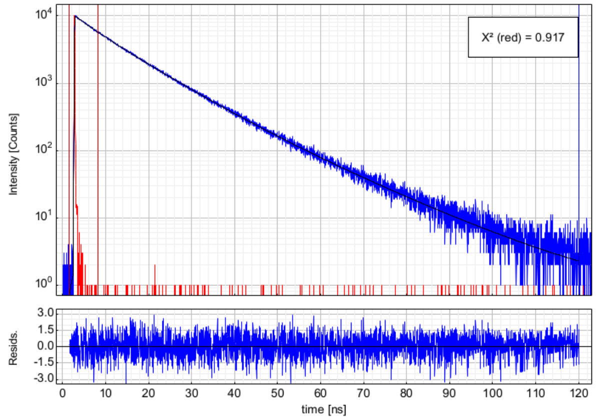

Parameter	Value	Conf. $_{Lower}$	Conf. $_{Upper}$
A_1 [Cnts]	1830.9	-30.2	+30.2
τ_1 [ns]	16.590	-0.151	+0.151
A_2 [Cnts]	7596.8	-55.1	+55.1
τ_2 [ns]	9.6590	-0.0565	+0.0565
A_3 [Cnts]	875	-126	+126
τ_3 [ns]	2.993	-0.512	+0.512
Bkgr. $_{Dec}$ [Cnts]	0.684	-0.640	+0.640
Bkgr. $_{IRF}$ [Cnts]	-0.111	-0.567	+0.567
Shift $_{IRF}$ [ns]	0.02486	-0.00329	+0.00329

Figure S22: Left: Time-resolved luminescence decay of **22** in a frozen *butyronitrile* glassy matrix at 77 K including the residuals and IRF (λ_{exc} = 376.7 nm). Right: Fitting parameters including pre-exponential factors and confidence limits.

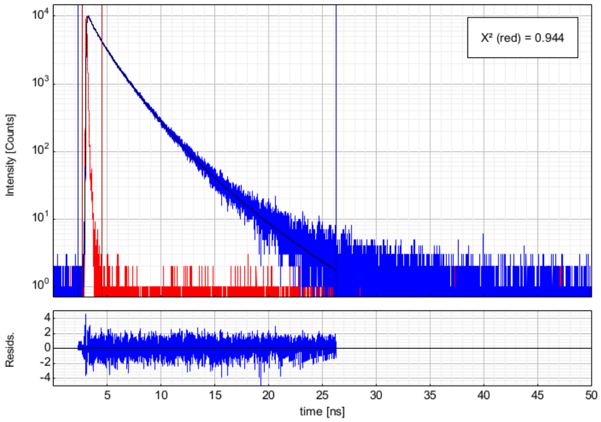

Parameter	Value	Conf. $_{Lower}$	Conf. $_{Upper}$
A_1 [Cnts]	843.0	-23.0	+23.0
τ_1 [ns]	3.6130	-0.0471	+0.0471
A_2 [Cnts]	7308.0	-56.7	+56.7
τ_2 [ns]	1.7771	-0.0106	+0.0106
A_3 [Cnts]	3509	-125	+125
τ_3 [ns]	0.6386	-0.0252	+0.0252
Bkgr. $_{Dec}$ [Cnts]	0.361	-0.557	+0.557
Bkgr. $_{IRF}$ [Cnts]	-1.19	-1.73	+1.73
Shift $_{IRF}$ [ns]	0.00542	-0.00167	+0.00167

Figure S23: Left: Time-resolved luminescence decay of **23** in acetonitrile including the residuals and IRF (λ_{exc} = 376.7 nm). Right: Fitting parameters including pre-exponential factors and confidence limits.

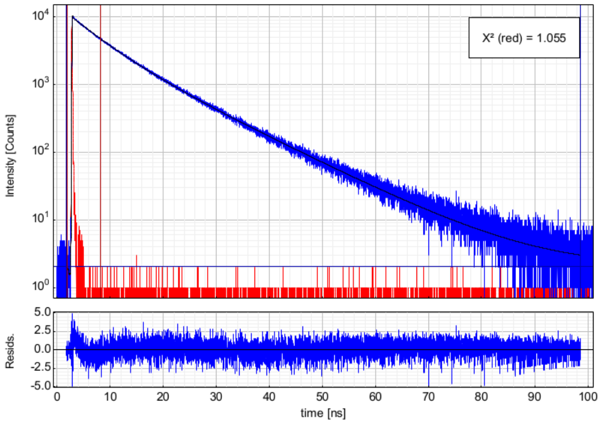

Parameter	Value	Conf. $_{Lower}$	Conf. $_{Upper}$
A_1 [Cnts]	4256.4	-38.6	+38.6
τ_1 [ns]	11.4191	-0.0631	+0.0631
A_2 [Cnts]	5668.3	-78.5	+78.5
τ_2 [ns]	5.3655	-0.0702	+0.0702
A_3 [Cnts]	4557	-984	+984
τ_3 [ns]	0.0863	-0.0228	+0.0228
Bkgr. $_{Dec}$ [Cnts]	2.063	-0.694	+0.694
Bkgr. $_{IRF}$ [Cnts]	0.200	-1.01	+1.01
Shift $_{IRF}$ [ns]	0.00546	-0.00319	+0.00319

Figure S24: Left: Time-resolved luminescence decay of **23** in a frozen butyronitrile glassy matrix at 77 K including the residuals and IRF (λ_{exc} = 376.7 nm). Right: Fitting parameters including pre-exponential factors and confidence limits.

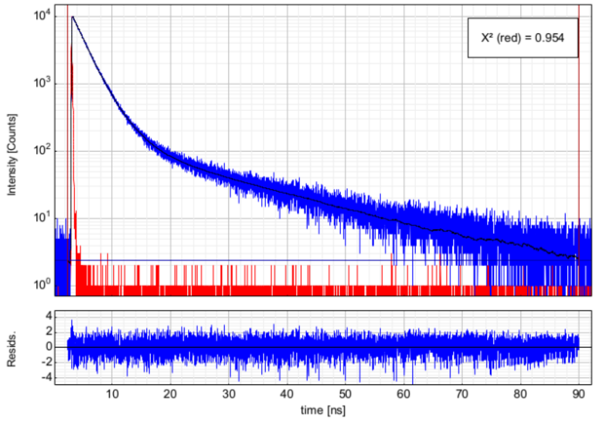

Parameter	Value	Conf. $_{Lower}$	Conf. $_{Upper}$
A_1 [Cnts]	169.22	-6.59	+6.59
τ_1 [ns]	17.912	-0.431	+0.431
A_2 [Cnts]	2497.5	-71.6	+71.6
τ_2 [ns]	3.2709	-0.0633	+0.0633
A_3 [Cnts]	8389	-115	+115
τ_3 [ns]	2.0108	-0.0237	+0.0237
Bkgr. $_{Dec}$ [Cnts]	2.436	-0.554	+0.554
Bkgr. $_{IRF}$ [Cnts]	0.1591	-0.0295	+0.0295
Shift $_{IRF}$ [ns]	0.06577	-0.00342	+0.00342

Figure S25: Left: Time-resolved luminescence decay of **24** in a frozen butyronitrile glassy matrix at 77 K including the residuals and IRF (λ_{exc} = 376.7 nm). Right: Fitting parameters including pre-exponential factors and confidence limits.

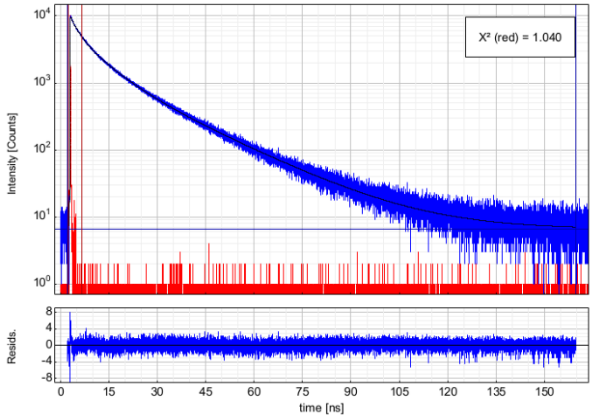

Parameter	Value	Conf. $_{Lower}$	Conf. $_{Upper}$
A_1 [Cnts]	1966.8	-27.3	+27.3
τ_1 [ns]	18.920	-0.153	+0.153
A_2 [Cnts]	4310.1	-73.6	+73.6
τ_2 [ns]	7.213	-0.110	+0.110
A_3 [Cnts]	4212	-179	+179
τ_3 [ns]	2.086	-0.102	+0.102
Bkgr. $_{Dec}$ [Cnts]	6.577	-0.792	+0.792
Bkgr. $_{IRF}$ [Cnts]	-1.80	-2.56	+2.56
Shift $_{IRF}$ [ns]	0.13503	-0.00459	+0.00459

Figure S26: Left: Time-resolved luminescence decay of **24*** in a frozen butyronitrile glassy matrix at 77 K including the residuals and IRF (λ_{exc} = 376.7 nm). Right: Fitting parameters including pre-exponential factors and confidence limits.

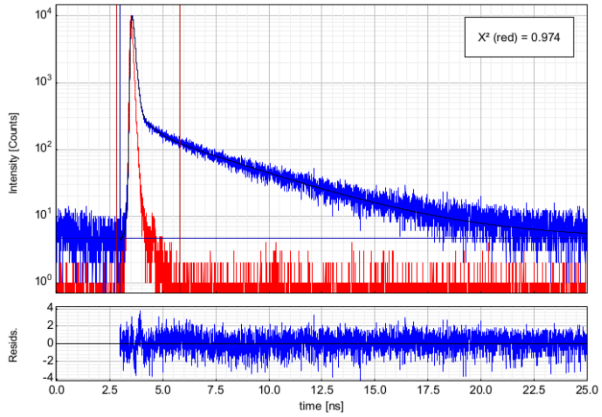

Parameter	Value	Conf. $_{Lower}$	Conf. $_{Upper}$
A_1 [Cnts]	204.31	-6.14	+6.14
τ_1 [ns]	3.9618	-0.0937	+0.0937
A_2 [Cnts]	30357	-512	+512
τ_2 [ns]	0.06931	-0.00103	+0.00103
A_3 [Cnts]	286.0	-44.8	+44.8
τ_3 [ns]	0.5265	-0.0624	+0.0624
Bkgr. $_{Dec}$ [Cnts]	4.618	-0.639	+0.639
Bkgr. $_{IRF}$ [Cnts]	0.473	-1.97	+1.97
Shift $_{IRF}$ [ns]	-0.03163	-0.00133	+0.00133

Figure S27: Left: Time-resolved luminescence decay of **25** in acetonitrile including the residuals and IRF (λ_{exc} = 376.7 nm). Right: Fitting parameters including pre-exponential factors and confidence limits.

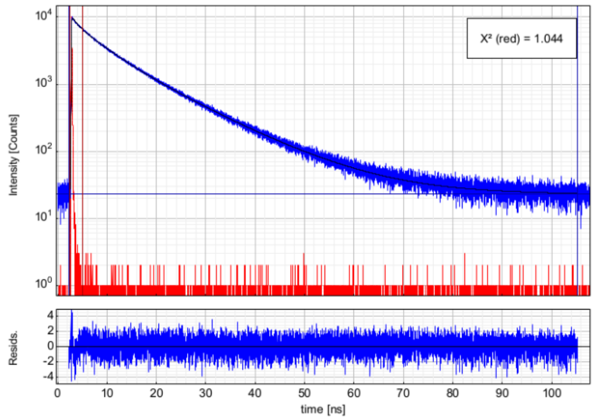

Parameter	Value	Conf. Lower	Conf. Upper
A_1 [Cnts]	3973.7	-37.1	+37.1
τ_1 [ns]	11.9494	-0.0728	+0.0728
A_2 [Cnts]	4644.3	-77.1	+77.1
τ_2 [ns]	5.4108	-0.0864	+0.0864
A_3 [Cnts]	1948	-221	+221
τ_3 [ns]	1.061	-0.150	+0.150
Bkgr. Dec [Cnts]	22.88	-1.26	+1.26
Bkgr. IRF [Cnts]	-3.21	-3.11	+3.11
Shift IRF [ns]	0.23633	-0.00360	+0.00360
A Scat [Cnts]	31540	-6660	+6660

Figure S28: Left: Time-resolved luminescence decay of **25** in a frozen butyronitrile glassy matrix at 77 K including the residuals and IRF (λ_{exc} = 376.7 nm). Right: Fitting parameters including pre-exponential factors and confidence limits.

5 Calculation of absorption and emission spectra

All single point DFT calculations were performed with the Gaussian 09 package[9] using the PBE0 hybrid exchange-correlation functional[10] and the 6-31G* basis set. Absorption spectra were obtained from TDDFT calculations at the optimized ground state geometries, emission spectra from TDDFT calculations at the optimized S_1 geometry. The acetonitrile solvent was taken into account through a polarizable continuum model with default (UFF) atomic radii. For each of the species **16**, **19**, and **24**, dynamically broadened emission spectra were calculated by averaging over the spectra of 10 geometries sampled randomly from a Car-Parrinello molecular dynamics (CP-MD) simulation in the S_1 state. The AIMD runs were carried out with the CPMD code[11] using a fictitious orbital mass of 400 a.u. and a time step of 4 a.u. (ca. 0.1 fs). The mean temperature was set to 900 K and controlled by a Nosé-Hoover chain thermostat.[12-14] The elevated temperature was chosen to account for the difference in potential energy between the Franck-Condon point and the S_1 minimum. The S_1 state was descibed using Restricted Open-Shell Kohn-Sham theory, a plane wave basis set truncated at 70 Ry, and normconserving (Troullier-Martins) pseudopotentials.

16	17	18	19
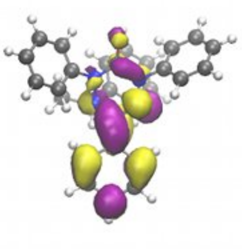	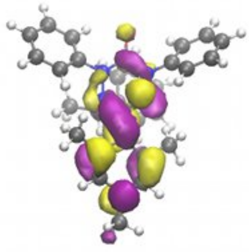	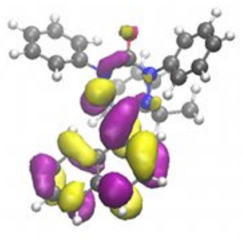	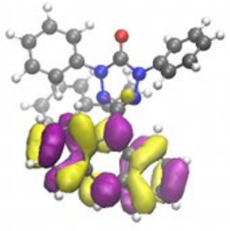
**-0.04925 a.u.<br>= -1.340 eV**	**-0.03898 a.u.<br>= -1.061 eV**	**-0.06046 a.u.<br>= -1.645 eV**	**-0.07460 a.u.<br>= -2.030 eV**
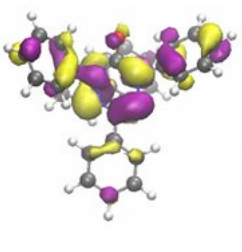	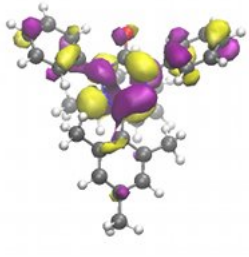	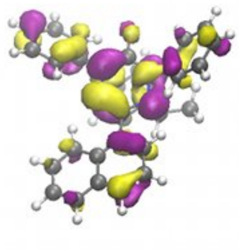	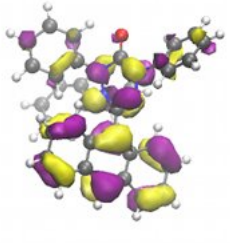
**-0.21498 a.u.<br>= -5.850 eV**	**-0.20951 a.u.<br>= -5.701 eV**	**-<br>-0.21333 a.u.<br>= -5.805 eV**	**-0.21095 a.u.<br>= -5.740 eV**

20	21	22	23
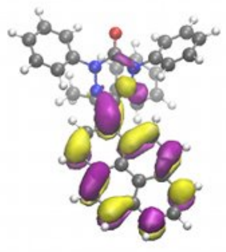	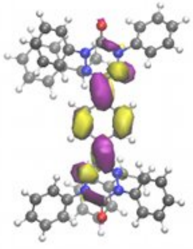	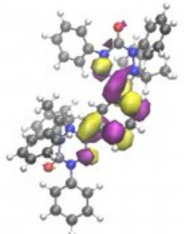	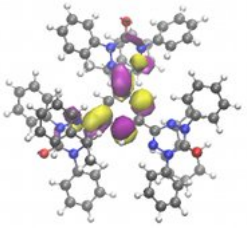
**-0.07436 a.u. = -2.023 eV**	**-0.07232 a.u. = -1.968 eV**	**-0.06110 a.u. = -1.663 eV**	**-0.06706 a.u. = -1.825 eV**
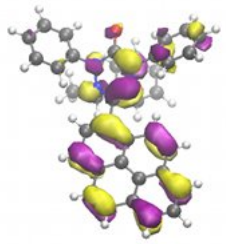	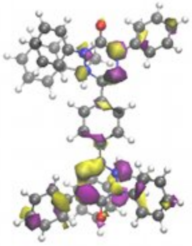	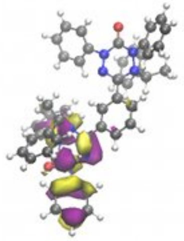	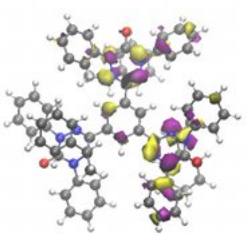
**-0.20797 a.u. = -5.659 eV**	**-0.21410 a.u. = -5.826 eV**	**-0.21672 a.u. = -5.897 eV**	**-0.21756 a.u. = -5.920 eV**

Figure S29: Visualisation of calculated HOMOs and LUMOs of species **16 – 23** and their corresponding orbital energies.

Table 2 contains geometric data of the ground state optimized structures in the gas phase together with the experimental X-ray data. The torsion angles between the central heterocycle and the R-substituent are defined as the NCCC dihedral angles and the corresponding C-C bond is listed. The torsion angles of the heterocycle are defined as the CNNC dihedral angles as a measure for the asymmetry within the ring.

Table S2: Geometric data from DFT/6-31G* calculations in the gas phase and comparison with experimental data obtained from X-Ray crystallography.

**Species**	**NCCC dihedral angle [deg]**		**CC bond length [Ang]**		**CNNC dihedral angle [deg]**	
	**theor.**	**exp.**	**theor.**	**exp.**	**theor.**	**exp.**
8	8.8 / 8.8	12.3 / 12.3 [7]	1.480	1.476 [7]	3.1 / 3.0	1.9 / 1.9 [7]
9	62.4 / 62.2		1.488		4.9 / 4.9	
10	42.3 / 45.9	45.6(2) / 47.2(2)	1.484	1.487(2)	-3.9 / 2.3	-10.1(2) / -2.0(2)
11	64.2 / 63.9	87.9(5) / 86.4(5)	1.489	1.490(5)	0.4 / 5.1	0.4(5) / -4.3(5)
16	16.6 / 16.2		1.472		40.4 / 30.6	
17	47.2 / 51.4		1.487		33.5 / 27.5	

18	28.2 / 25.8	17.0 / 14.2	1.477	1.481(2)	-30.9 / -39.6	-35.5 / -39.9
19	-63.3 / -64.2	-67.4 / -69.9	1.485	1.490(2)	45.1 / 29.5	37.8 / 33.1
20	27.1 / 29.5		1.475		39.2 / 31.2	
21	14.4 / 15.0		1.470		42.2 / 31.8	
22	13.0 / 13.5<br>20.7 / 20.2		1.472<br>1.472		41.3 / 31.1<br>25.3 / 41.1	
23	9.10 / 10.09<br>13.75 / 13.37<br>24.2 / 24.2		1.472<br>1.472<br>1.472		41.6 / 31.2<br>40.5 / 30.7<br>41.6 / 24.5	
24	20.9 / 21.3		1.472		35.4 / 33.0	

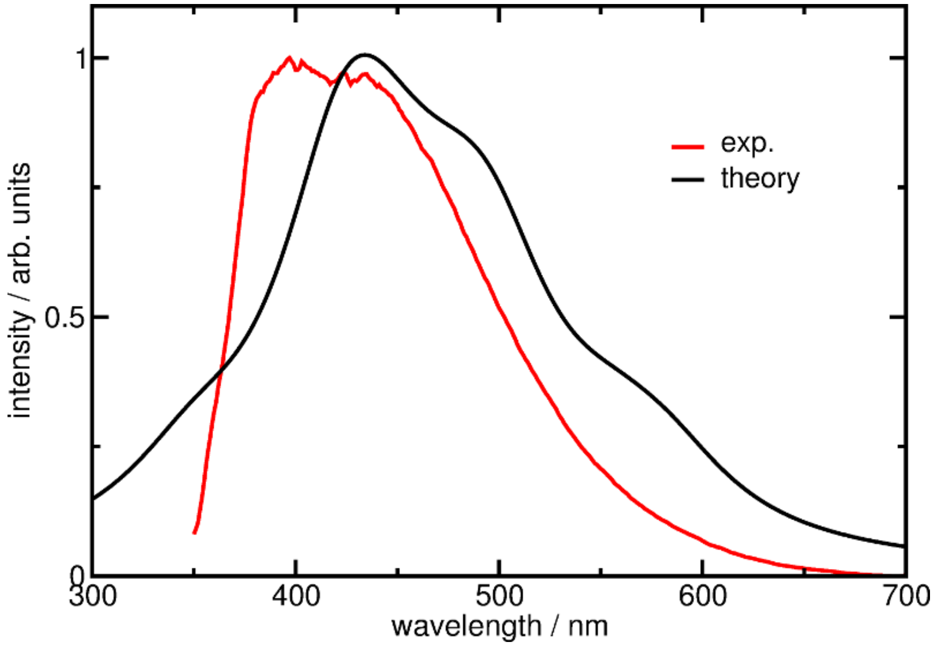

Figure S30: Comparison of theoretical and experimental emission spectra of compound **16** in acetonitrile. The theoretical spectrum was obtained by averaging over 10 TDDFT/6-31-G*/PCM calculations for geometries sampled from a CP-MD run in the S_1 state. The temperature had to be reduced to 300 K for this molecule not to dissociate.

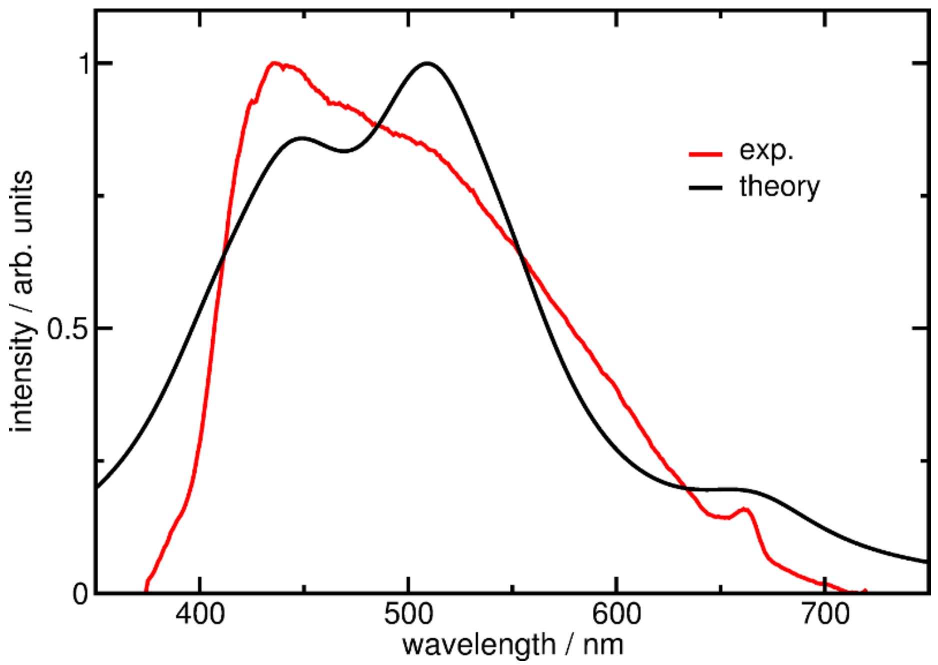

Figure S31: Comparison of theoretical and experimental emission spectra of compound **19** in acetonitrile. The theoretical spectrum was obtained by averaging over 10 TDDFT/6-31-G*/PCM calculations for geometries sampled from a CP-MD run in the S_1 state.

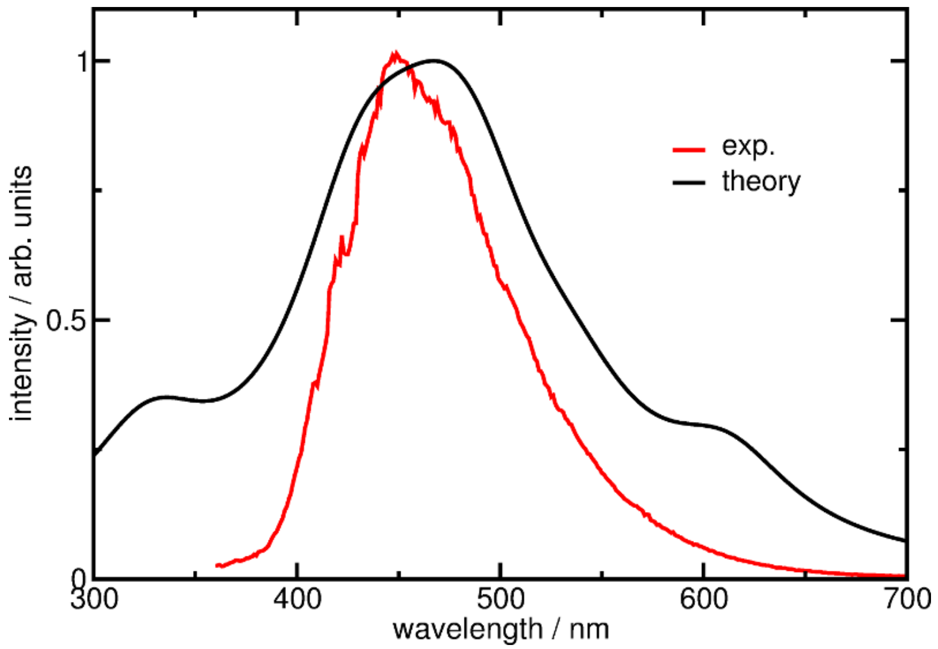

Figure S32: Comparison of theoretical and experimental emission spectra of compound **24** in acetonitrile. The theoretical spectrum was obtained by averaging over 10 TDDFT/6-31-G*/PCM calculations for geometries sampled from a CP-MD run in the S_1 state at 500 K, while the experimental spectrum was recorded at 77 K. The contributions at long wavelengths in the theoretical spectrum indicate a high probability for nonradiative decay.

6 X-Ray crystallographic data

X-Ray diffraction: Data sets were collected with a Nonius KappaCCD diffractometer. Programs used: data collection, COLLECT (R. W. W. Hooft, Bruker AXS, 2008, Delft, The Netherlands); data reduction Denzo-SMN;[15] absorption correction, Denzo;[16] structure solution SHELXS-97;[17] structure refinement SHELXL-97[18] and graphics, XP (Bruker AXS, 2000). Thermals ellipsoids are shown with 30% probability, *R*-values are given for observed reflections, and wR^2 values are given for all reflections.

X-ray crystal structure analysis of 10: formula $C_{24}H_{17}N_4O$, M = 377.42, red crystal, 0.40 x 0.07 x 0.05 mm, a = 3.9114(4), b = 20.5945(6), c = 22.4580(10) Å, β = 91.758(6) °, V = 1808.2(2) Å^3, ρ_{calc} = 1.386 gcm^{-3}, μ = 0.700 mm^{-1}, empirical absorption correction (0.767 ≤ T ≤ 0.965), Z = 4, monoclinic, space group $P2_1/n$ (No. 14), λ = 1.54178 Å, T = 223(2) K, ω and φ scans, 22085 reflections collected (±*h*, ±*k*, ±*l*), [(sinθ)/λ] = 0.60 Å^{-1}, 3192 independent (R_{int} = 0.044) and 2887 observed reflections [I>2σ(I)], 262 refined parameters, R = 0.037, wR^2 = 0.100, max. (min.) residual electron density 0.14 (-0.20) e.Å^{-3}, hydrogen atoms calculated and refined as riding atoms.

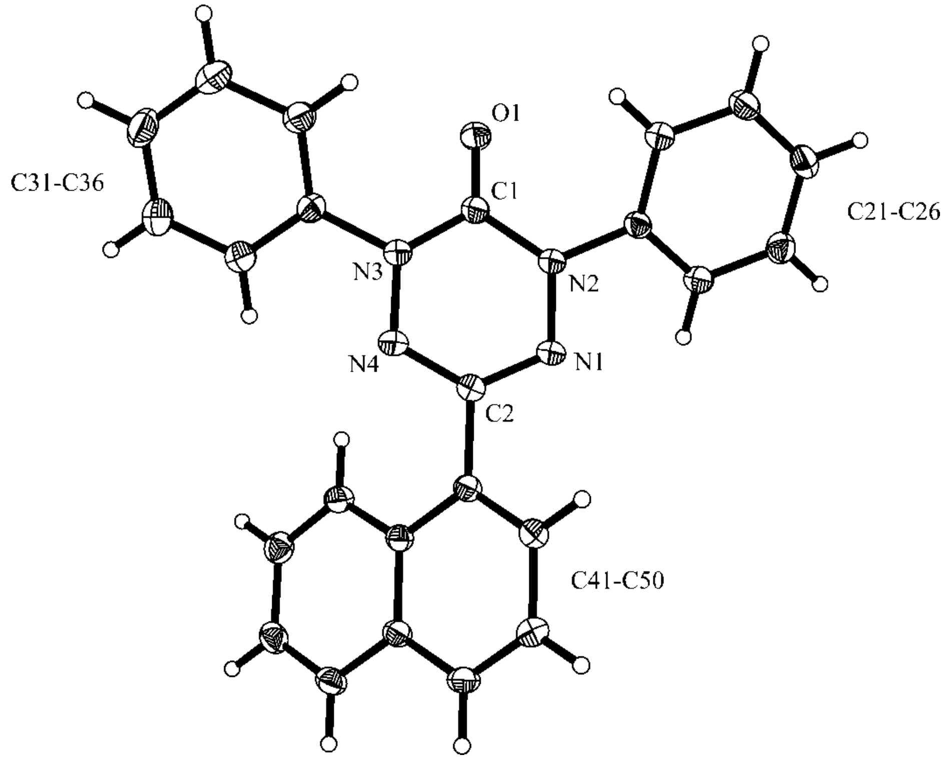

Figure S33: X-ray crystal structure analysis of **10**.
(Thermals ellipsoids are shown with 30% probability)

X-ray crystal structure analysis of 11: formula $C_{28}H_{19}N_4O$, *M* = 427.47, red crystal, 0.55 x 0.04 x 0.02 mm, *a* = 11.7763(13), *b* = 5.8280(3), *c* = 16.1543(9) Å, *β* = 104.773(6) °, *V* = 1072.0(1) Å^3, ρ_{calc} = 1.324 gcm^{-3}, μ = 0.658 mm^{-1}, empirical absorption correction (0.713 ≤ T ≤ 0.987), *Z* = 2, monoclinic, space group $P2_1$ (No. 4), λ = 1.54178 Å, *T* = 223(2) K, ω and φ scans, 10206 reflections collected (±*h*, ±*k*, ±*l*), [(sinθ)/λ] = 0.60 Å^{-1}, 3257 independent (R_{int} = 0.080) and 2635 observed reflections [$I > 2\sigma(I)$], 299 refined parameters, *R* = 0.057, wR^2 = 0.168, max. (min.) residual electron density 0.17 (-0.14) e.Å^{-3}, hydrogen atoms calculated and refined as riding atoms. Flack parameter: -0.4(6).

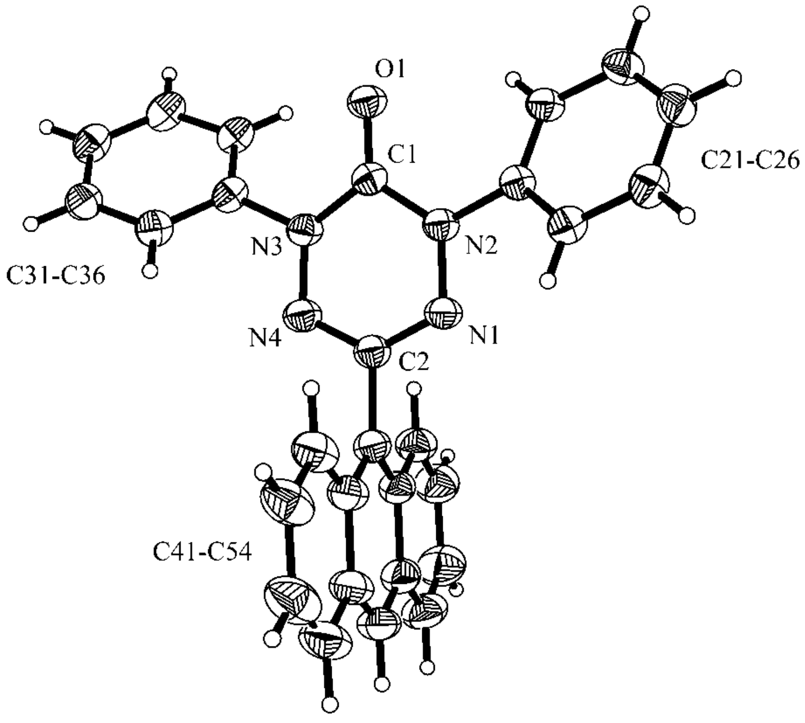

Figure S34: X-ray crystal structure analysis of **11**.
(Thermals ellipsoids are shown with 30% probability)

X-ray crystal structure analysis of 18: formula $C_{32}H_{26}N_4O$, *M* = 482.57, pale yellow crystal, 0.22 x 0.10 x 0.07 mm, *a* = 7.4740(8), *b* = 9.2641(5), *c* = 35.8650(18) Å, *β* = 91.227(5) °, *V* = 2482.7(3) Å^3, ρ_{calc} = 1.291 gcm^{-3}, μ = 0.627 mm^{-1}, empirical absorption correction (0.874 ≤ T ≤ 0.957), *Z* = 4, monoclinic, space group $P2_1/c$ (No. 14), λ = 1.54178 Å, *T* = 223(2) K, ω and φ scans, 21885 reflections collected (±*h*, ±*k*, ±*l*), [(sinθ)/λ] = 0.60 Å^{-1}, 4280 independent (R_{int} = 0.063) and 3416 observed reflections [$I > 2\sigma(I)$], 335 refined parameters, *R* = 0.041, wR^2 = 0.105, max. (min.) residual electron density 0.13 (-0.20) e.Å^{-3}, hydrogen atoms calculated and refined as riding atoms.

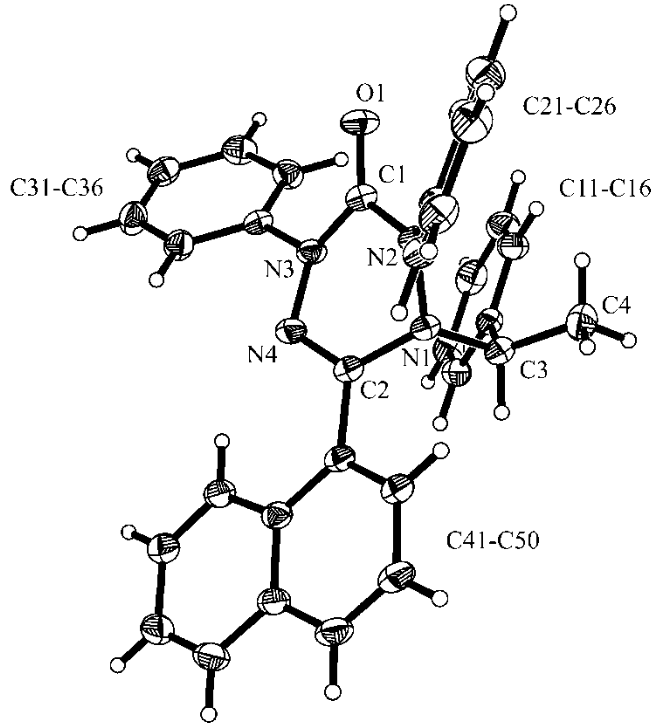

Figure S35: X-ray crystal structure analysis of **18**.
(Thermals ellipsoids are shown with 30% probability)

X-ray crystal structure analysis of 19: formula $C_{36}H_{28}N_4O$, *M* = 532.62, orange crystal, 0.48 x 0.36 x 0.25 mm, *a* = 11.2158(3), *b* = 10.0647(3), *c* = 24.8459(6) Å, *β* = 97.414(2) °, *V* = 2781.2(1) Å^3, ρ_{calc} = 1.272 gcm^{-3}, μ = 0.611 mm^{-1}, empirical absorption correction (0.757 ≤ T ≤ 0.862), *Z* = 4, monoclinic, space group $P2_1/c$ (No. 14), λ = 1.54178 Å, *T* = 223(2) K, ω and φ scans, 20096 reflections collected (±*h*, ±*k*, ±*l*), [(sinθ)/λ] = 0.60 Å^{-1}, 4794 independent (R_{int} = 0.039) and 4370 observed reflections [I>2σ(I)], 371 refined parameters, *R* = 0.047, wR^2 = 0.145, max. (min.) residual electron density 0.29 (-0.21) e.Å^{-3}, hydrogen atoms calculated and refined as riding atoms.

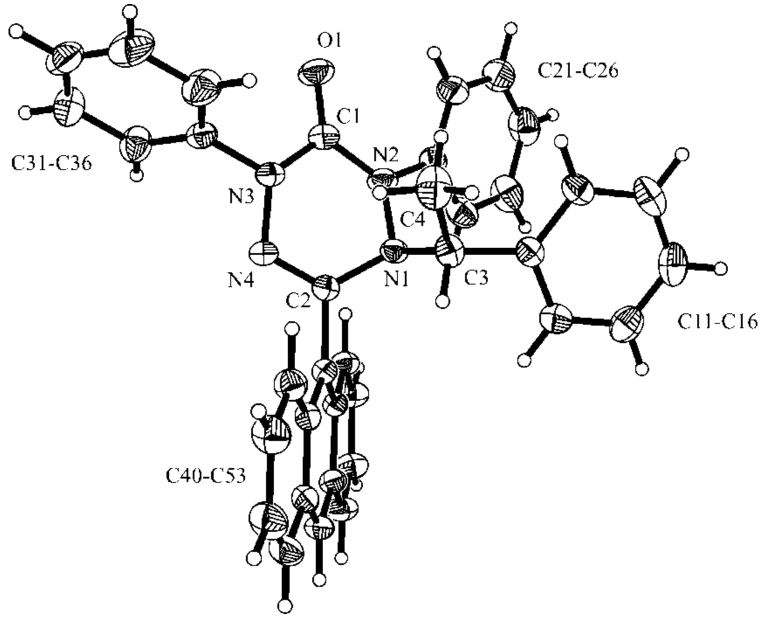

Figure S36: X-ray crystal structure analysis of **19**.
(Thermals ellipsoids are shown with 30% probability)

7 ^{1}H and ^{13}C NMR spectra

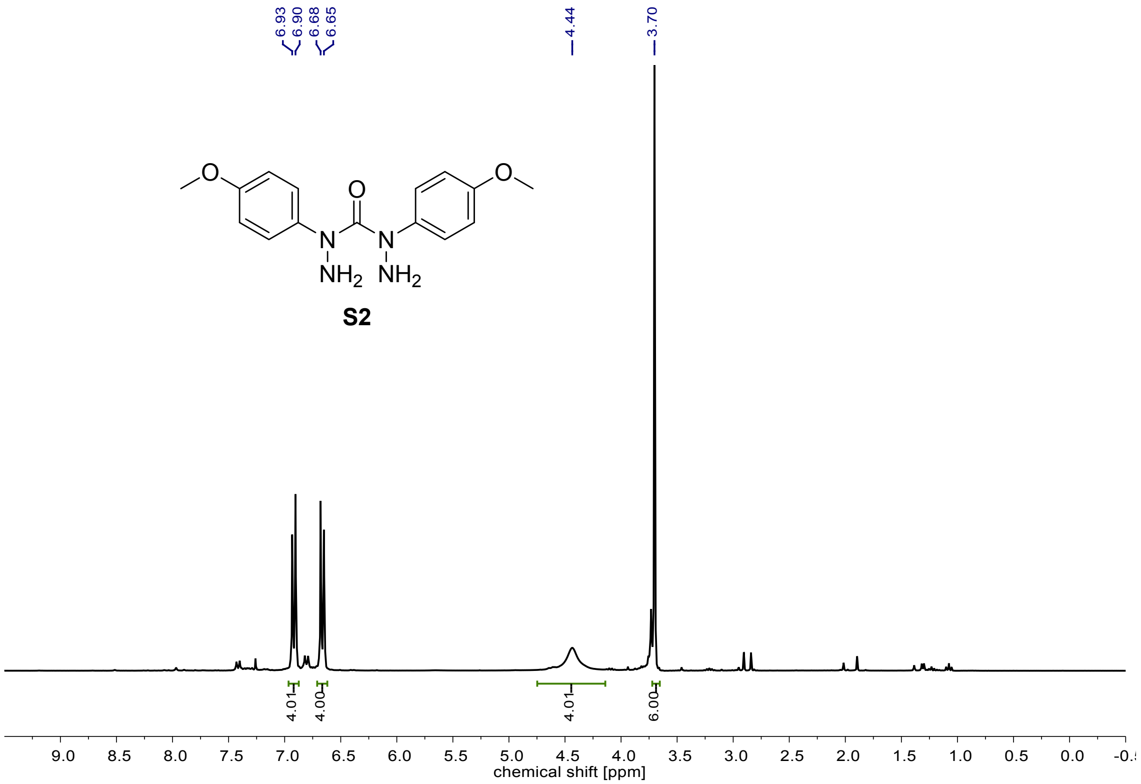

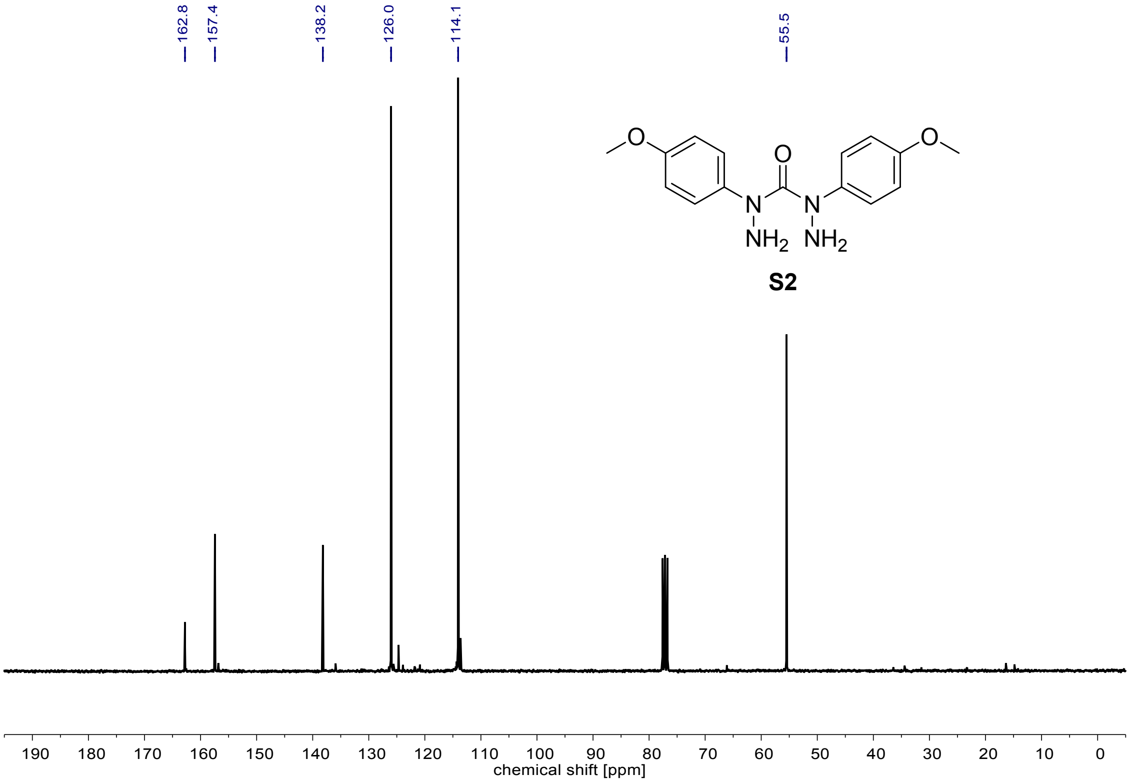

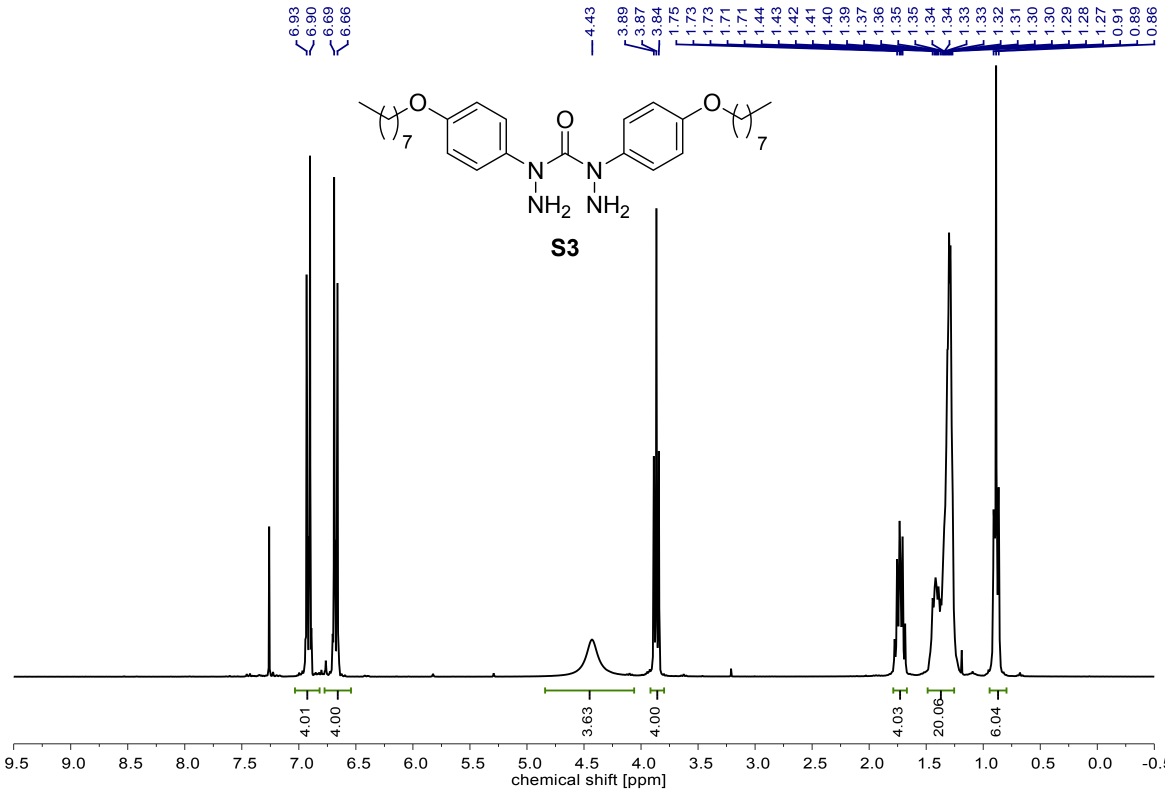
6.93
6.90
6.69
6.66
4.43
3.89
3.87
3.84
1.75
1.73
1.73
1.71
1.71
1.44
1.43
1.42
1.41
1.40
1.39
1.37
1.36
1.35
1.35
1.34
1.34
1.33
1.33
1.32
1.31
1.30
1.30
1.29
1.28
1.27
0.91
0.89
0.86
O
NH_2
NH_2
7
S3
4.01
4.00
3.63
4.00
4.03
20.06
6.04
chemical shift [ppm]

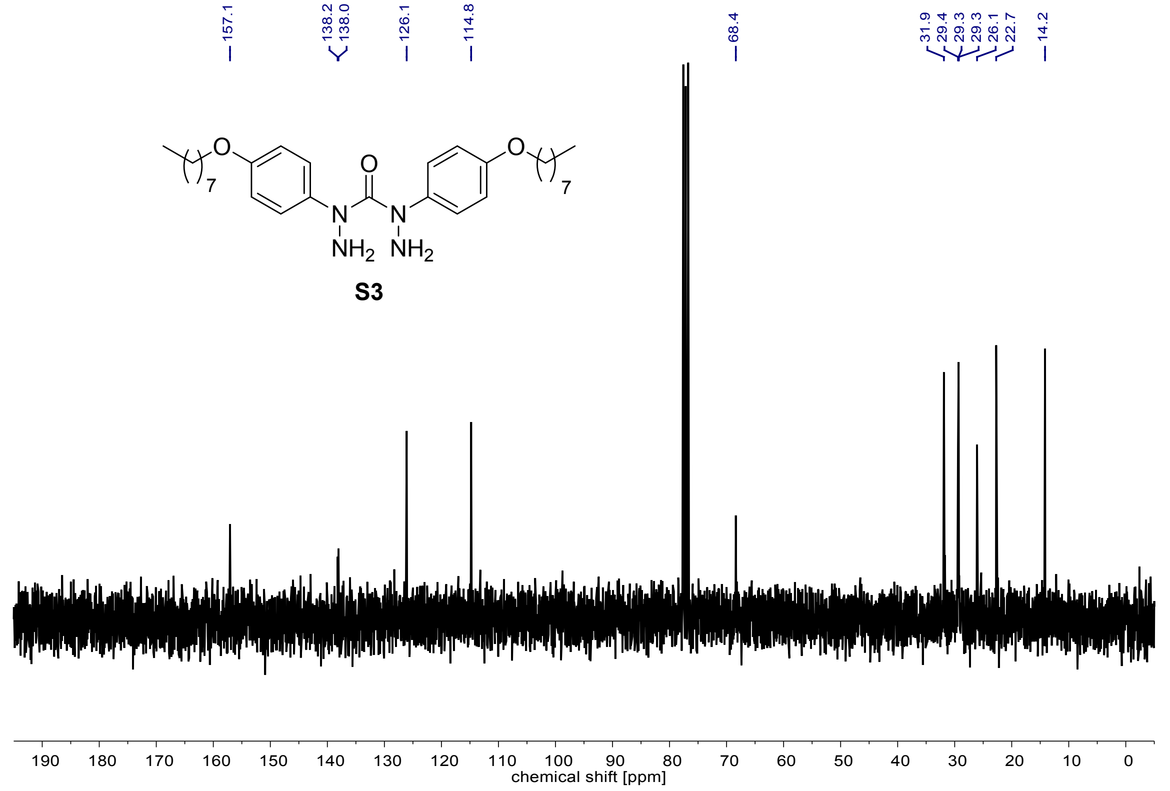
157.1
138.2
138.0
126.1
114.8
68.4
31.9
29.4
29.3
29.3
26.1
22.7
14.2
S3
chemical shift [ppm]

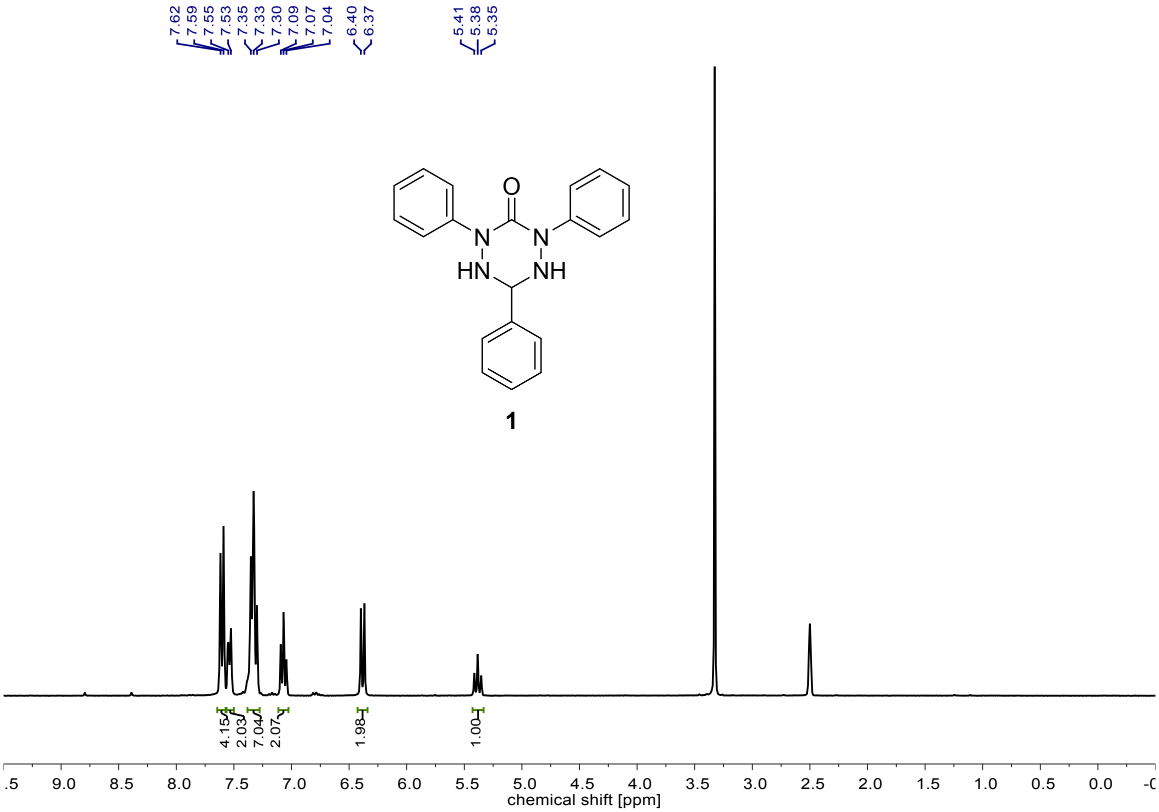
7.62 7.59 7.55 7.53 7.35 7.33 7.30 7.09 7.07 7.04 6.40 6.37 5.41 5.38 5.35
1
4.15 2.03 7.04 2.07 1.98 1.00
chemical shift [ppm]

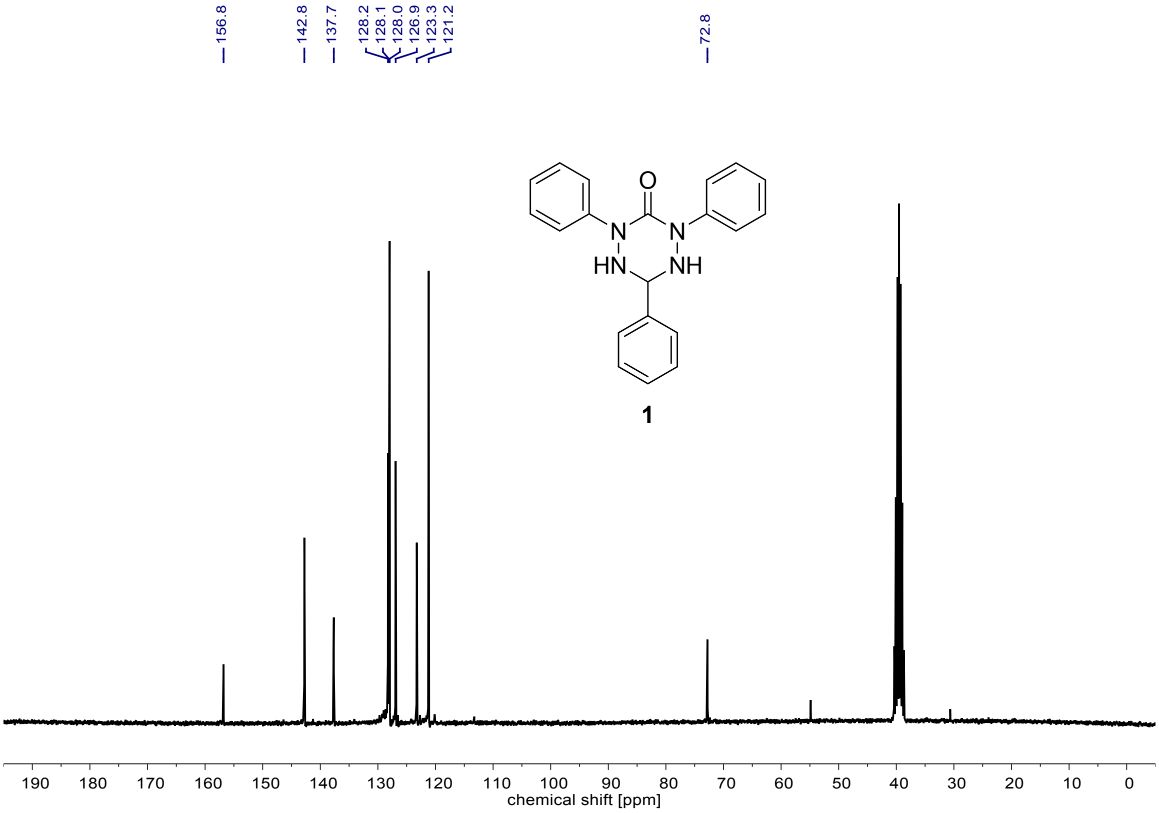
156.8 142.8 137.7 128.2 128.1 128.0 126.9 123.3 121.2 72.8
1
chemical shift [ppm]

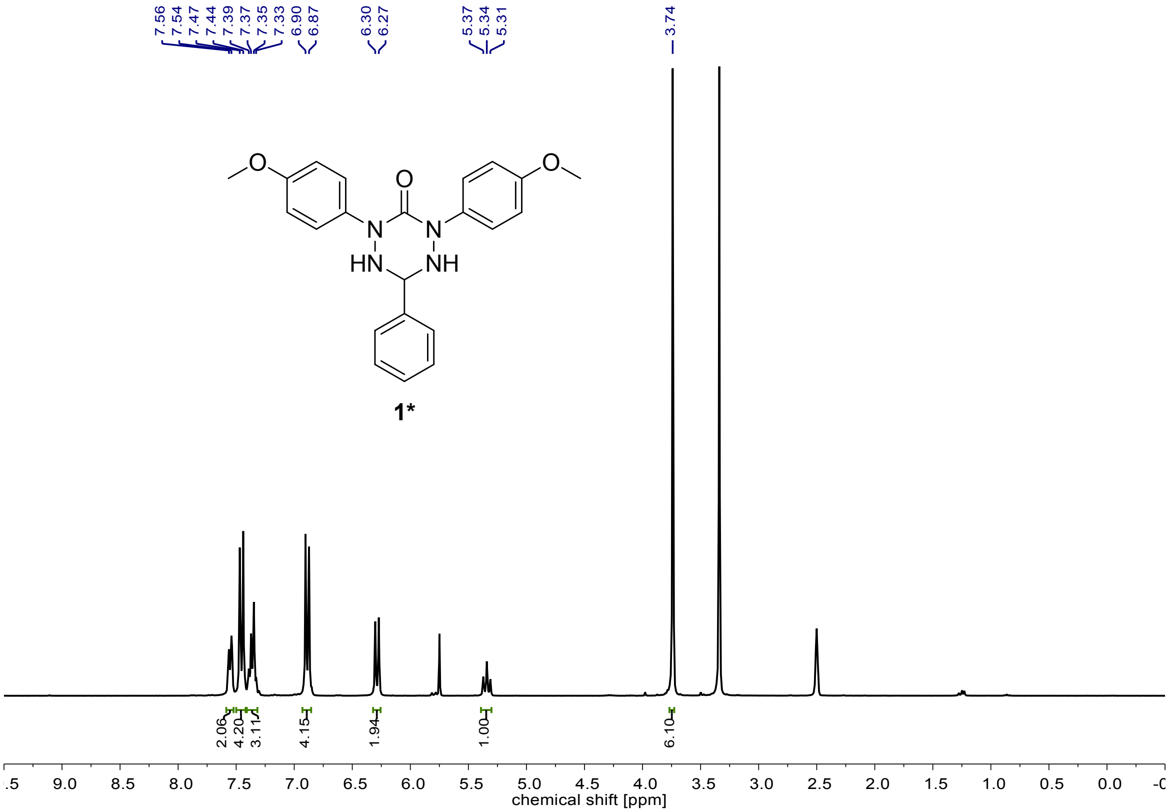
7.56
7.54
7.47
7.44
7.39
7.37
7.35
7.33
6.90
6.87
6.30
6.27
5.37
5.34
5.31
3.74
1*
2.06
4.20
3.11
4.15
1.94
1.00
6.10
chemical shift [ppm]

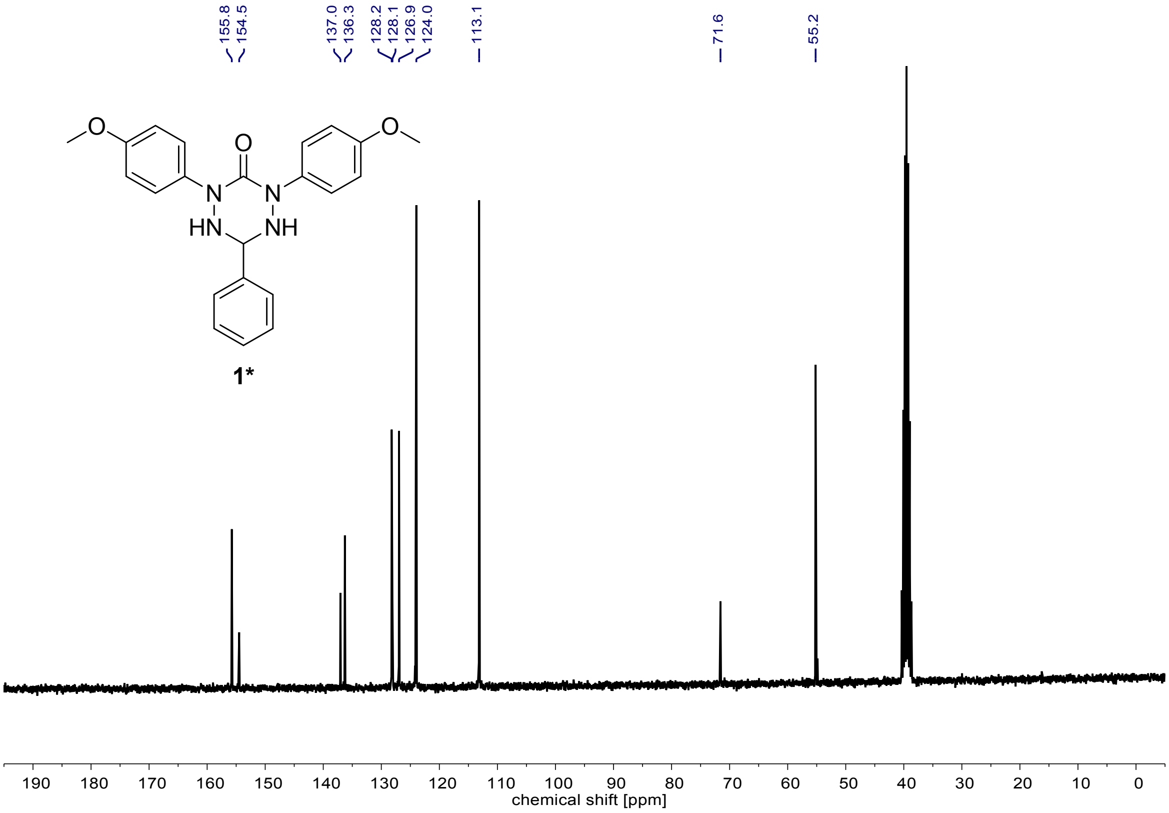
155.8
154.5
137.0
136.3
128.2
128.1
126.9
124.0
113.1
71.6
55.2
1*
chemical shift [ppm]

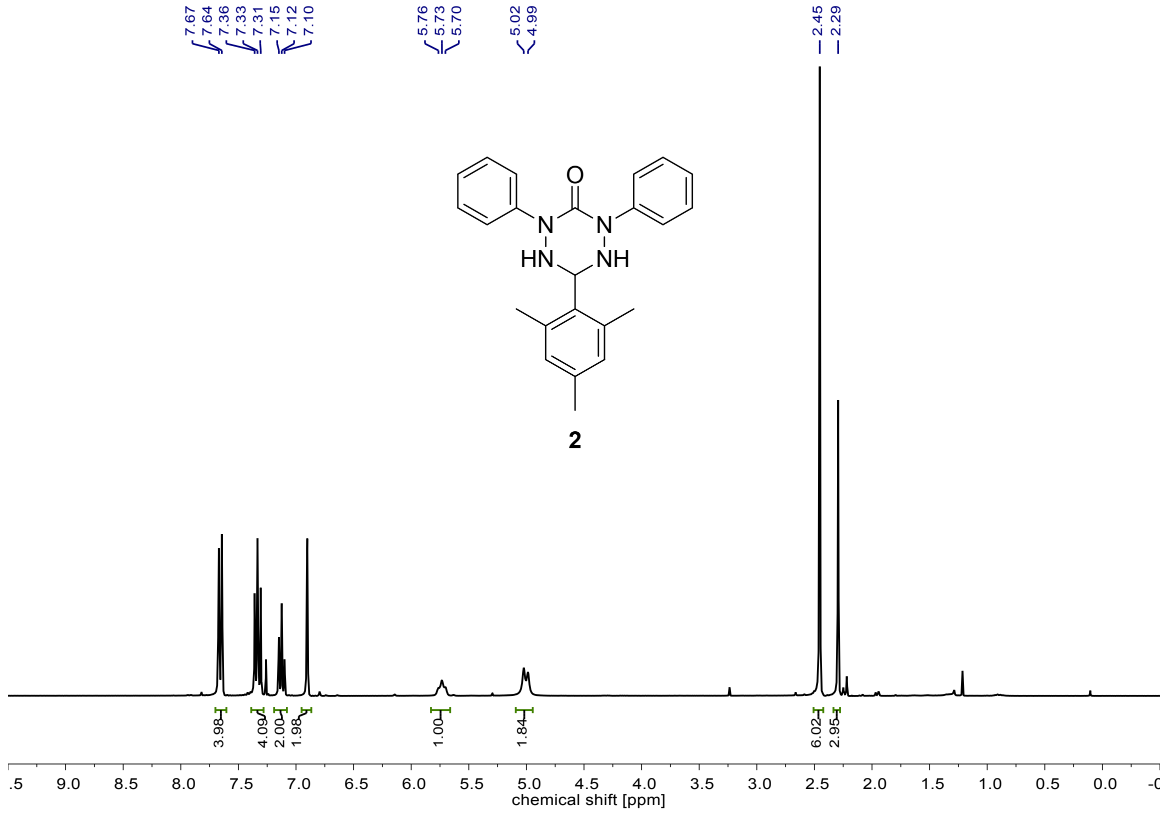
7.67
7.64
7.36
7.33
7.31
7.15
7.12
7.10
5.76
5.73
5.70
5.02
4.99
2.45
2.29
2
3.98
4.09
2.00
1.98
1.00
1.84
6.02
2.95
chemical shift [ppm]

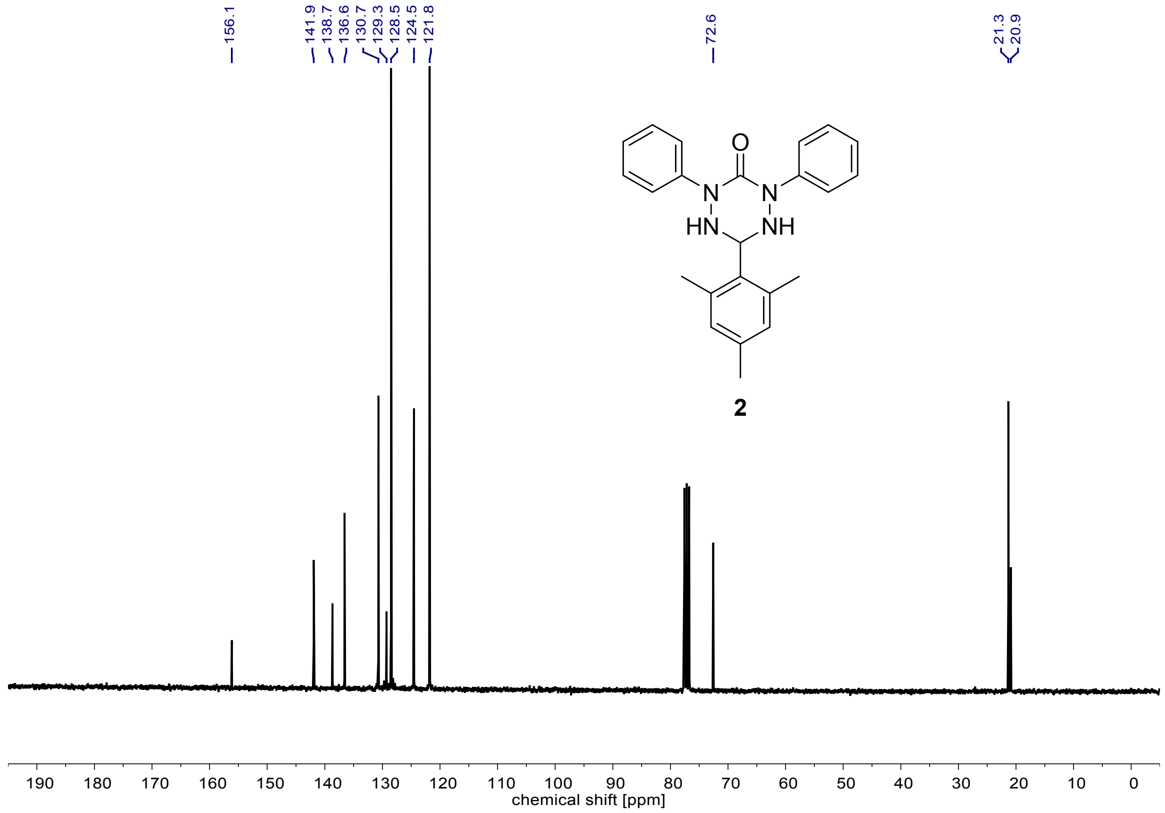
156.1
141.9
138.7
136.6
130.7
129.3
128.5
124.5
121.8
72.6
21.3
20.9
2
chemical shift [ppm]

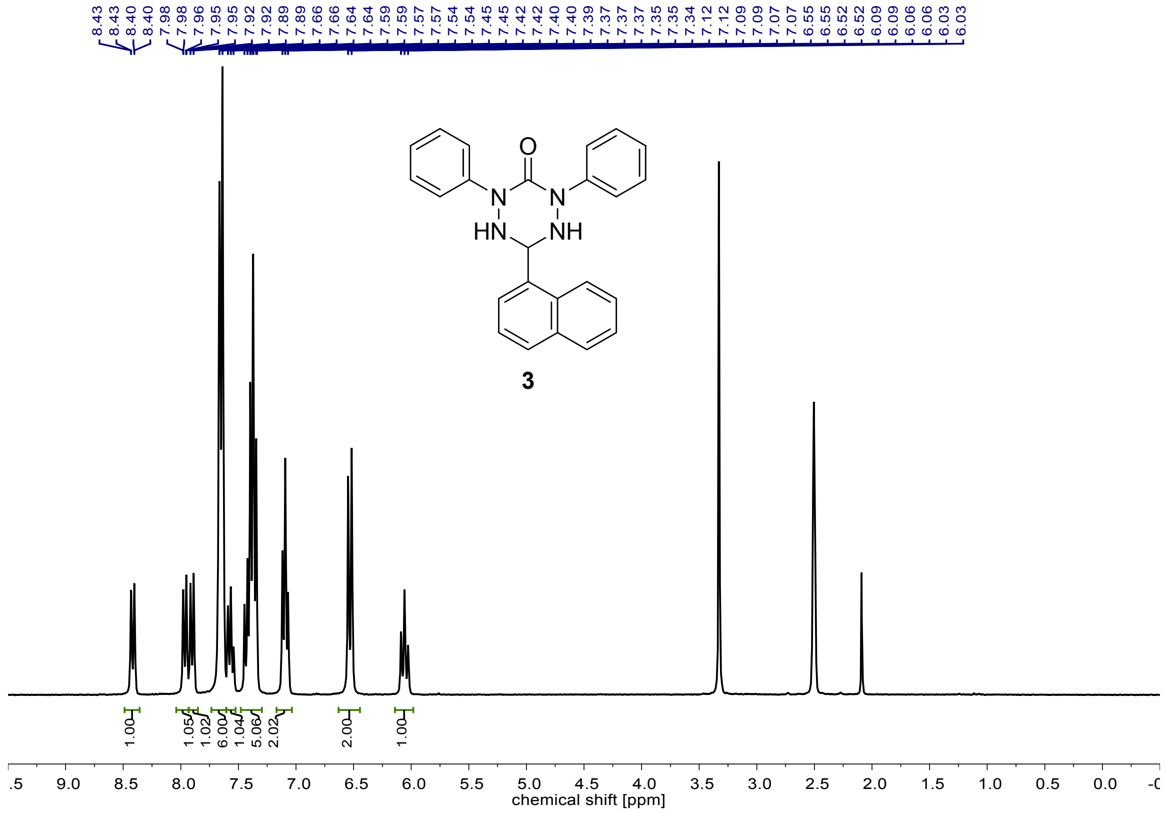
8.43
8.43
8.40
8.40
7.98
7.96
7.95
7.95
7.92
7.92
7.89
7.89
7.66
7.66
7.64
7.64
7.59
7.59
7.57
7.57
7.54
7.45
7.45
7.42
7.42
7.40
7.40
7.39
7.37
7.37
7.37
7.35
7.35
7.34
7.12
7.12
7.09
7.09
7.07
7.07
6.55
6.55
6.52
6.52
6.09
6.09
6.06
6.06
6.03
6.03
O
N
N
HN
NH
3
1.00
1.05
1.02
6.00
1.04
5.06
2.02
2.00
1.00
chemical shift [ppm]

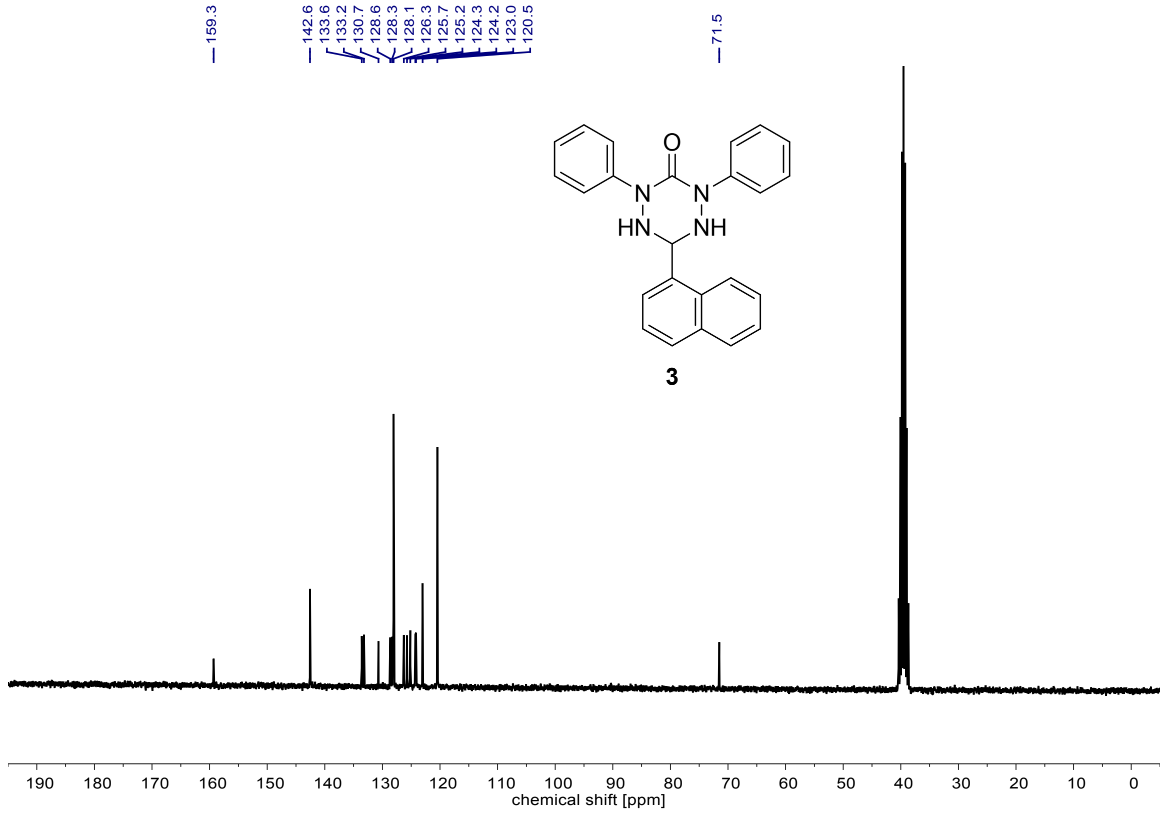
159.3
142.6
133.6
133.2
130.7
128.6
128.3
128.1
126.3
125.7
125.2
124.3
124.2
123.0
120.5
71.5
O
N
N
HN
NH
3
chemical shift [ppm]

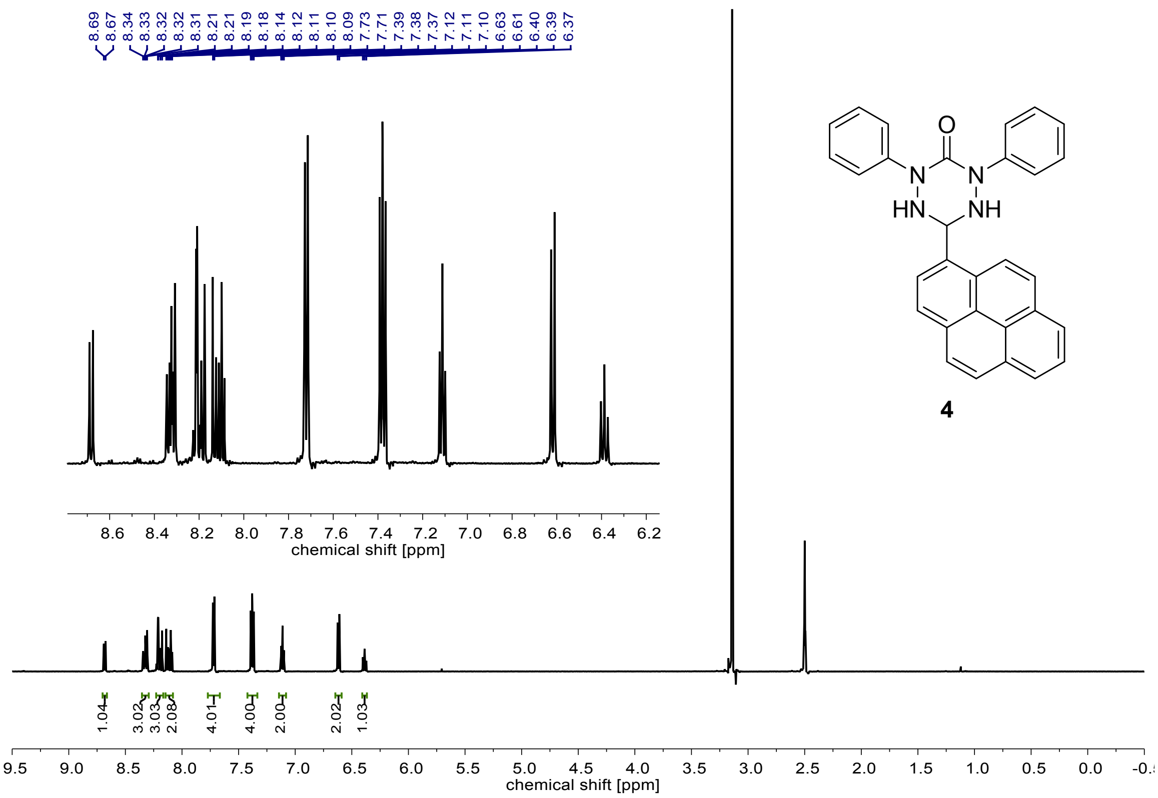
8.69
8.67
8.34
8.33
8.32
8.32
8.31
8.21
8.21
8.19
8.18
8.14
8.12
8.11
8.10
8.09
7.73
7.71
7.39
7.38
7.37
7.12
7.11
7.10
6.63
6.61
6.40
6.39
6.37
HN
NH
N
N
O
4
1.04
3.02
3.03
2.08
4.01
4.00
2.00
2.02
1.03
chemical shift [ppm]
chemical shift [ppm]

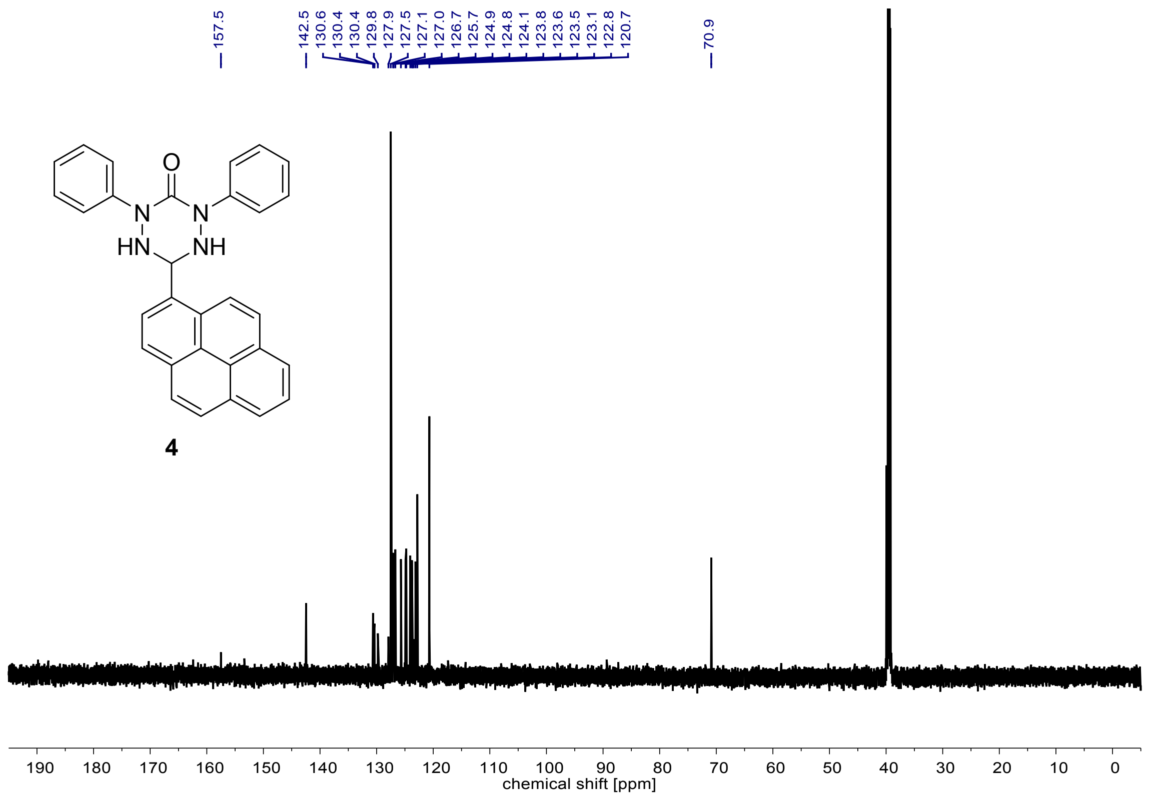
157.5
142.5
130.6
130.4
130.4
129.8
127.9
127.5
127.1
127.0
126.7
125.7
124.9
124.8
124.1
123.8
123.6
123.5
123.1
122.8
120.7
70.9
HN
NH
N
N
O
4
chemical shift [ppm]

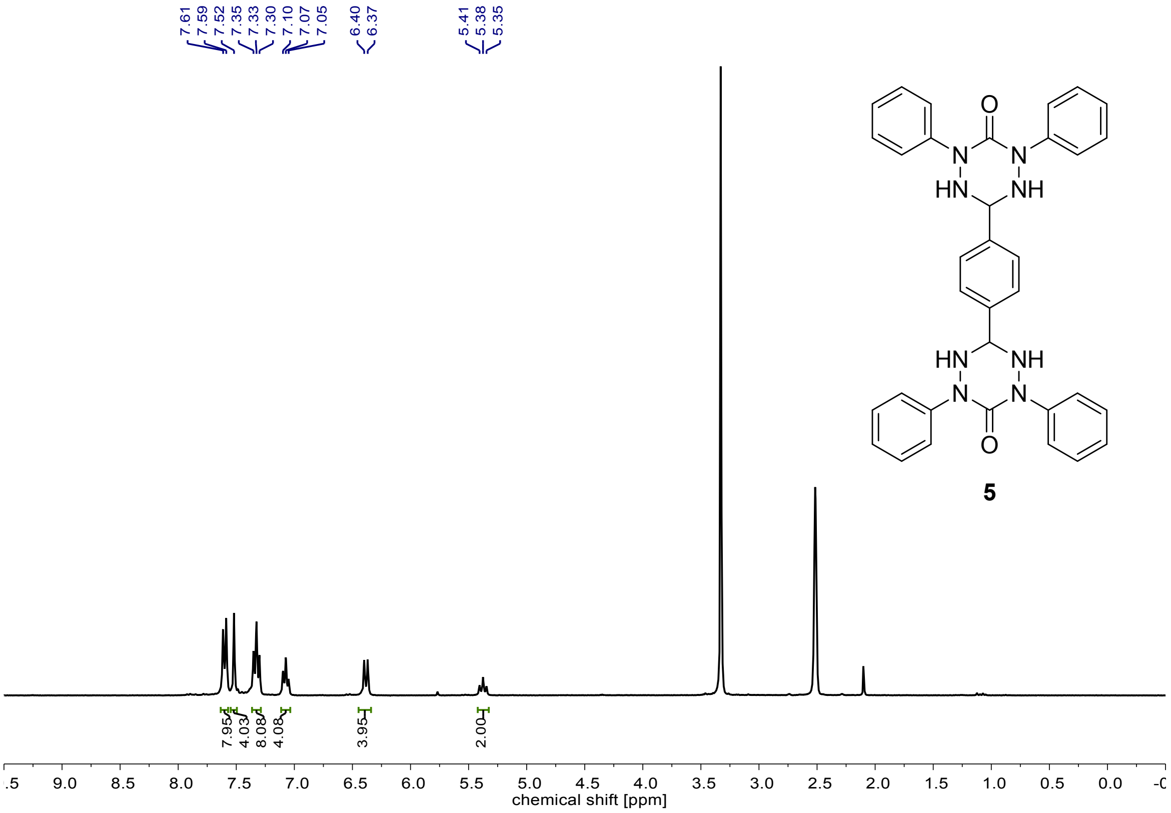
7.61
7.59
7.52
7.35
7.33
7.30
7.10
7.07
7.05
6.40
6.37
5.41
5.38
5.35
7.95
4.03
8.08
4.08
3.95
2.00
5
chemical shift [ppm]

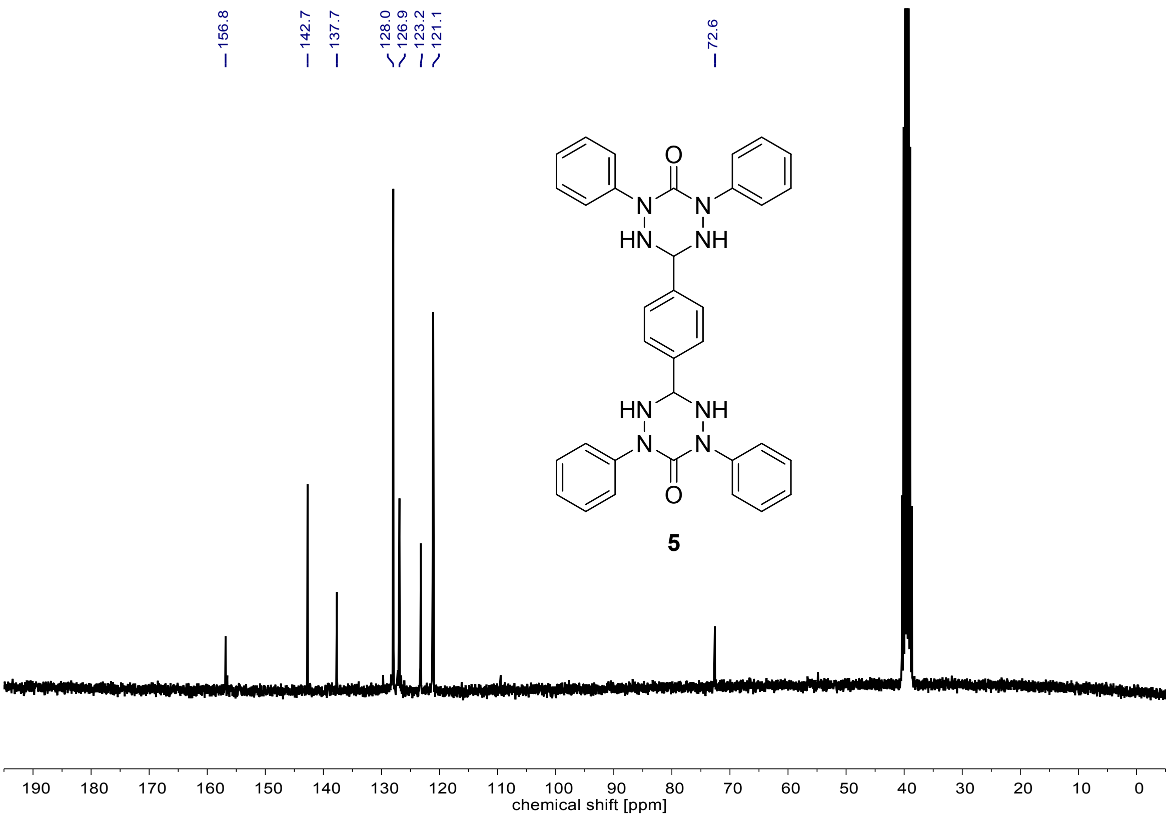
156.8
142.7
137.7
128.0
126.9
123.2
121.1
72.6
5
chemical shift [ppm]

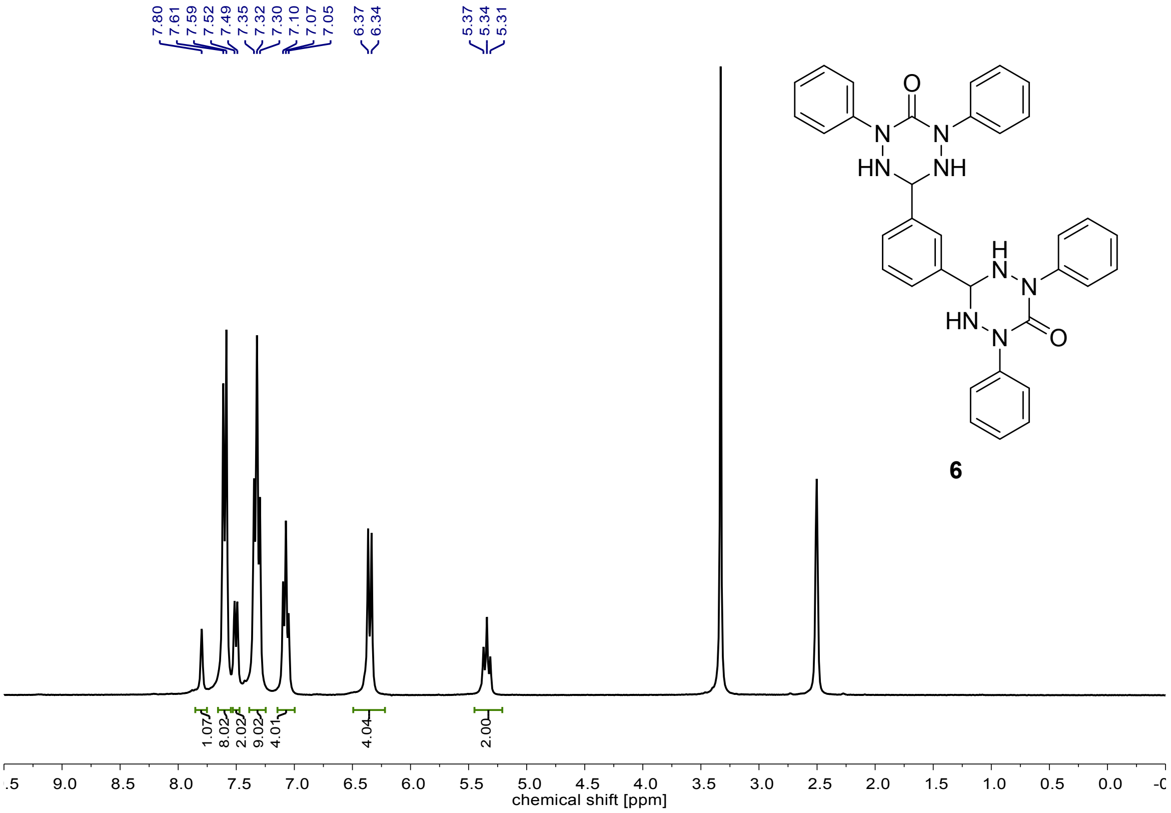
7.80
7.61
7.59
7.52
7.49
7.35
7.32
7.30
7.10
7.07
7.05
6.37
6.34
5.37
5.34
5.31
1.07
8.02
2.02
9.02
4.01
4.04
2.00
chemical shift [ppm]
6

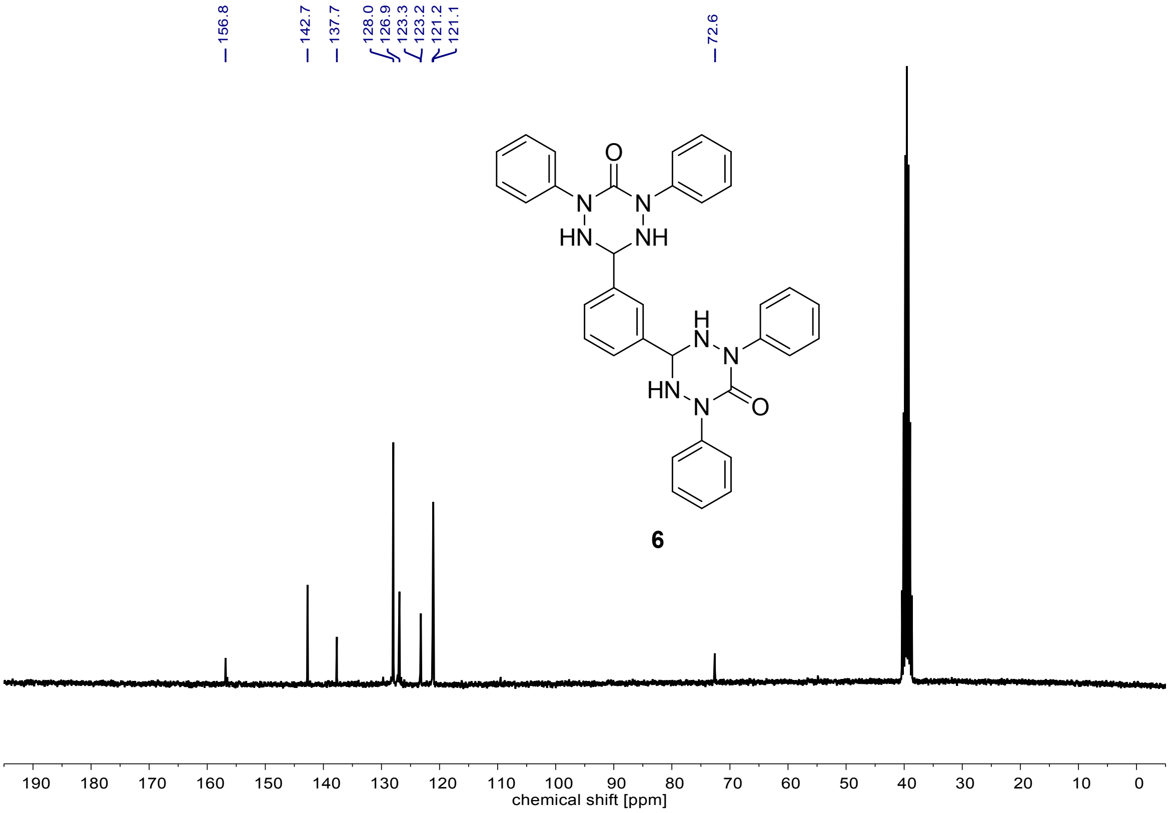
156.8
142.7
137.7
128.0
126.9
123.3
123.2
121.2
121.1
72.6
chemical shift [ppm]
6

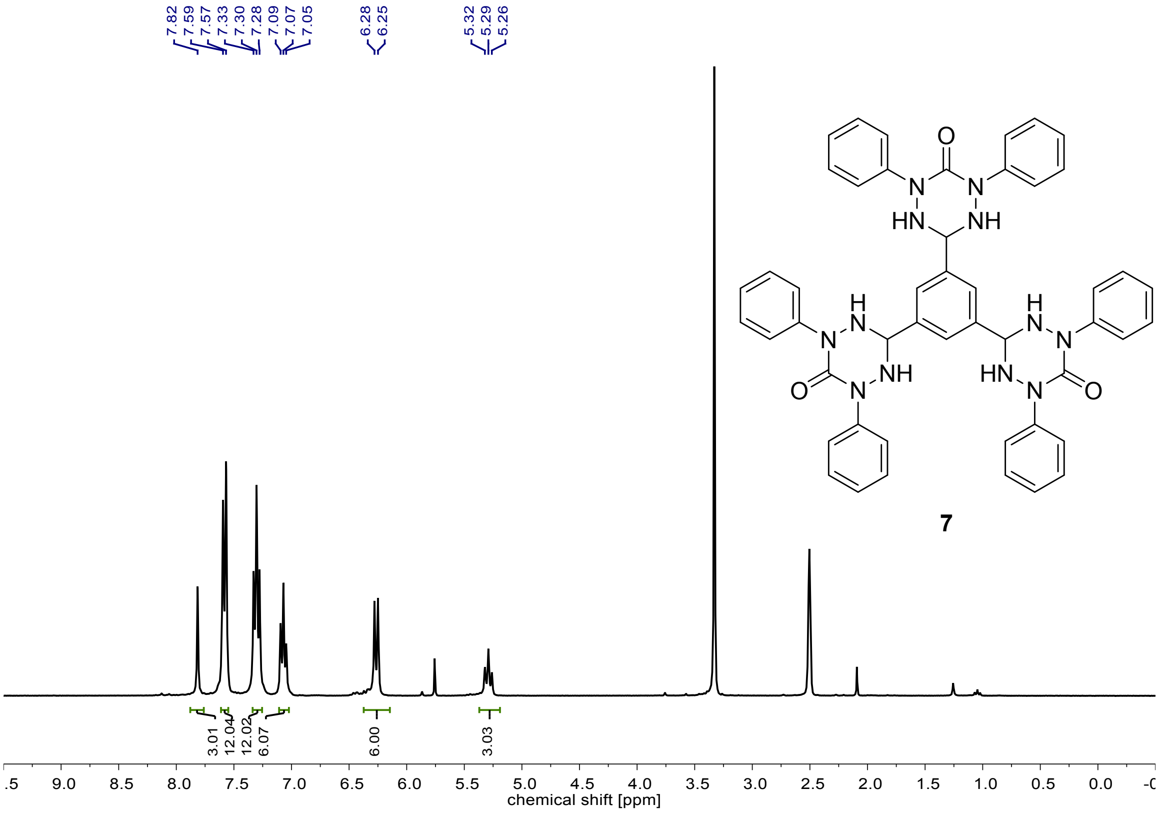
7.82
7.59
7.57
7.33
7.30
7.28
7.09
7.07
7.05
6.28
6.25
5.32
5.29
5.26
3.01
12.04
12.02
6.07
6.00
3.03
7
chemical shift [ppm]

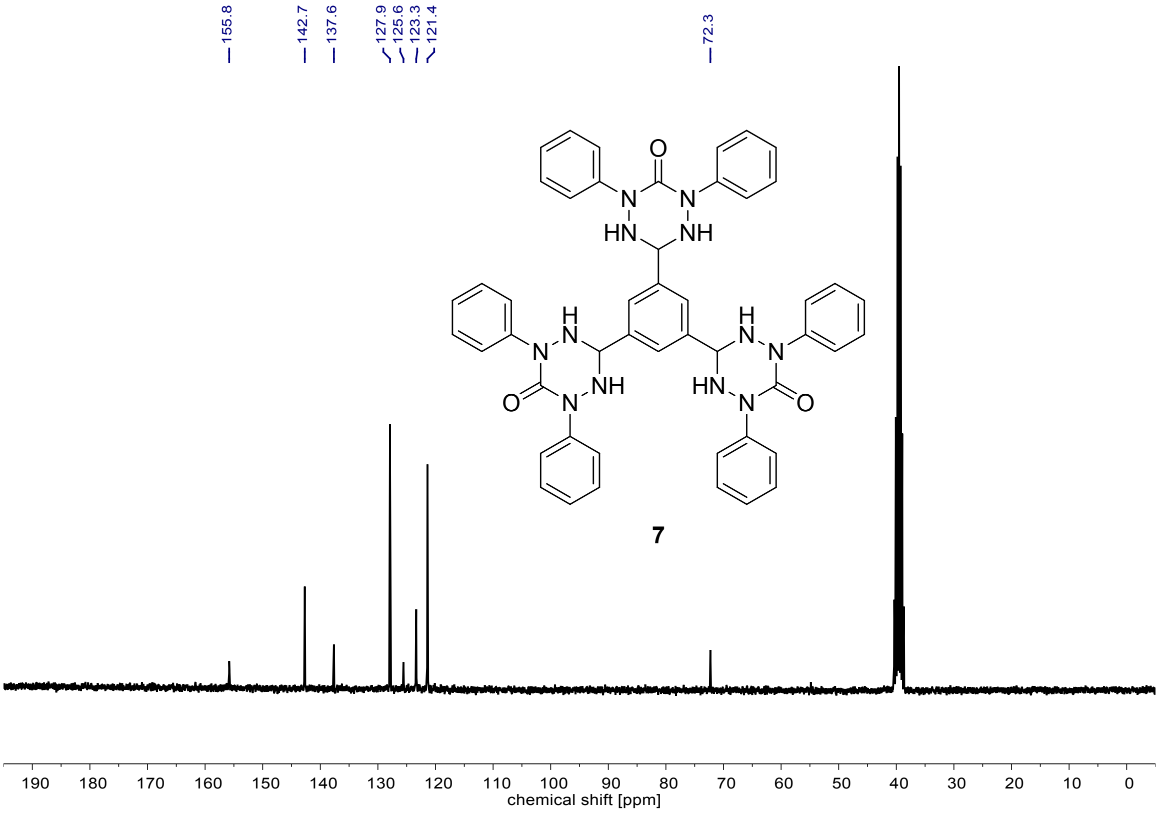
155.8
142.7
137.6
127.9
125.6
123.3
121.4
72.3
7
chemical shift [ppm]

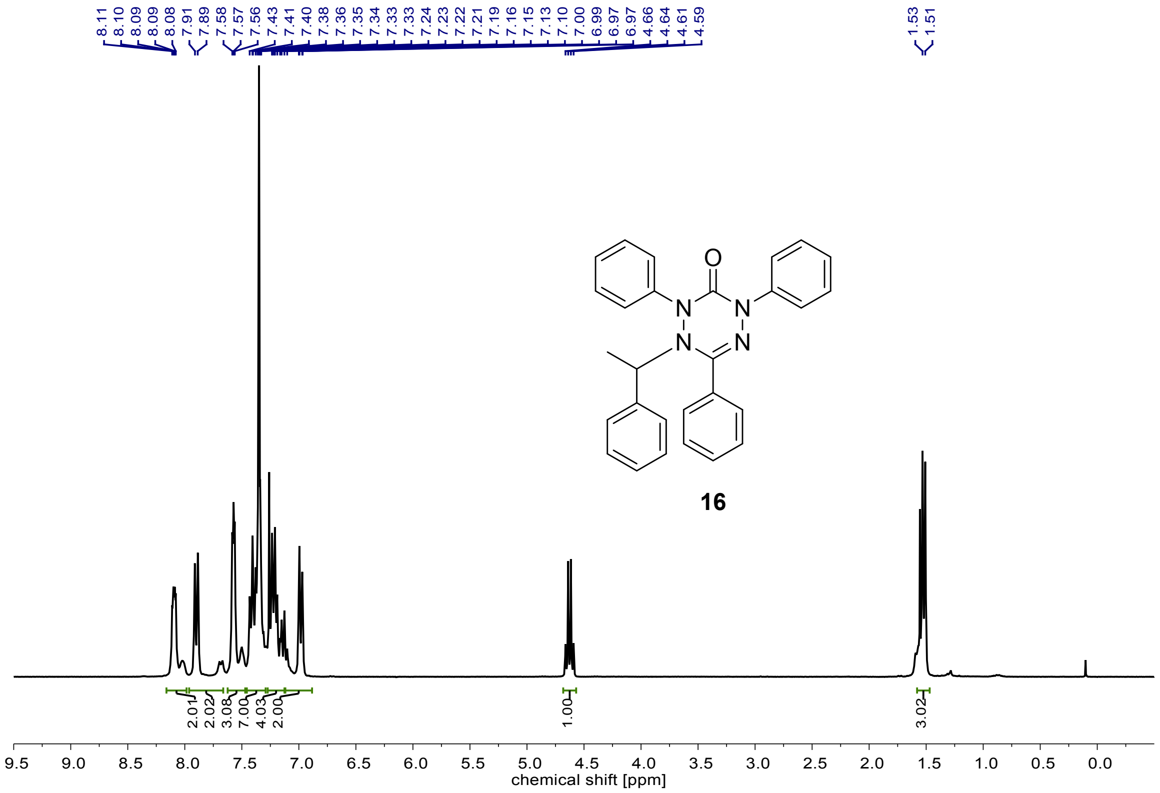
8.11
8.10
8.09
8.09
8.08
7.91
7.89
7.58
7.57
7.56
7.43
7.41
7.40
7.38
7.36
7.35
7.34
7.33
7.33
7.24
7.23
7.22
7.21
7.19
7.16
7.15
7.13
7.10
7.00
6.99
6.97
6.97
4.66
4.64
4.61
4.59
1.53
1.51
16
2.01
2.02
3.08
7.00
4.03
2.00
1.00
3.02
9.5
9.0
8.5
8.0
7.5
7.0
6.5
6.0
5.5
5.0
4.5
4.0
3.5
3.0
2.5
2.0
1.5
1.0
0.5
0.0
chemical shift [ppm]

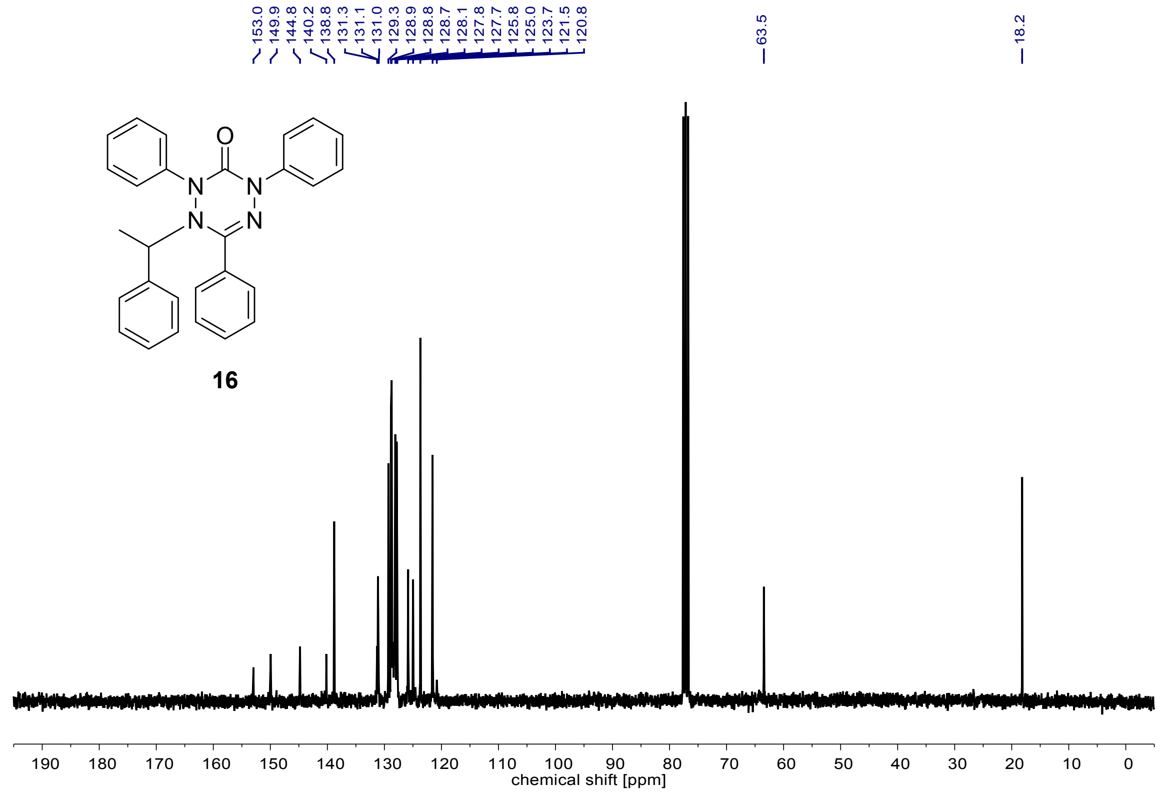
153.0
149.9
144.8
140.2
138.8
131.3
131.1
131.0
129.3
128.9
128.8
128.7
128.1
127.8
127.7
125.8
125.0
123.7
121.5
120.8
63.5
18.2
16
190
180
170
160
150
140
130
120
110
100
90
80
70
60
50
40
30
20
10
0
chemical shift [ppm]

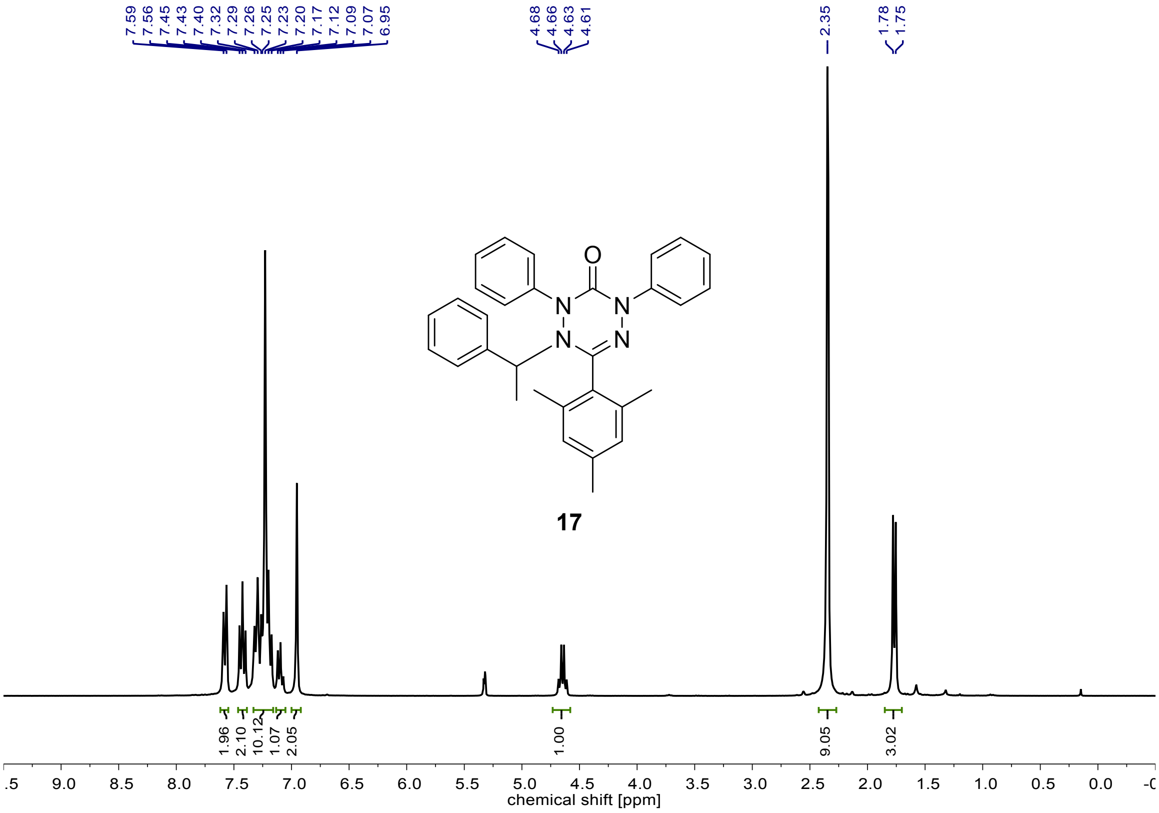
7.59
7.56
7.45
7.43
7.40
7.32
7.29
7.26
7.25
7.23
7.20
7.17
7.12
7.09
7.07
6.95
4.68
4.66
4.63
4.61
2.35
1.78
1.75
O
N
N
N
N
17
1.96
2.10
10.12
1.07
2.05
1.00
9.05
3.02
chemical shift [ppm]

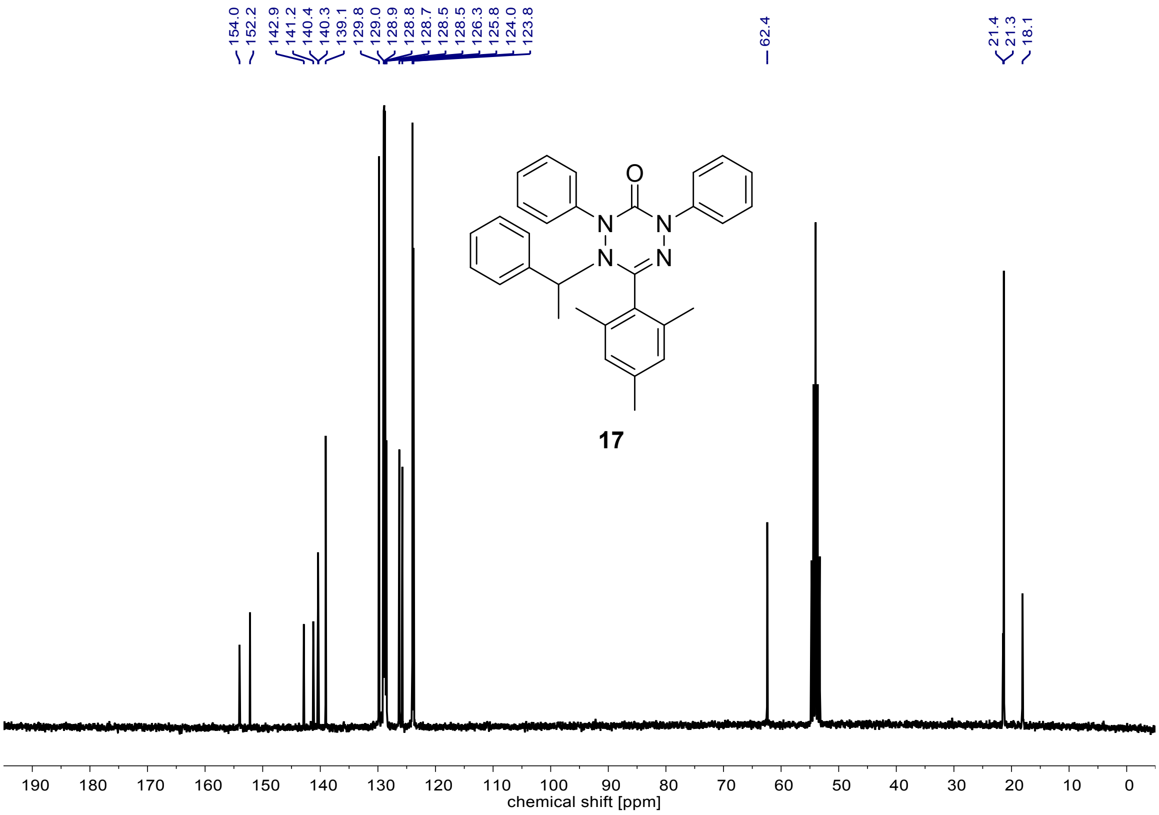
154.0
152.2
142.9
141.2
140.4
140.3
139.1
129.8
129.0
128.9
128.8
128.7
128.5
128.5
126.3
125.8
124.0
123.8
62.4
21.4
21.3
18.1
O
N
N
N
N
17
chemical shift [ppm]

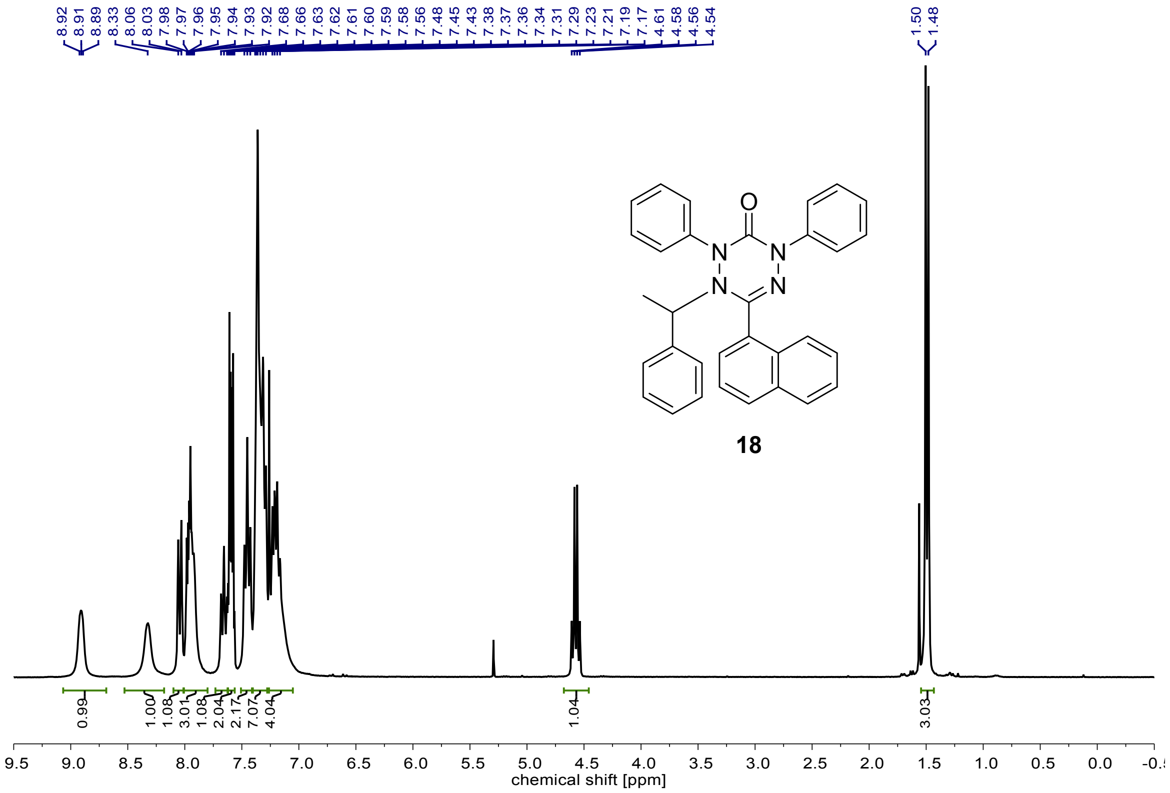
8.92 8.91 8.89 8.33 8.06 8.03 7.98 7.97 7.96 7.95 7.94 7.93 7.92 7.68 7.66 7.63 7.62 7.61 7.60 7.59 7.58 7.56 7.48 7.45 7.43 7.38 7.37 7.36 7.34 7.31 7.29 7.23 7.21 7.19 7.17 4.61 4.58 4.56 4.54
1.50 1.48
0.99 1.00 1.08 3.01 1.08 2.04 2.17 7.07 4.04
1.04
3.03
chemical shift [ppm]
18

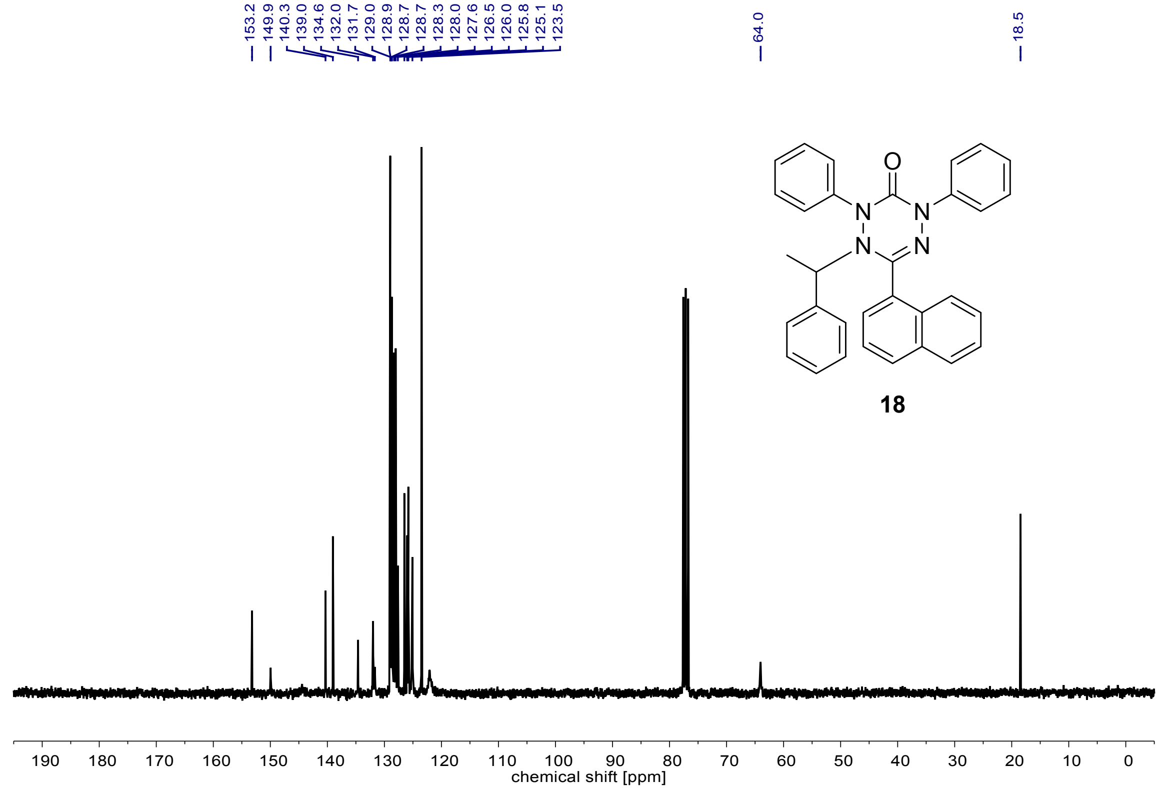
153.2 149.9 140.3 139.0 134.6 132.0 131.7 129.0 128.9 128.7 128.7 128.3 128.0 127.6 126.5 126.0 125.8 125.1 123.5
64.0
18.5
chemical shift [ppm]
18

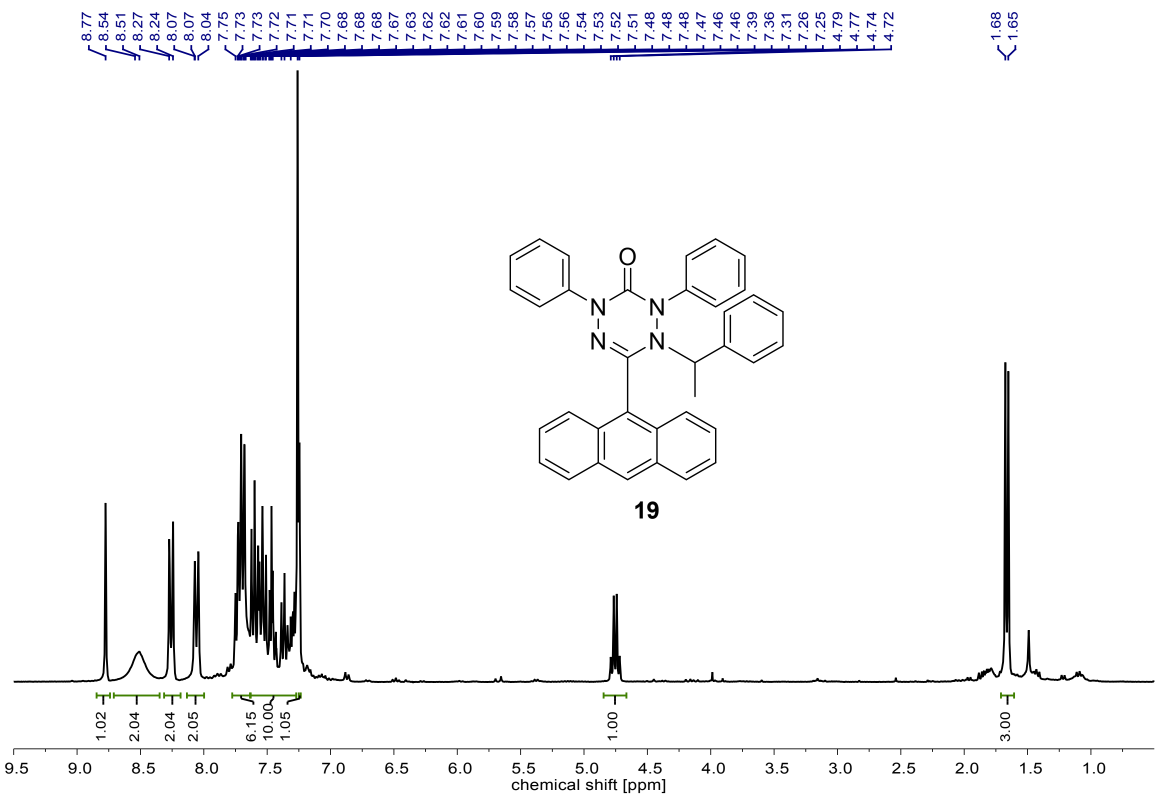
8.77 8.54 8.51 8.27 8.24 8.07 8.04 7.75 7.73 7.73 7.72 7.71 7.71 7.70 7.68 7.68 7.68 7.67 7.63 7.62 7.62 7.61 7.60 7.59 7.58 7.57 7.56 7.56 7.54 7.53 7.52 7.51 7.48 7.48 7.47 7.46 7.46 7.39 7.36 7.31 7.26 7.25 4.79 4.77 4.74 4.72
1.68 1.65
O
N
N
N
N
19
1.02 2.04 2.04 2.05 6.15 10.00 1.05 1.00 3.00
9.5 9.0 8.5 8.0 7.5 7.0 6.5 6.0 5.5 5.0 4.5 4.0 3.5 3.0 2.5 2.0 1.5 1.0
chemical shift [ppm]

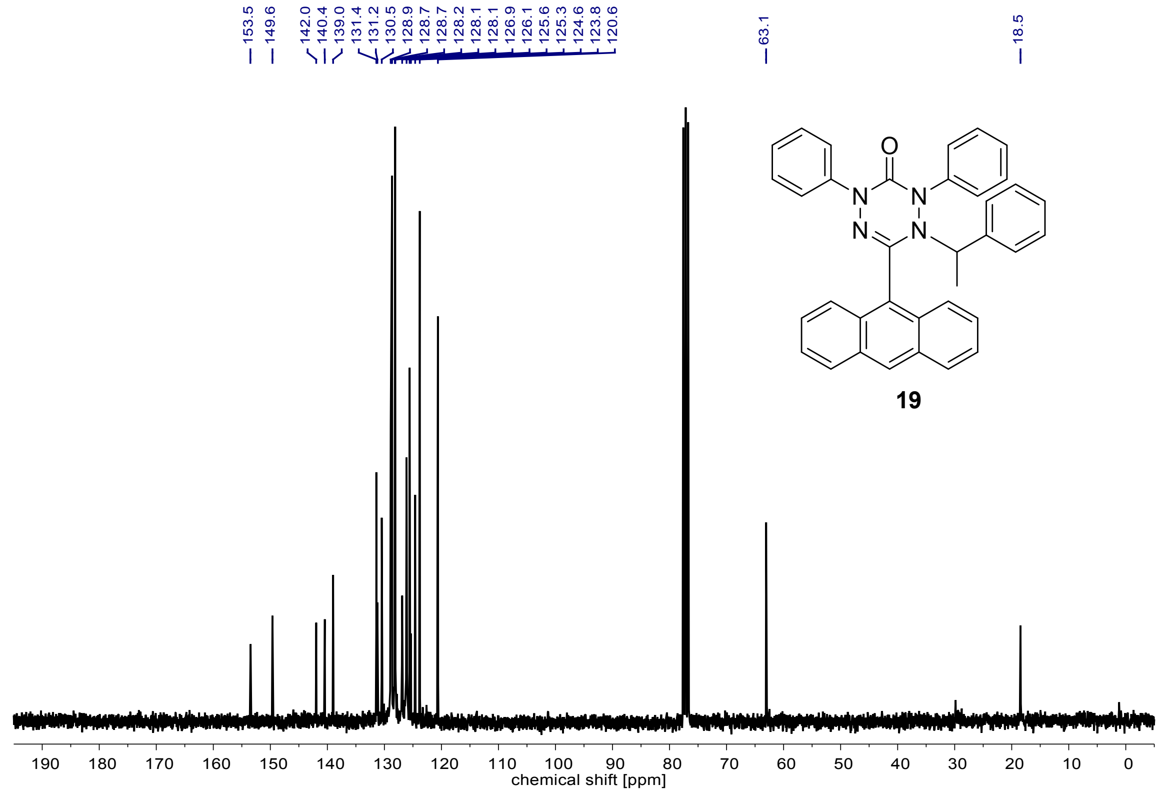
153.5 149.6 142.0 140.4 139.0 131.4 131.2 130.5 128.9 128.7 128.7 128.2 128.1 128.1 126.9 126.1 125.6 125.3 124.6 123.8 120.6
63.1
18.5
O
N
N
N
N
19
190 180 170 160 150 140 130 120 110 100 90 80 70 60 50 40 30 20 10 0
chemical shift [ppm]

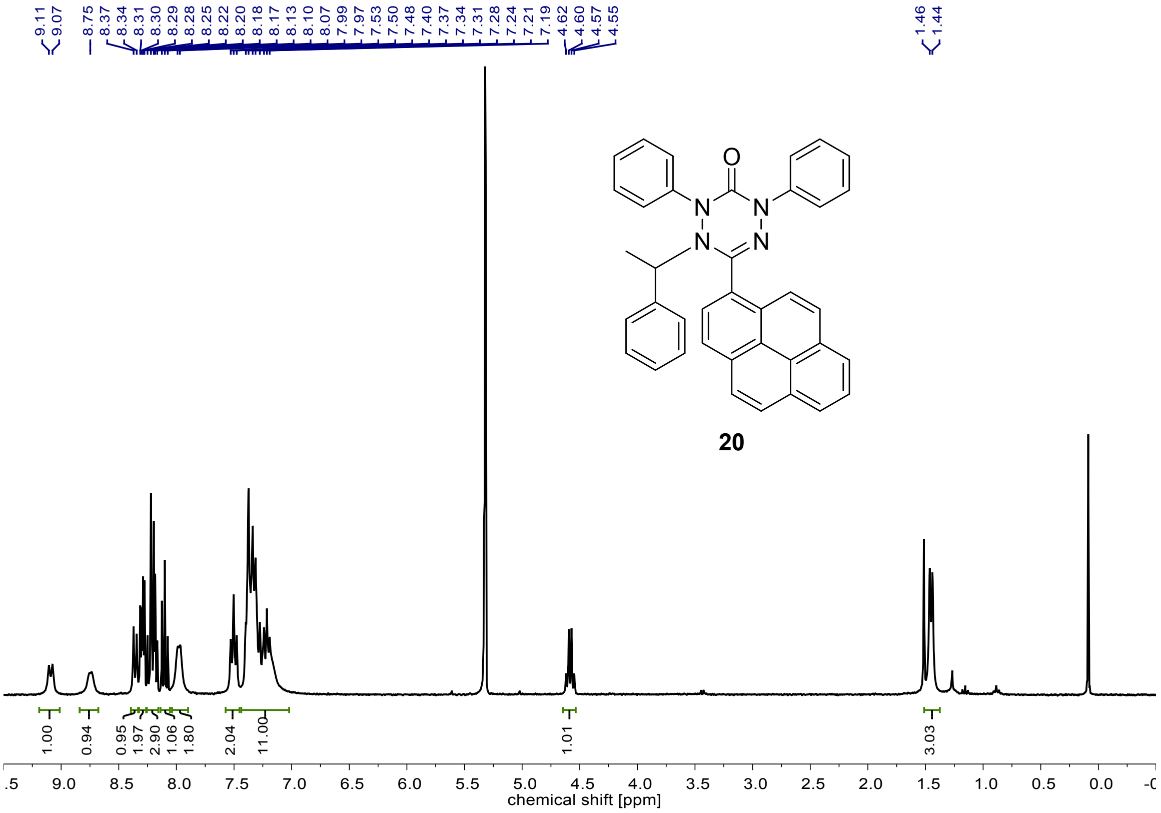

9.11
9.07
8.75
8.37
8.34
8.31
8.30
8.29
8.28
8.25
8.22
8.20
8.18
8.17
8.13
8.10
8.07
7.99
7.97
7.53
7.50
7.48
7.40
7.37
7.34
7.31
7.28
7.24
7.21
7.19
4.62
4.60
4.57
4.55
1.46
1.44
20
1.00
0.94
0.95
1.97
2.90
1.06
1.80
2.04
11.00
1.01
3.03
chemical shift [ppm]

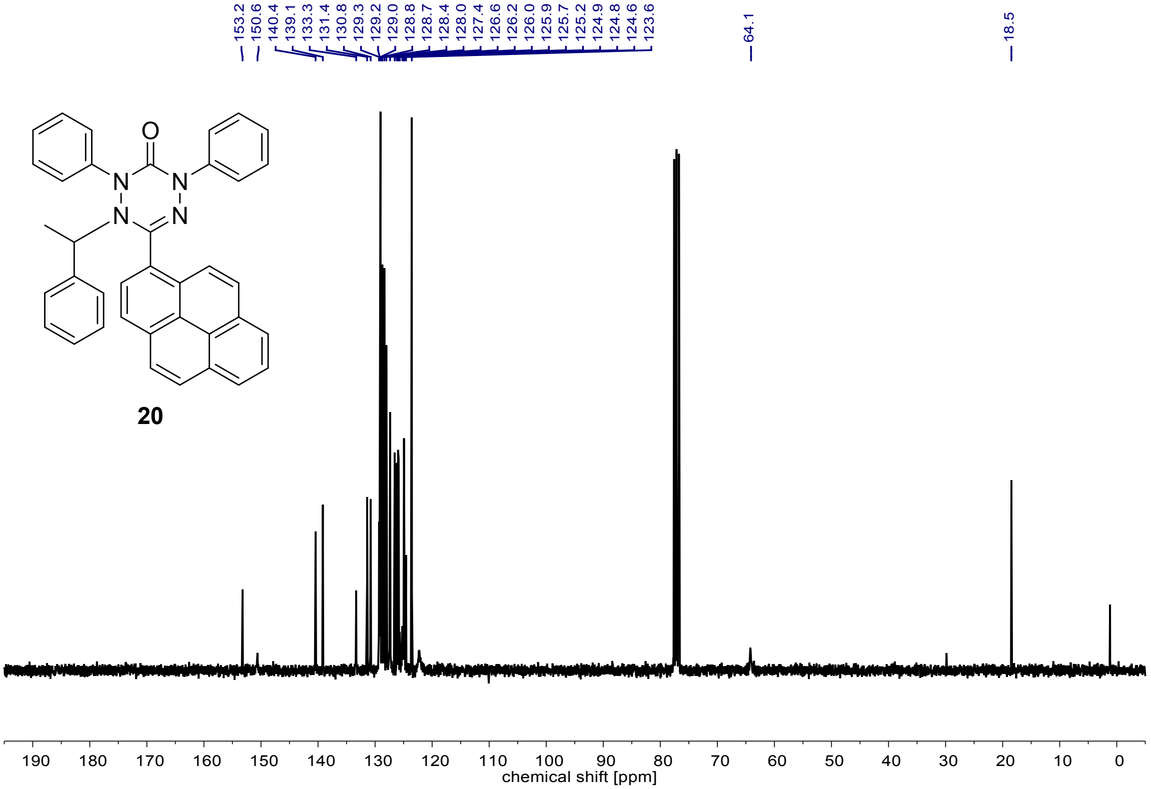

153.2
150.6
140.4
139.1
133.3
131.4
130.8
129.3
129.2
129.0
128.8
128.7
128.4
128.0
127.4
126.6
126.2
126.0
125.9
125.7
125.2
124.9
124.8
124.6
123.6
64.1
18.5
20
chemical shift [ppm]

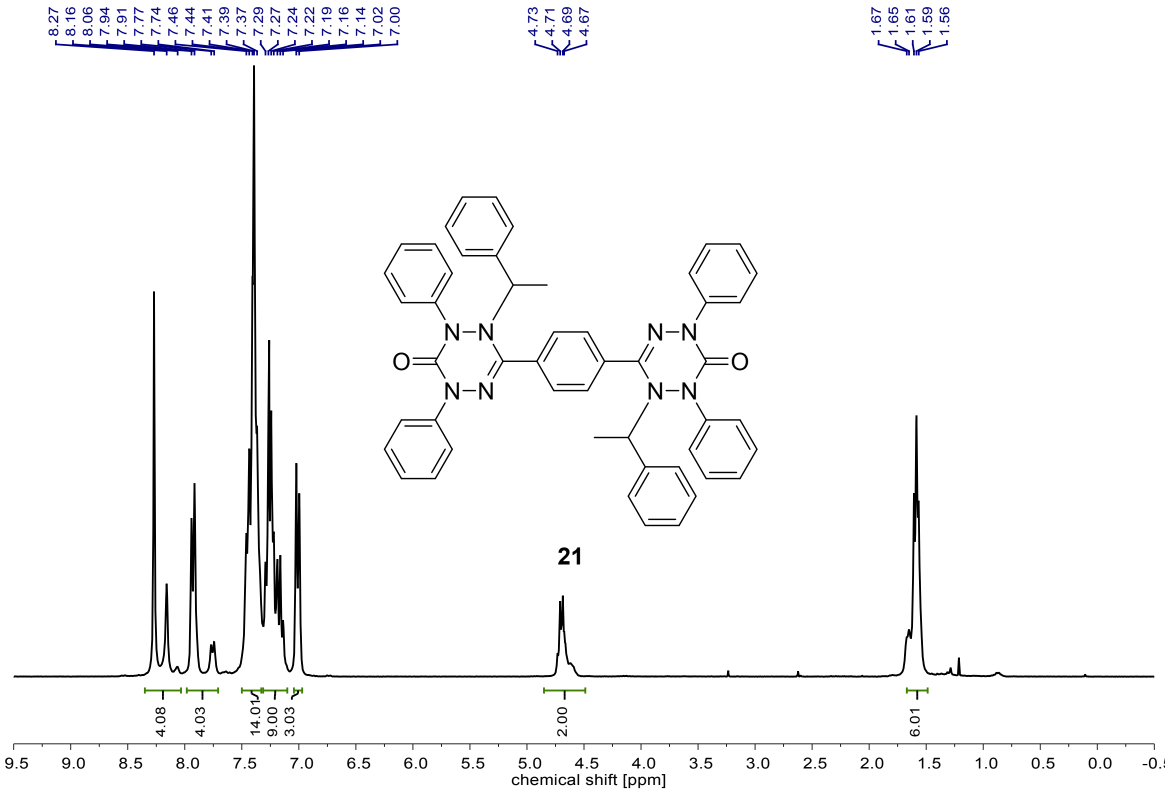
8.27
8.16
8.06
7.94
7.91
7.77
7.74
7.46
7.44
7.41
7.39
7.37
7.29
7.27
7.24
7.22
7.19
7.16
7.14
7.02
7.00
4.73
4.71
4.69
4.67
1.67
1.65
1.61
1.59
1.56
4.08
4.03
14.01
9.00
3.03
2.00
6.01
chemical shift [ppm]

21

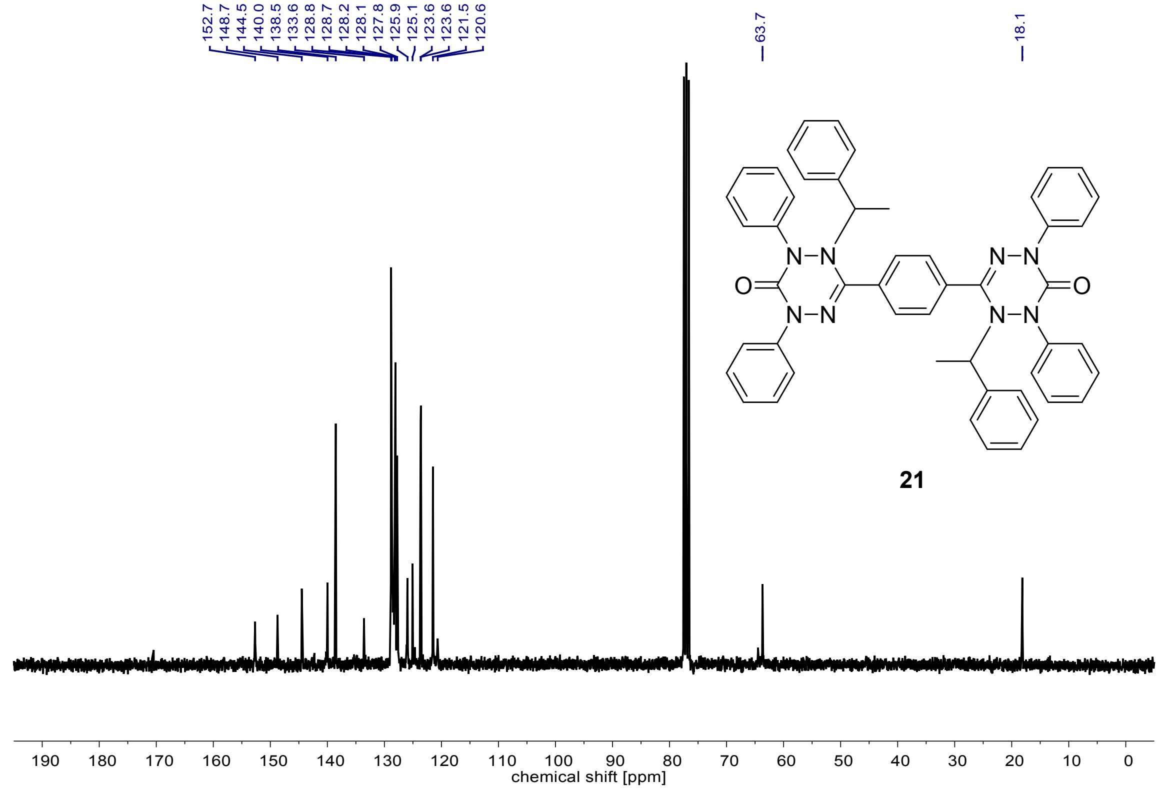
152.7
148.7
144.5
140.0
138.5
133.6
128.8
128.7
128.2
128.1
127.8
125.9
125.1
123.6
123.6
121.5
120.6
63.7
18.1
chemical shift [ppm]

21

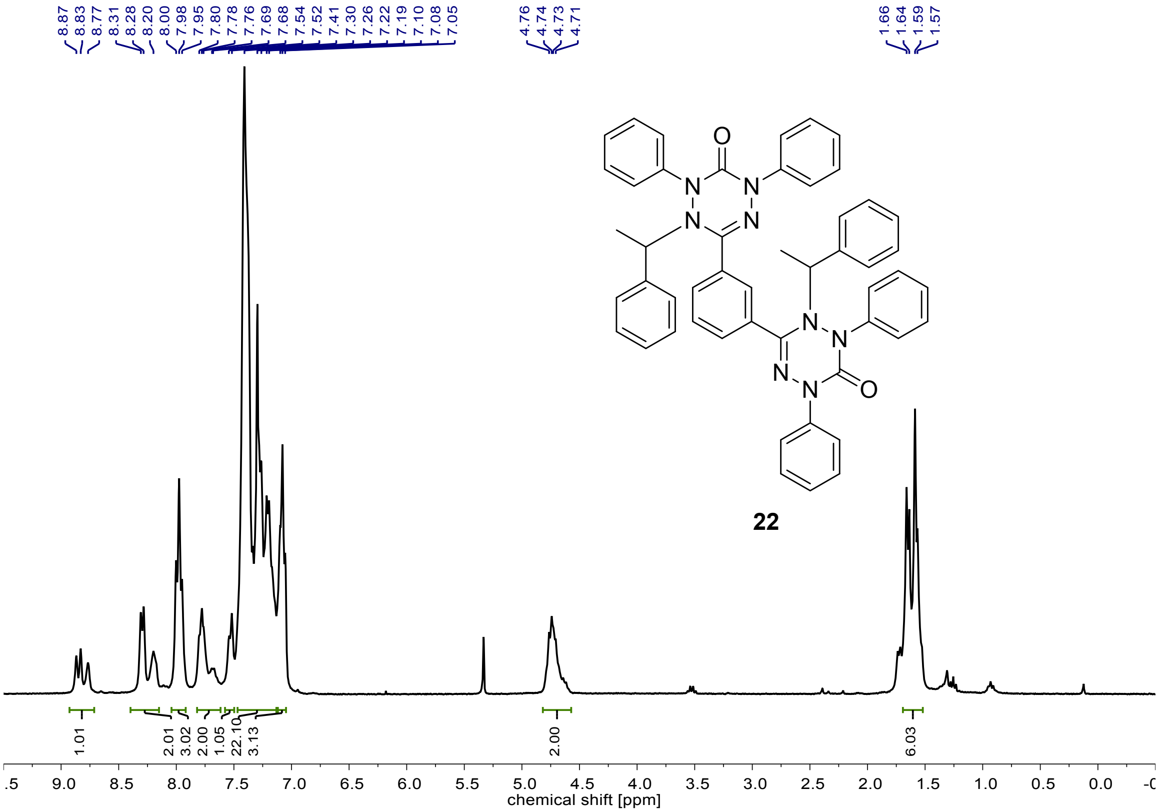
8.87 8.83 8.77 8.31 8.28 8.20 8.00 7.98 7.95 7.80 7.78 7.76 7.69 7.68 7.54 7.52 7.41 7.30 7.26 7.22 7.19 7.10 7.08 7.05
4.76 4.74 4.73 4.71
1.66 1.64 1.59 1.57
22
1.01 2.01 3.02 2.00 1.05 22.10 3.13 2.00 6.03
chemical shift [ppm]

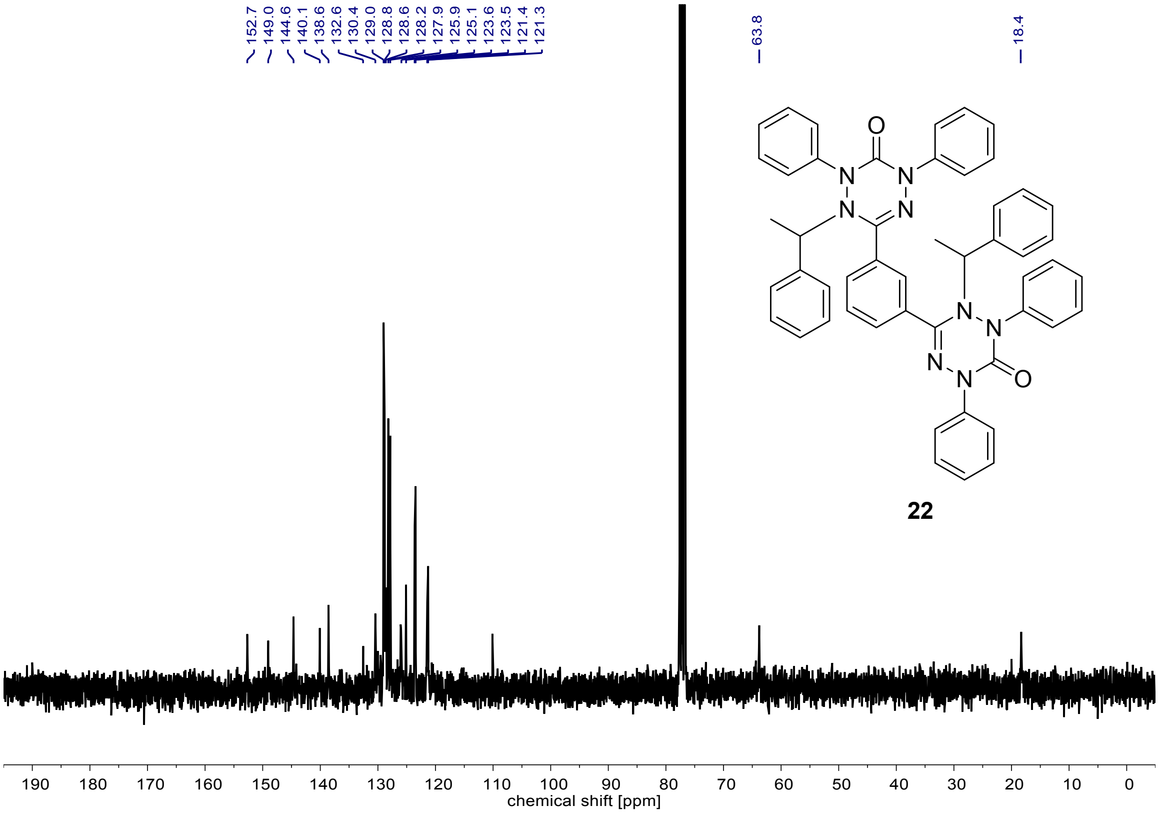
152.7 149.0 144.6 140.1 138.6 132.6 130.4 129.0 128.8 128.6 128.2 127.9 125.9 125.1 123.6 123.5 121.4 121.3
63.8
18.4
22
chemical shift [ppm]

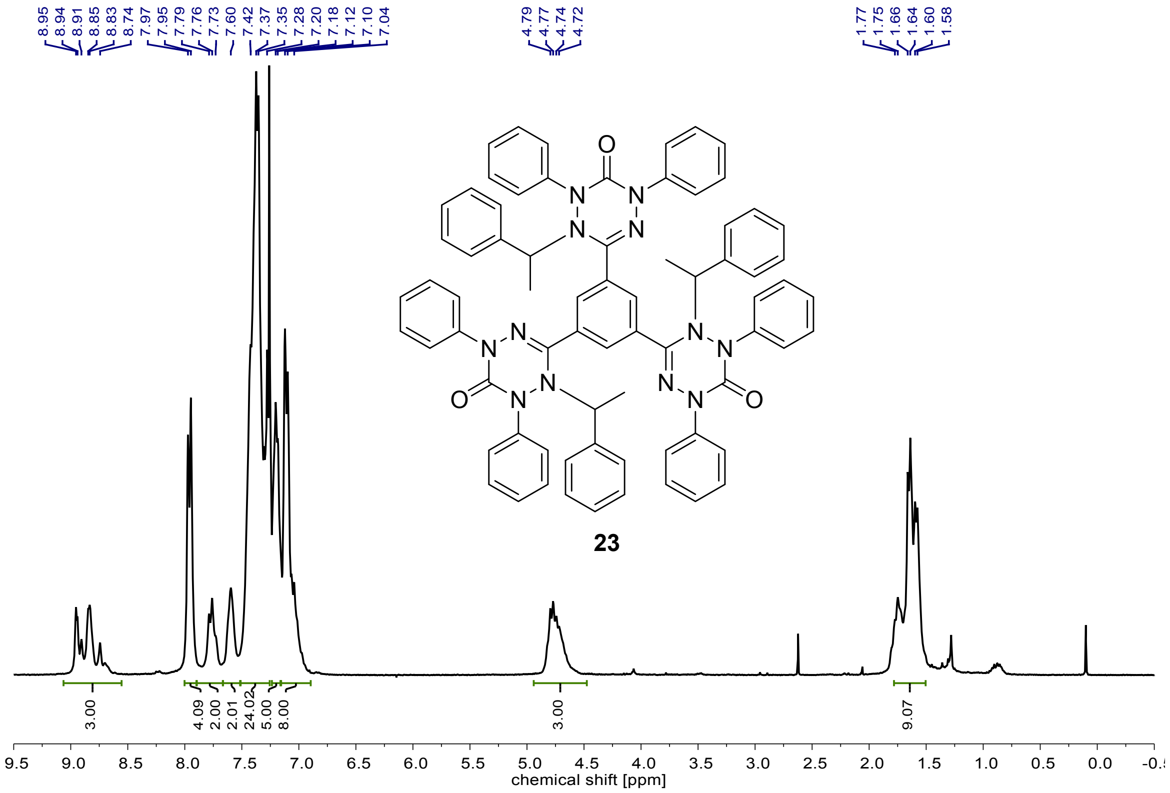
8.95 8.94 8.91 8.85 8.83 8.74 7.97 7.95 7.79 7.76 7.73 7.60 7.42 7.37 7.35 7.28 7.20 7.18 7.12 7.10 7.04
4.79 4.77 4.74 4.72
1.77 1.75 1.66 1.64 1.60 1.58
3.00 4.09 2.00 2.01 24.02 5.00 8.00 3.00 9.07
chemical shift [ppm]

23

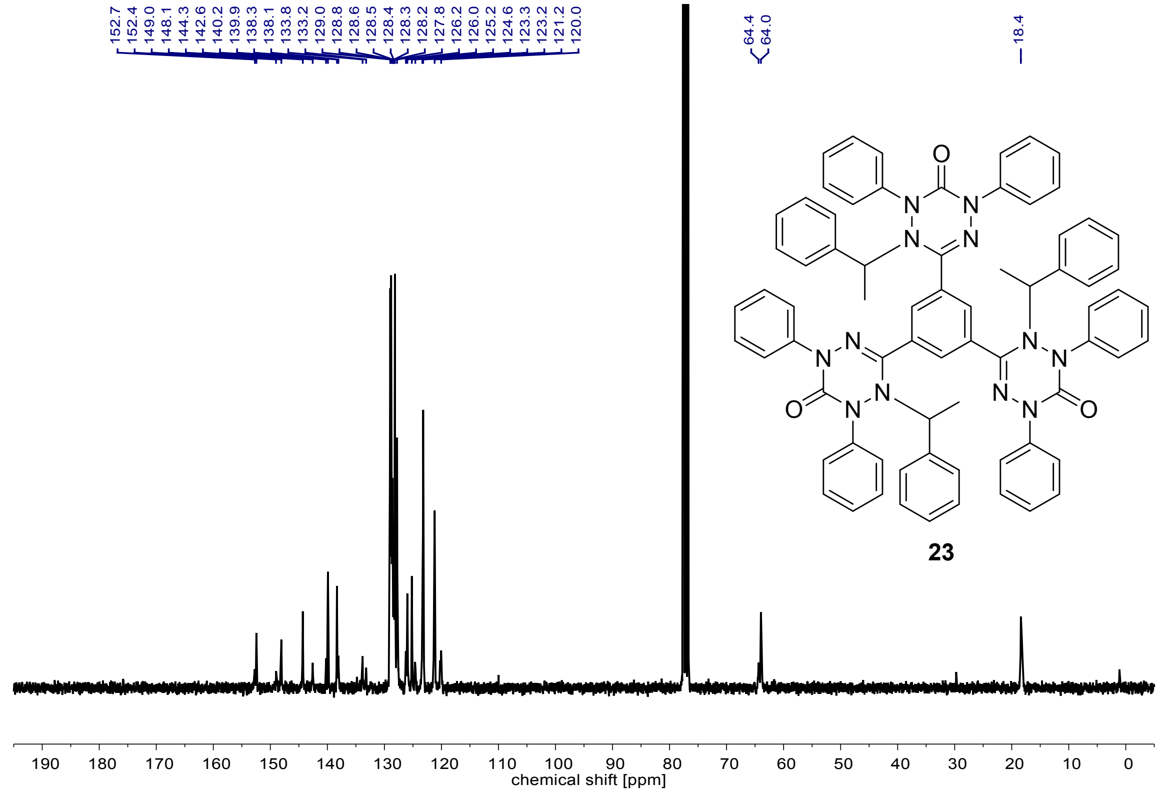
152.7 152.4 149.0 148.1 144.3 142.6 140.2 139.9 138.3 138.1 133.8 133.2 129.0 128.8 128.6 128.5 128.4 128.3 128.2 127.8 126.2 126.0 125.2 124.6 123.3 123.2 121.2 120.0
64.4 64.0
18.4
chemical shift [ppm]

23

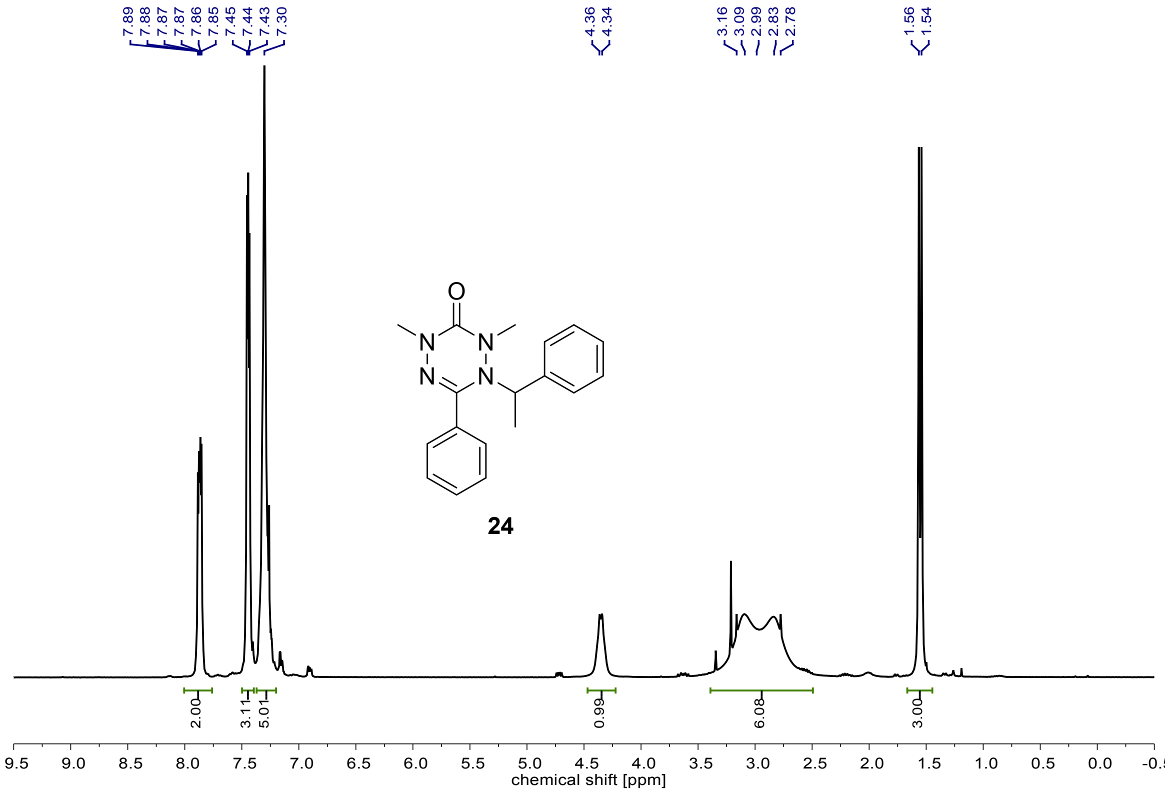
7.89
7.88
7.87
7.87
7.86
7.85
7.45
7.44
7.43
7.30
4.36
4.34
3.16
3.09
2.99
2.83
2.78
1.56
1.54
24
2.00
3.11
5.01
0.99
6.08
3.00
chemical shift [ppm]

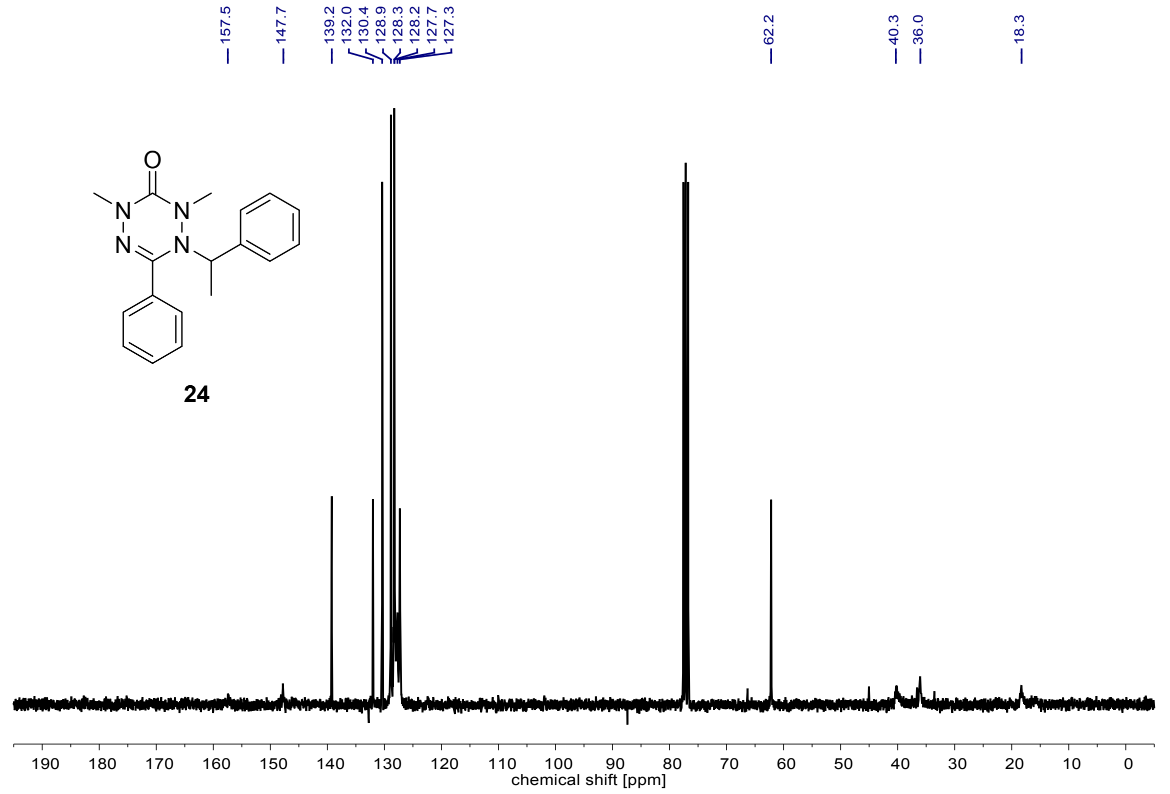
157.5
147.7
139.2
132.0
130.4
128.9
128.3
128.2
127.7
127.3
62.2
40.3
36.0
18.3
24
chemical shift [ppm]

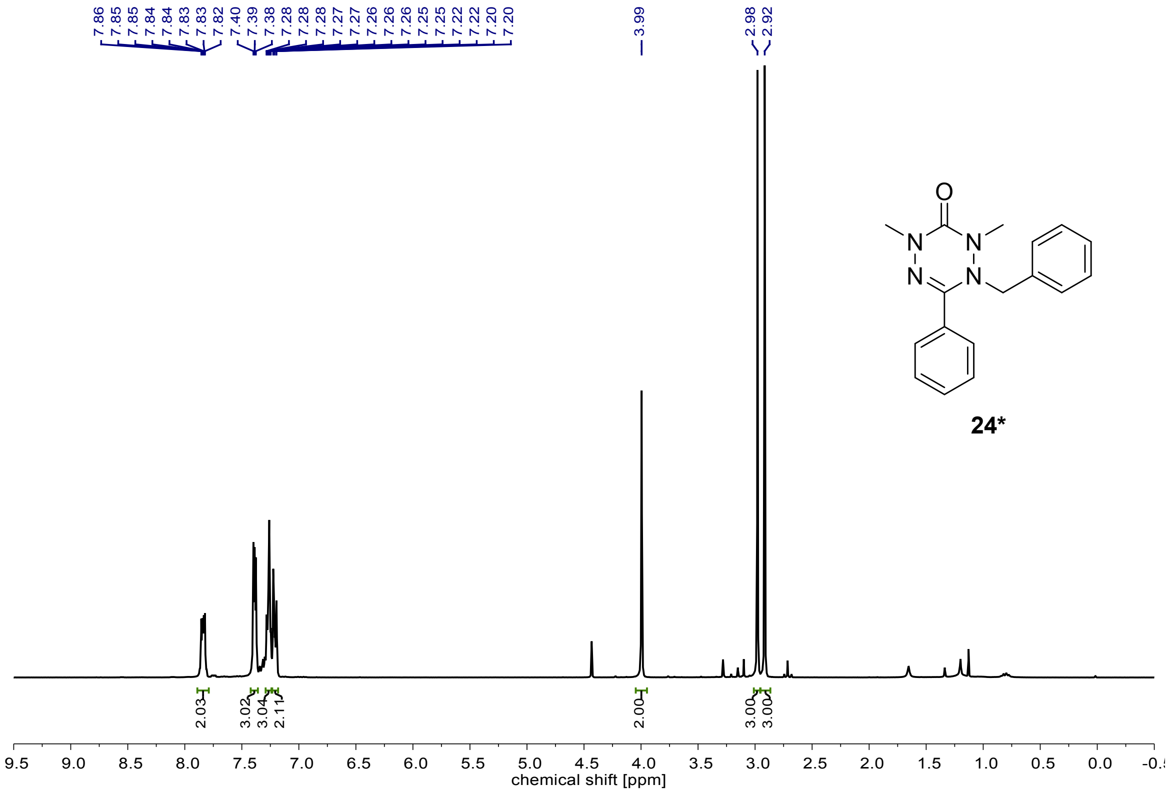
7.86 7.85 7.85 7.84 7.84 7.83 7.83 7.82 7.40 7.39 7.38 7.28 7.28 7.28 7.27 7.27 7.26 7.26 7.26 7.25 7.25 7.22 7.22 7.20 7.20
3.99
2.98 2.92
24*
2.03 3.02 3.04 2.11 2.00 3.00 3.00
chemical shift [ppm]

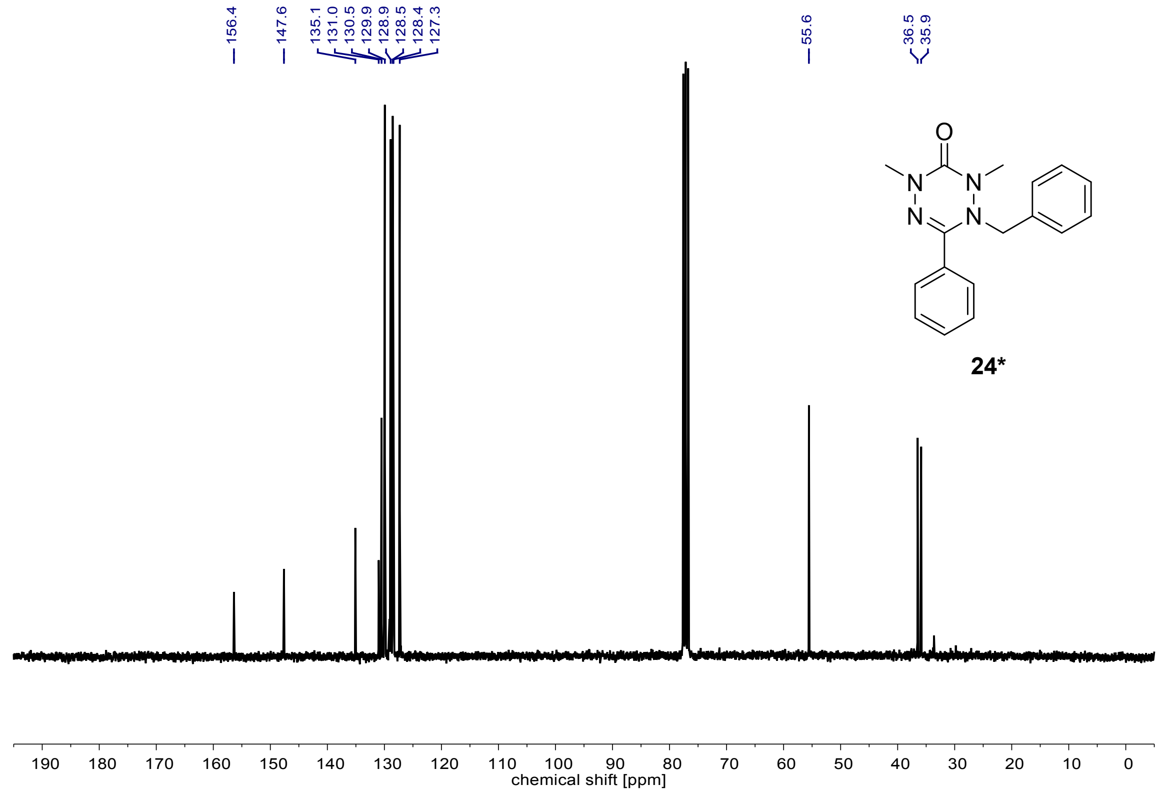
156.4
147.6
135.1 131.0 130.5 129.9 128.9 128.5 128.4 127.3
55.6
36.5 35.9
24*
chemical shift [ppm]

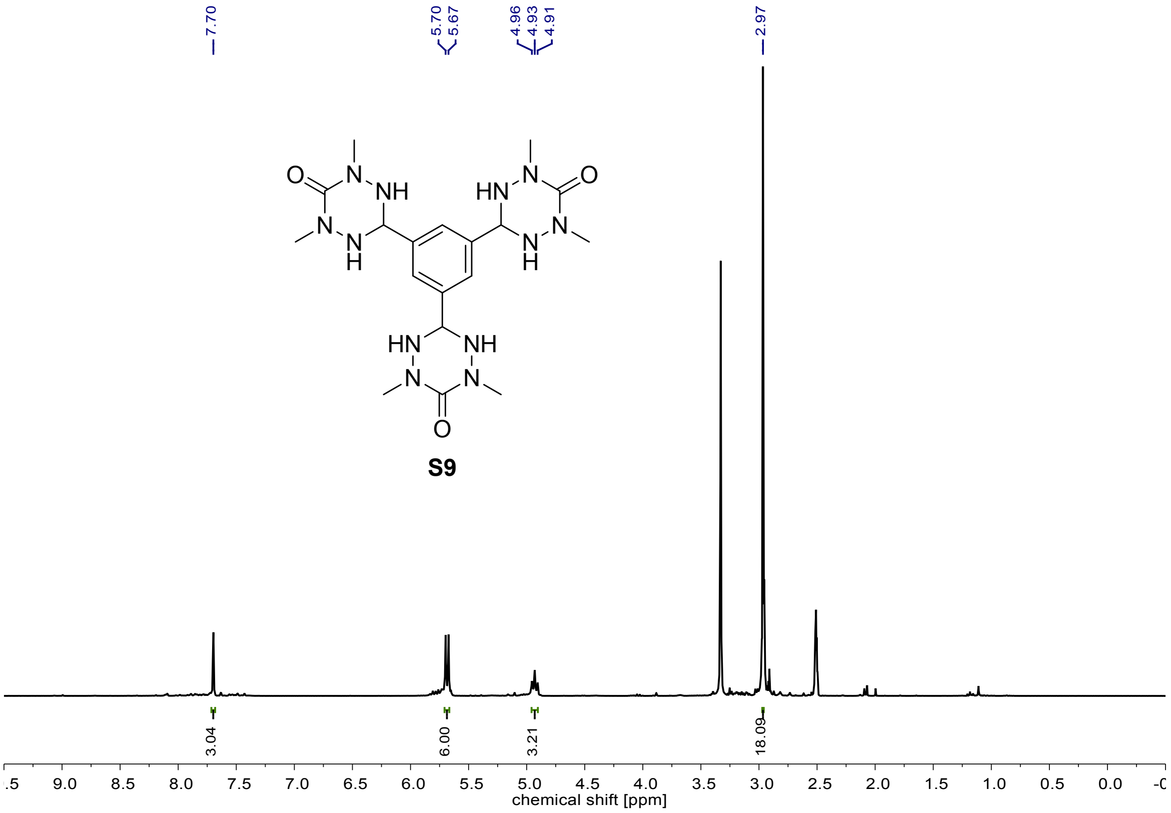
7.70
5.70
5.67
4.96
4.93
4.91
2.97
3.04
6.00
3.21
18.09
chemical shift [ppm]

S9

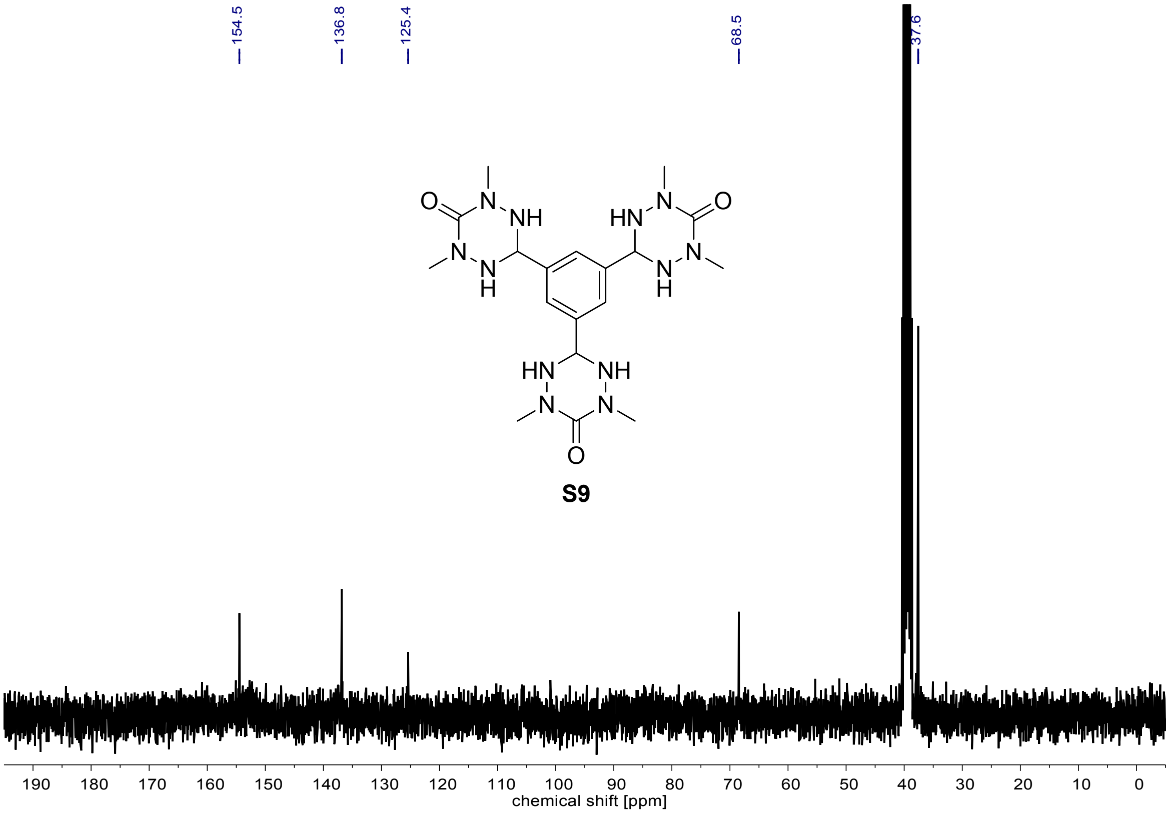
154.5
136.8
125.4
68.5
37.6
chemical shift [ppm]

S9

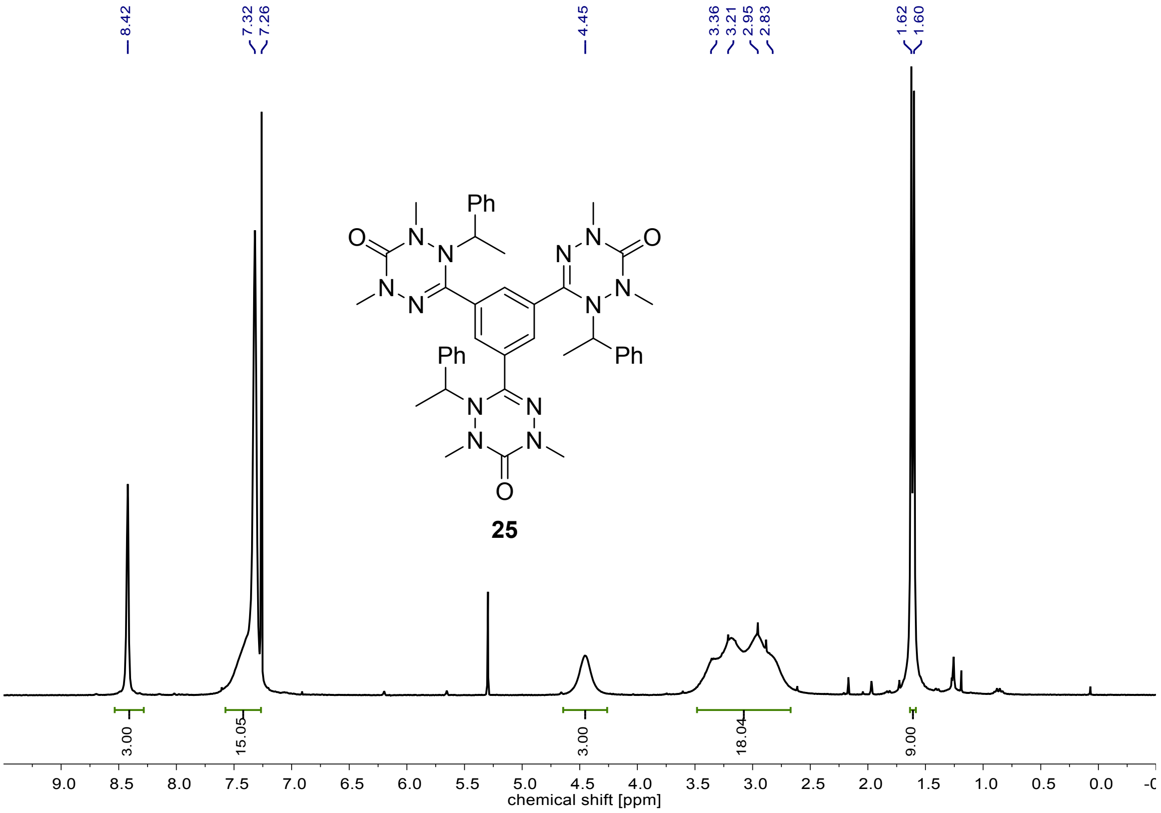
8.42
7.32
7.26
4.45
3.36
3.21
2.95
2.83
1.62
1.60
Ph
O
N
N
N
N
Ph
25
3.00
15.05
3.00
18.04
9.00
chemical shift [ppm]

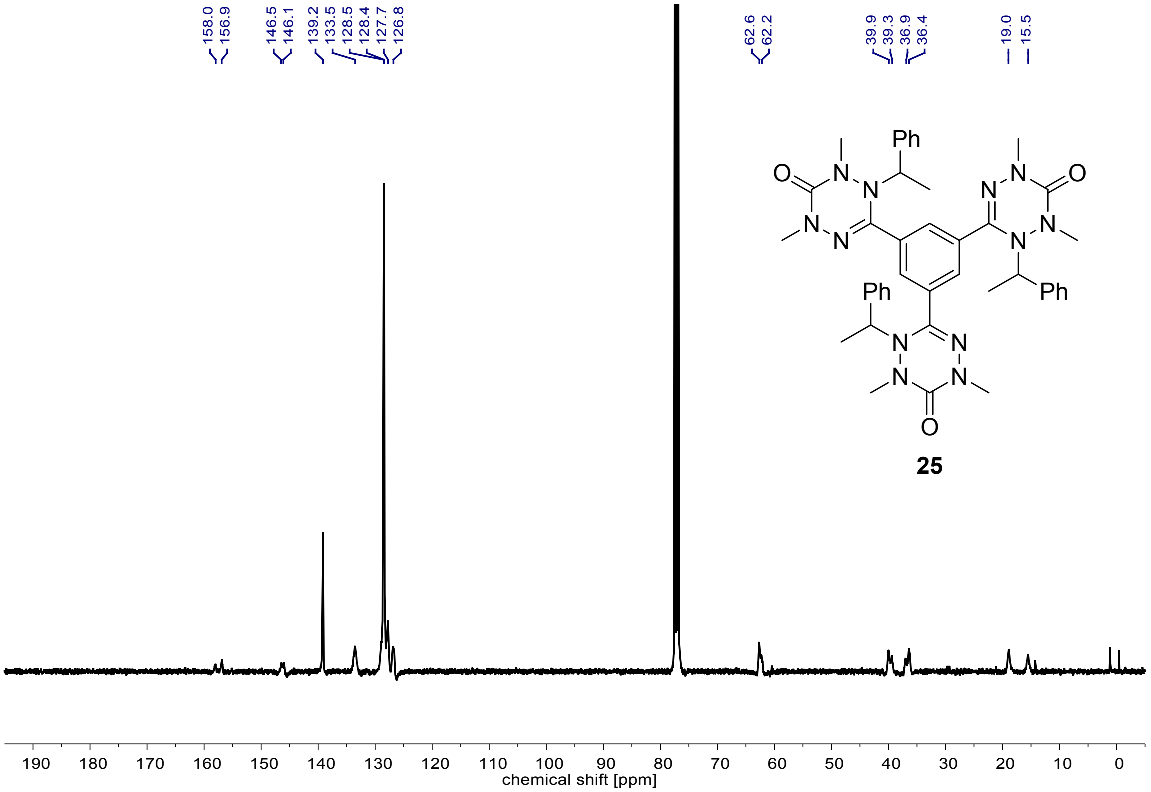
158.0
156.9
146.5
146.1
139.2
133.5
128.5
128.4
127.7
126.8
62.6
62.2
39.9
39.3
36.9
36.4
19.0
15.5
Ph
O
N
Ph
25
chemical shift [ppm]

8 References

1. A. M. Brouwer, *Pure Appl. Chem.*, 2011, **83**, 2213.
2. Y. Masuda, M. Kuratsu, S. Suzuki, M. Kozaki, D. Shiomi, K. Sato, T. Takui and K. Okada, *Polyhedron*, 2009, **28**, 1950.
3. F. A. Neugebauer, H. Fischer and R. Siegel, *Chem. Ber.*, 1988, **121**, 815.
4. K. Matyjaszewski, B. E. Woodworth, X. Zhang, S. G. Gaynor and Z. Metzner, *Macromolecules*, 1998, **31**, 5955.
5. A. Yang, T. Kasahara, E. K. Y. Chen, G. K. Hamer and M. K. Georges, *Eur. J. Org. Chem.*, 2008, 4571.
6. P. Pandey, A. P. Katsoulidis, I. Eryazici, Y. Wu, M. G. Kanatzidis and S. T. Nguyen, *Chem. Mater.*, 2010, **22**, 4974.
7. F. A. Neugebauer, H. Fischer and C. Krieger, *J. Chem. Soc., Perkin. Trans. 2*, 1993, 535.
8. M. Jasiński, J. S. Gerding, A. Jankowiak, K. Gębicki, J. Romański, K. Jastrzębska, A. Sivaramamoorthy, K. Mason, D. H. Evans, M. Celeda and P. Kaszyński, *J. Org. Chem.*, 2013, **78**, 7445.
9. M. J. Frisch *et al.* GAUSSIAN 09, Revision A.1, Gaussian, Inc., Pittsburgh PA, 2009.
10. C. Adamo and V. Barone, *J. Chem. Phys.*, 1999, **110**, 6158.
11. J. Hutter *et al.*, Car-Parrinello Molecular Dynamics: An Ab Initio Electronic Structure and Molecular Dynamics Program, see www.cpmd.org.
12. S. Nosé, *J. Chem. Phys.*, 1984, **81**, 511.
13. W. G. Hoover, *Phys. Rev.* , 1985, **A31**, 1695.
14. G. J. Martyna, M. L. Klein and M. E. Tuckerman, *J. Chem. Phys.*, 2002, **97**, 2635.
15. Z. Otwinowski and W. Minor, *Methods Enzymol.*, 1997, **276**, 307.
16. Z. Otwinowski, D. Borek, W. Majewski and W. Minor, *Acta Crystallogr.*, 2003, **A59**, 228.
17. G. M. Sheldrick, *Acta Crystallogr.*, 1990, **A46**, 467.
18. G. M. Sheldrick, *Acta Crystallogr.*, 2008, **A64**, 112.
